# Structures and stabilization of kinetoplastid-specific split rRNAs revealed by comparing leishmanial and human ribosomes

**DOI:** 10.1038/ncomms13223

**Published:** 2016-10-18

**Authors:** Xing Zhang, Mason Lai, Winston Chang, Iris Yu, Ke Ding, Jan Mrazek, Hwee L. Ng, Otto O. Yang, Dmitri A. Maslov, Z. Hong Zhou

**Affiliations:** 1 Center of Cryo Electron Microscopy, Zhejiang University School of Medicine, Hangzhou, Zhejiang 310058, China; 2 California NanoSystems Institute, University of California, Los Angeles, California 90095, USA; 3 Department of Microbiology, Immunology and Molecular Genetics, University of California, Los Angeles, California 90095, USA; 4 Division of Infectious Diseases, Department of Medicine, David Geffen School of Medicine, University of California Los Angeles, Los Angeles, California 90095, USA; 5 Department of Biology, University of California, Riverside, California 91521, USA

## Abstract

The recent success in ribosome structure determination by cryoEM has opened the door to defining structural differences between ribosomes of pathogenic organisms and humans and to understand ribosome-targeting antibiotics. Here, by direct electron-counting cryoEM, we have determined the structures of the *Leishmania donovani* and human ribosomes at 2.9 Å and 3.6 Å, respectively. Our structure of the leishmanial ribosome elucidates the organization of the six fragments of its large subunit rRNA (as opposed to a single 28S rRNA in most eukaryotes, including humans) and reveals atomic details of a unique 20 amino acid extension of the uL13 protein that pins down the ends of three of the rRNA fragments. The structure also fashions many large rRNA expansion segments. Direct comparison of our human and leishmanial ribosome structures at the decoding A-site sheds light on how the bacterial ribosome-targeting drug paromomycin selectively inhibits the eukaryotic *L. donovani*, but not human, ribosome.

## Abstract (web summary)

Leishmania donovani is a protozoan parasite that can cause fatal infections in humans. Here the authors present a high resolution cryoEM structure of the *L. donovani* 80S ribosome and compare it to its human counterpart to provide insight into the basis for drug selectivity towards this eukaryotic parasite.

## Graphical abstract

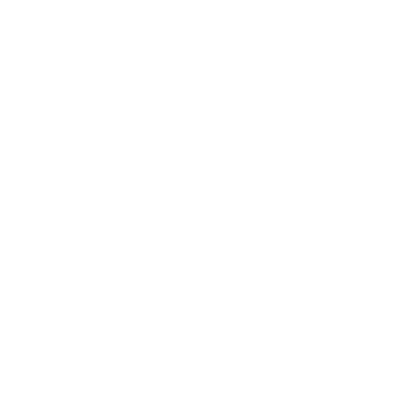

L*eishmania donovani* is a protozoan parasite responsible for ∼400,000 new cases of visceral leishmaniasis each year^1^. This disseminated infection carries about 10% mortality with treatment, and is almost uniformly fatal if left untreated. Treatment options are limited, and include drugs with significant toxicities, further complicated by the rapid emergence of resistant Leishmania strains, necessitating a better understanding of drug targets. The World Health Organization recommends a broad-spectrum, bacterial ribosome-targeting drug, paromomycin, for treatment of visceral leishmaniasis^1^. However, the selectivity of this prokaryotic ribosome inhibitor for the *L. donovani* versus human ribosome is poorly understood in the absence of a systematic comparison of the atomic structures of these ribosomes.

Recent technological advances in cryo electron microscopy (cryoEM) have allowed for atomic modelling of large asymmetrical particles such as the ribosome. In the wake of these advancements, the field of ribosome studies has seen an explosion of high-resolution cryoEM ribosome structures^2^,^3^,^4^,^5^,^6^,^7^, presenting a remarkable opportunity to define the unique structures of components of the *L. donovani* ribosome, which should provide valuable information about kinetoplastid ribosome biogenesis and may serve as the basis for future identification of antibiotic targets. Given the large evolutionary distance between *Leishmania* and related trypanosomes versus humans, one would expect the *Leishmania* ribosomal structure to open novel avenues through which antibiotic selectivity could be explored and new more efficient inhibitors designed. Indeed, unlike the single molecule in humans, the 28S ribosomal RNA (rRNA) in the large subunit (LSU) of the *L. donovani* ribosome is split into six separate molecules^8^. These rRNA molecules require involvement of kinetoplastid-specific proteins for ribosome biogenesis^9^, and interact closely with one another and with trypanosome-unique protein extensions for ribosome integrity^10^. However, no high-resolution structure is available of a leishmanial ribosome, and the only existing trypanosomal ribosome structures are that of *Trypanosoma brucei* at 5.57 Å resolution^3^ and that of *Trypanosoma cruzi* at 12 Å resolution^11^, both of which lack atomic details of protein–RNA and RNA–RNA interactions needed for understanding the stabilization of fragmented rRNA and for describing chemical specificity of ribosome-targeting drugs.

Here, we have used cryoEM to reconstruct the ribosome structures of *L. donovani* and *Homo sapiens* at 2.9 Å and 3.6 Å, respectively, and derive their atomic models. Comparison of the atomic model of the leishmanial ribosome with that of the human ribosome reveals RNA–protein interactions formed among the ends of the split rRNA molecules, as well as significant differences in the arrangement and presence of ribosomal RNA expansion segments, and base-pair features at the decoding A-site relevant to selective inhibition by paromomycin.

## Results

### Overall structure of the leishmanial ribosome

**Figure 1:**
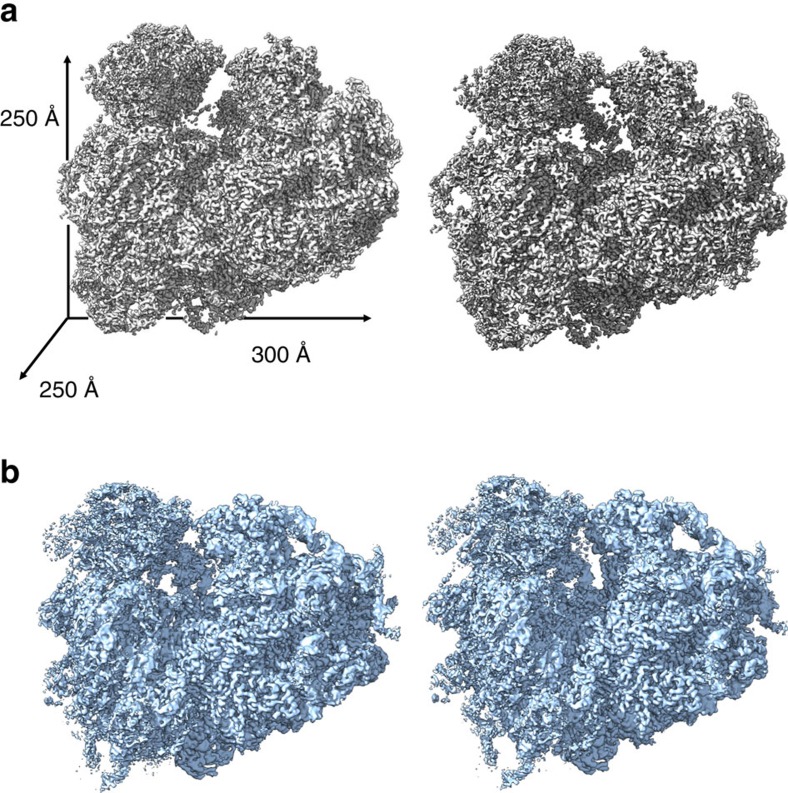
CryoEM reconstructions of the *L. donovani* and human ribosomes. Stereo related views of the *L. donovani* (**a**) and human (**b**) ribosomes at 2.9 Å and 3.6 Å respectively. Approximate dimensions of the ribosome: 300 Å × 250 Å × 250 Å.

We recorded cryoEM images of *L. donovani* 80S ribosomes on a direct electron counting camera, performed two-dimensional (2D) image classification and three-dimensional (3D) structure classification, and, taking into consideration possible ratcheting movement between the large subunit (LSU) and small subunit (SSU) subunits, performed both global and focused refinements (refining the ribosome as a whole or refining LSU and SSU separately, see Methods) of the 3D structures. We found very little ratcheting movement between LSU and SSU in our sample. Our best 3D structure was obtained with global refinement and has an averaged resolution of 2.9 Å (Fig. 1, Supplementary Fig. 1; Supplementary Table 1). Consistent with this resolution assessment, our reconstructions resolved many side chains of amino acids and bases of nucleic acids (Supplementary Figs 1–6, Supplementary Movies 1–5), which were used to build atomic models for the protein and RNA molecules (Supplementary Tables 2–3).

We follow the recommended nomenclature for rRNA molecules as established by Gerbi^12^ and protein components as established by Ban *et al*.^13^. Specifically, rRNA Expansion Segments were identified by numbering followed by a letter designating the Small or Large subunit (for example, ES6S designates expansion segment six of the small subunit); protein components by a prefix designating its eukaryotic, bacterial or universal nature followed by a letter indicating protein's attribution to the Small or Large subunit, and a number (for example, uL13 designates universal protein 13 in the large subunit).

**Figure 2:**
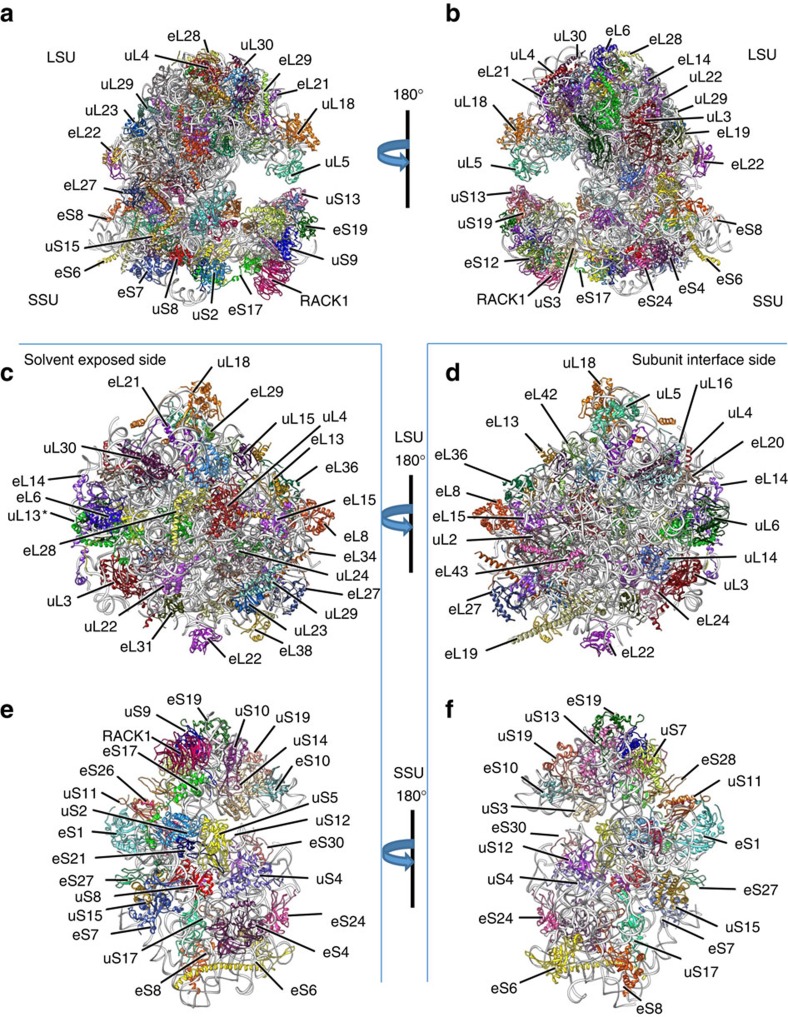
Arrangement of the *L. donovani* ribosome. Atomic models of the whole ribosome (**a**,**b**), LSU (**c**,**d**), and SSU (**e**,**f**) of the *L. donovani* 80S ribosome. Protein molecules are shown in colour and rRNA molecules in grey. (**c**)The LSU as seen from the side exposed to solvent (Solvent exposed side). (**d**) The LSU as seen from the subunit interface (Subunit interface side). (**e**) The SSU as seen from the side exposed to solvent (Solvent exposed side). (**f**) The SSU as seen from the subunit interface (Subunit interface side). *Protein uL13 is involved in rRNA stabilization, binding the ends of srRNA2 and srRNA3.

For the LSU (Supplementary Table 2), we were able to model 41 proteins (including uL2-6, uL13-16, uL18, uL22-24, uL29-30, eL6, eL8, eL13-15, eL18-22, eL24, eL27-34, eL36-40 and eL42-43 (ref. 13)) and eight RNA molecules (including ES7L, ES31L, ES19L, ES3L, ES27L and ES4L), but not uL1 or uL11.The N-terminal or C-terminal tails of several proteins were also not clearly resolved. In the SSU (Supplementary Table 3), 30 proteins (including uS2-5, uS7, uS8-15, uS17, uS19; eS1, eS4, eS6-8, eS10, eS17, eS19, eS21, eS24, eS26-28, eS30 and RACK1 (ref. 13)) and the 18S rRNA (including expansion segments ES6S, ES7S, ES3S, ES2S, ES12S, ES10S and ES9S) were modelled (Fig. 2), while eS12, eS31 and eS25 were not, although their secondary structures were resolved. The validities of our cryoEM map and atomic model are supported by the excellent match of the observed density with amino acid side chains and nucleic acid bases (Supplementary Figs 1-6), and only 0.93% and 2.93% of residues categorized as Ramachandran outliers in the LSU and SSU, respectively (Supplementary Table 1).

**Figure 3:**
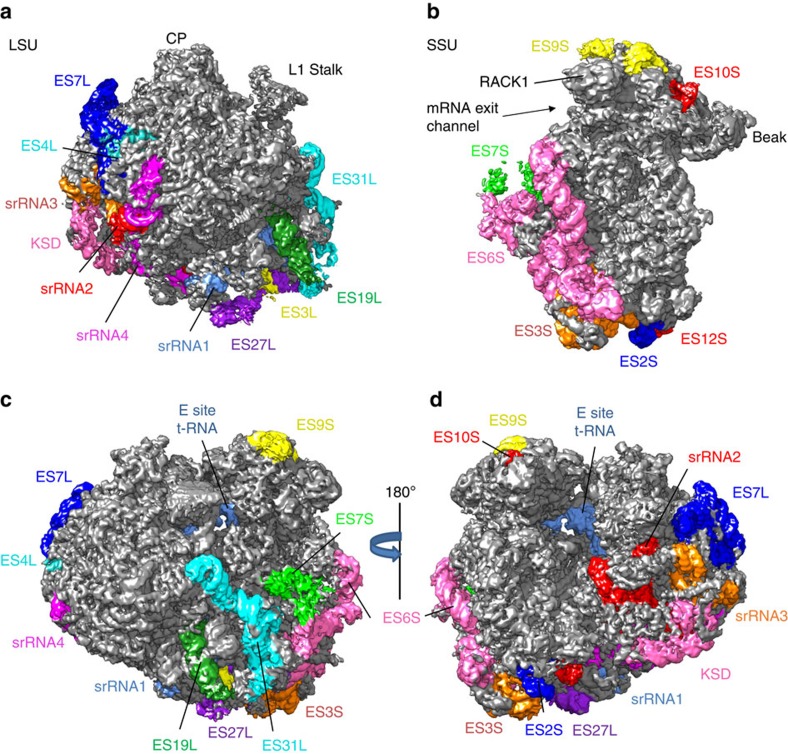
Leishmanial ribosome expansion segments that are distinctive from non-trypanosomal ribosomes. For clarity, the cryoEM map of the *L. donovani* ribosome was filtered to a resolution of 5 Å with the most conserved structure elements displayed in grey and the expansion segments distinctive from those in non-trypanosomal ribosomes in colour. (**a**,**b**) The solvent-exposed side (side visible from the exterior of the ribosome) of LSU (**a**) and SSU (**b**). (**c**,**d**) Whole ribosome with visible tRNA in the E site. CP, central protuberance.

Overall, the structure of *L. donovani* 80S ribosome shows a combination of conserved and unique features. In addition to the core region (indicated with grey in Fig. 3) characteristic of 80S ribosomes^2^,^3^,^4^,^5^,^7^,^14^,^15^,^16^, the *L. donovani* ribosome features several rRNA expansion segments located along the periphery of the structure (coloured in Fig. 3). These expansions are less pronounced in the LSU (Fig. 3a) compared with the SSU (Fig. 3b) in which a large ES6S expansion is seen along the body of the subunit. The entire ribosome appears in a conformation resembling an intersubunit-rotated state, with a discernible E-site transfer RNA (tRNA) (Fig. 3c,d). In most ribosomes the LSU rRNA is known to exist as a single 28S rRNA molecule, but in ribosomes from *Leishmania* and related trypanosomes the LSU rRNA is split into six segments (LSU-α, LSU-β, srRNA1, srRNA2, srRNA3 and srRNA4), likely produced by cleavage of a larger precursor^8^. These unique split rRNA segments are clearly resolved in our structure (see below). The leishmanial ribosome contains a conserved set of proteins (Fig. 2) as in other 80S ribosomes^4^,^7^,^14^,^15^, but the cryoEM map shows many trypanosome-unique protein extensions (up to 20 aa) that interact with rRNA expansion segments (see below).

### Unique features of the leishmanial ribosome

The ribosome of *L. donovani* contains a fragmented 28S rRNA, which features many significant differences when compared with the human ribosome. These regions are concentrated along the periphery, with the core regions being highly conserved. Many of the most notable differences occurs in the LSU, where fragmented rRNAs of *L. donovani* are located.

**Figure 4:**
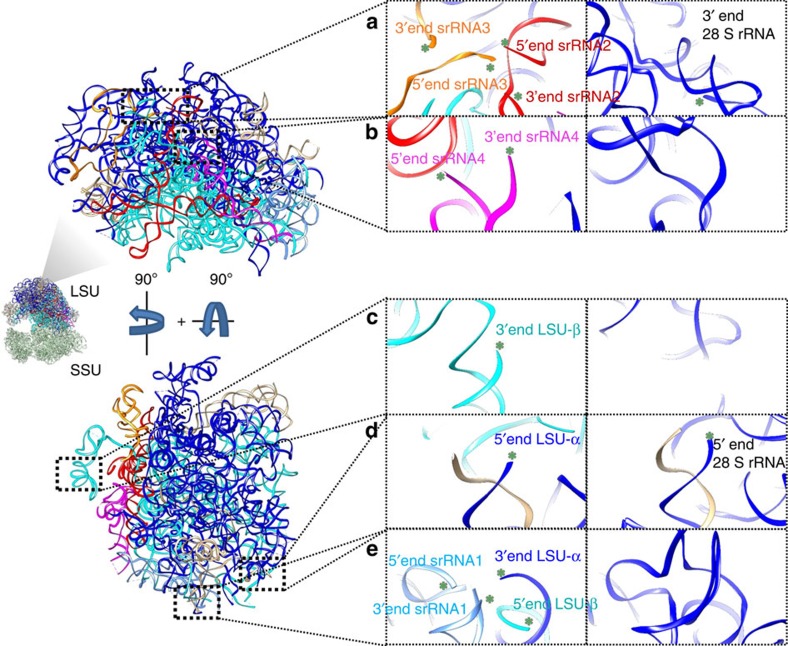
Unique rRNA end localization in the (left) *L. donovani* ribosome compared with homologous regions in the (right) human ribosome. Guide figures are rRNA components found in *L. donovani* and are related by two 90° turns, one along the *x* and one along the *y* axis. rRNA ends are denoted with green asterisks and labelled. The two 28S rRNA ends are labelled similarly for the human ribosome. For clarity, conserved 5S and 5.8S rRNA elements are shown in beige and are not labelled. Protein components are not shown. (**a**) Unique 3′ and 5′ ends of srRNA2 and srRNA3 compared with the homologous region in the human ribosome. (**b**) Unique 3′ and 5′ ends of srRNA4 compared with the homologous region in the human ribosome. (**c**) Unique 3′ end of LSU-β compared with the homologous region in the human ribosome. (**d**) The conserved 5′ end of LSU-α compared with the homologous region in the human ribosome. (**e**) Unique 5′ and 3′ ends of srRNA1, unique 3′ end of LSU- α and unique 5′ end of LSU-β compared with the homologous region in the human ribosome. Corresponding density and full models are shown in Supplementary Fig. 7.

**Figure 5:**
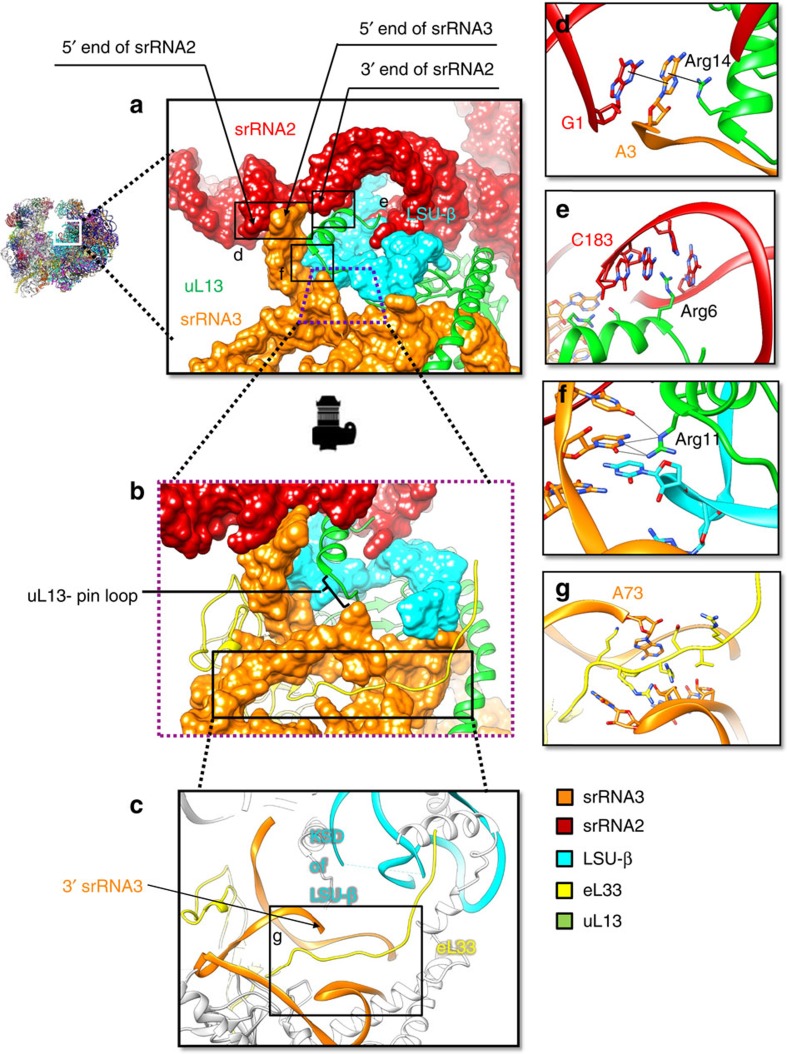
uL13-pin and rRNA fragmented end stabilization. (**a**) Surface representation of the region surrounding uL13-pin (ribbon), which is the trypanosome-unique N-terminal extension of uL13, showing the locations of rRNA ends in relation to uL13-pin. KSD and eL14 are not shown for clarity. (**b**) Rotated view of the trapezoidal region in (**a**) showing the rRNA ring (surface) formed by LSU-β (cyan) and srRNA3 (orange) through which uL13-pin passes through. (**c**) Arrangement of the trypanosome-unique extension of eL33 which binds the 3′ end of srRNA3 (orange) adjacent to uL13. (**d**) Sandwiching of the 5′ end of srRNA3 (A3) between the 5′ end of srRNA2 (G1) and Arg14 of uL13. (**e**) Binding of the 3′ end of srRNA2 (C183) by Arg6 of uL13. (**f**) Interactions between srRNA3 and Arg11 of uL13. (**g**) Binding of the 3′ end of srRNA3 (A73) by eL33. Full models with density maps available in Supplementary Figs 10 and 11.

The six split rRNA molecules in the LSU possess a total of 12 rRNA ends, as opposed to two for the single 28S rRNA molecule in the LSU of the human ribosome (Fig. 4). Our structure reveals that three of these rRNA ends are not involved in helical base pairing, which is energetically unfavourable in the absence of other stabilizing interactions. This problem is resolved by two new trypanosome-unique extensions in proteins uL13 and eL33 that bind these three cleaved rRNA ends and provide extra stabilization to a fourth rRNA end. The 5′ and 3′ ends of srRNA2 are bound by the extension of uL13, while the 5′ and 3′ ends of srRNA3 are bound by extensions of uL13 and eL33 respectively (Fig. 5a, Supplementary Figs 2a, 4a,b,d). Stabilization of the end with protein is also seen in the 18S SSU rRNA. The remaining six trypanosome-specific ends are stabilized by base pairing (Fig. 4; Supplementary Fig. 7), as in the ES20L/ES26L cleavage site^3^, while the two canonical 28S LSU RNA ends are stabilized by double helical basepairing (Supplementary Fig. 8b,c).

The ∼20 amino acid N-terminal extension of LSU protein uL13, unique to trypanosomes and previously unmodeled in the 5.57 Å *T. brucei* structure^3^, consists of a 9 a.a. loop and a three-turn helix (Fig 5a,b, Supplementary Video 6). We hereby name this extension as uL13-pin. uL13-pin is rich in basic amino acids and *pins down* three RNA molecules—srRNA2, srRNA3 and LSU-β –through electrostatic, hydrogen-bond and π-stacking interactions (Fig. 5d–f). These three rRNA molecules come together to form a three-sided pocket to accommodate uL13-pin (Supplementary Fig. 8a). Notably, uL13-pin binds the 5′ end of srRNA3 and 5′ end of srRNA2 on one side of the pocket. uL13-pin is situated in space occupied by H98 in the human and yeast 80S ribosomes and by LSU- β in *T. brucei* (Supplementary Fig. 9), necessitating RNA cleavage to accommodate the trypanosome-extension. In *L. donovani*, the loop of uL13-pin first passes through a narrow ring of rRNA formed by the base of the Kinetoplastid-specific domain (KSD) of LSU-β and srRNA3 (Fig. 5b, Supplementary Fig. 10) before the N-terminal helix interacts with the three-sided pocket formed by LSU-β, srRNA2 and srRNA3. This rRNA ring is also present in *T. brucei* but is absent in yeast and human 80S ribosomes, and is formed by RNA–RNA and RNA–protein interactions, sealing the ring in two places (Supplementary Fig. 10). Moreover, the helix of uL13-pin interacts with three rRNA cleaved ends, the 5′ and 3′ sites of srRNA2 and the 5′ site of srRNA3, and is in the immediate vicinity of two other rRNA ends. Arg14 of uL13-pin and G1 of srRNA2 sandwich A3 of srRNA3 (Fig. 5d, Supplementary Fig. 11b) through stacking interactions that mimic the ring stacking found in double helices, anchoring the 5′ ends of these molecules (srRNA2, srRNA3). On another side of the binding pocket, Arg6 of uL13-pin binds the 3′ end of srRNA2 (Fig. 5e, Supplementary Fig. 11a). In this way uL13-pin may mediate interactions between srRNA2, srRNA3 and LSU-β and serve as an anchor for these rRNA molecules. A variety of electrostatic and stacking interactions occur between srRNA3 and LSU-β in the region adjacent to uL13-pin (Supplementary Figs 10–11).

We expect uL13 and its trypanosome-unique N-terminal extension to play a significant role in the assembly of the leishmanial ribosome, given its proximity to and interaction with several ends of the cleaved rRNA. Indeed, uL13 is known to be required for formation of an early folding intermediate of the large subunit^17^. The presence of uL13-pin could potentially alter the folding pathway for the biogenesis of trypanosomal ribosomes as it occupies the space attributed to 28S rRNA in other eukaryotes. Indeed, the biogenesis of ribosomes in these organisms has been shown to involve novel preribosomal complexes as well as novel snoRNAs^9^,^18^,^19^.

Another area involving trypanosomal-unique protein–RNA interaction concerns the arrangement of srRNA3 (Fig. 5a,b). It differs from that in the earlier low-resolution structure of *T. brucei* ribosome^3^, occupying the space previously attributed incorrectly to srRNA2 (nucleotides 1–4). In particular the 5′ and 3′ ends of srRNA3 are positioned differently. A previously unmodeled trypanosome-specific extension of eL33 interacts with the backbone of srRNA3 at the base of the KSD (Fig. 5c,g, Supplementary Fig. 4e). We propose that this extension may also help secure the 3′ end of srRNA3. Moreover, the trypanosome-unique uL13-pin is adjacent to the eL33 extension. The presence of these unique protein extensions amidst multiple cleaved rRNA ends and the KSD suggests that the region around uL13-pin holds structural and developmental significance in trypanosomes.

### Unique structures with putative functional significance

**Figure 6:**
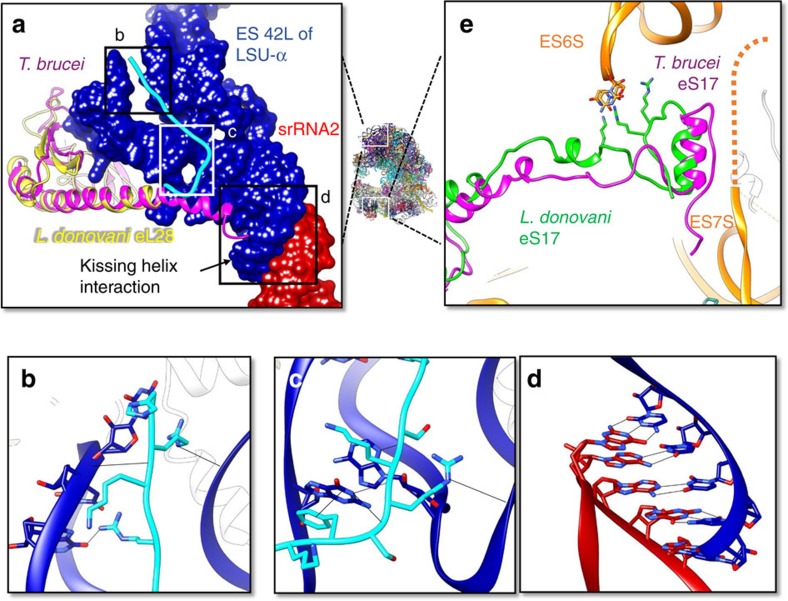
Functionally significant unique structural features in the *L. donovani* ribosome. (**a**) The unusually large ES42L (blue) binds srRNA2 (red) and a C-terminal extension of eL28 (yellow and cyan) in *L. donovani*. eL28 rests in a groove which lies between two RNA helices in ES42L. The *L. donovani* unique portion of the extension is coloured cyan, superimposed against that in *T. brucei*^3^ (magenta). (**b**,**c**) Boxed regions of (**a**) showing interactions between the C-terminal extension of eL28 and ES42L. (**d**) A closeup, 180° rotated view of the boxed region in (**a**), showing the kissing helix interaction between srRNA2 and ES42L formed by canonical basepairing. (**e**) Positioning of eS17 in *L. donovani* and *T. brucei* in relation to the trypanosome unique portions of ES6S and ES7S. Dashed line depicts flexible region of ES7S visible in the cryoEM map.

Of the many unique structural features of the leishmanial ribosome, two regions with putative functional significance in trypanosomes are related to ES6S/ES7S and the kissing helix interaction between srRNA2 and LSU-α identified in the low-resolution structure of the *T. brucei* ribosome^3^. The proximity of ES6S/ES7S to the messenger RNA (mRNA) channel points to a possible role in a unique translation process in trypanosomes, while the kissing helix interaction is thought to stabilize the srRNA column that mimics domain VI of 28S rRNA (ref. 3). Our structure shows that *L. donovani* proteins localized to these regions exhibit significant structural uniqueness as compared with non-trypanosomal ribosomes, and surprisingly even to *T. brucei*. In the structure formed by srRNA2 and LSU-α, ES42L of LSU-α is unusually large and meets srRNA2 to form a ‘kissing–helix' interaction through canonical base pairing (Fig. 6a–d). Nearby, protein eL28 fashions a trypanosome-unique C-terminal extension, which is rich in basic residues and rests in a groove in ES42L, stabilizing the structure (Fig. 6b,c). This extension is also present in the 5.57 Å resolution structure of the *T. brucei* ribosome^3^, though it adopts a different conformation and its C-terminal end is ∼48 Å from that in *L. donovani* (Fig. 6a). We suggest that the kissing–helix interaction stabilizes ES42L in conjunction with the *Leishmania* unique protein extension of eL28.

Translation in trypanosomes is thought to utilize unique mechanisms and protein factors^20^. Moreover, all trypanosomal mRNA have a unique 5′ spliced leader and an unusual cap 4 structure both of which likely play a role in translation^21^,^22^. Among the many expansion segments in the *L. donovani* ribosome, ES6S and ES7S are the most significantly enlarged (pink and green colour in Fig. 3b) as compared with other eukaryotic ribosomes^2^,^3^,^4^,^5^,^7^,^14^,^15^. Their proximity to the mRNA exit channel (Fig. 3b) suggests a possible role in translation initiation^3^. In particular, ES6S has been implicated in the recruitment of eukaryotic initiation factor 3 (eIF3)^7^. Some of the bases of ES6S and ES7S are not as well resolved as bases elsewhere, indicating flexibility of these two elements, despite the fact that ES6S (and possibly ES7S) binds the C-terminal end of eS17, which is rich in charged residues (Fig. 6e).

### Insights into paromomycin specificity

**Figure 7:**
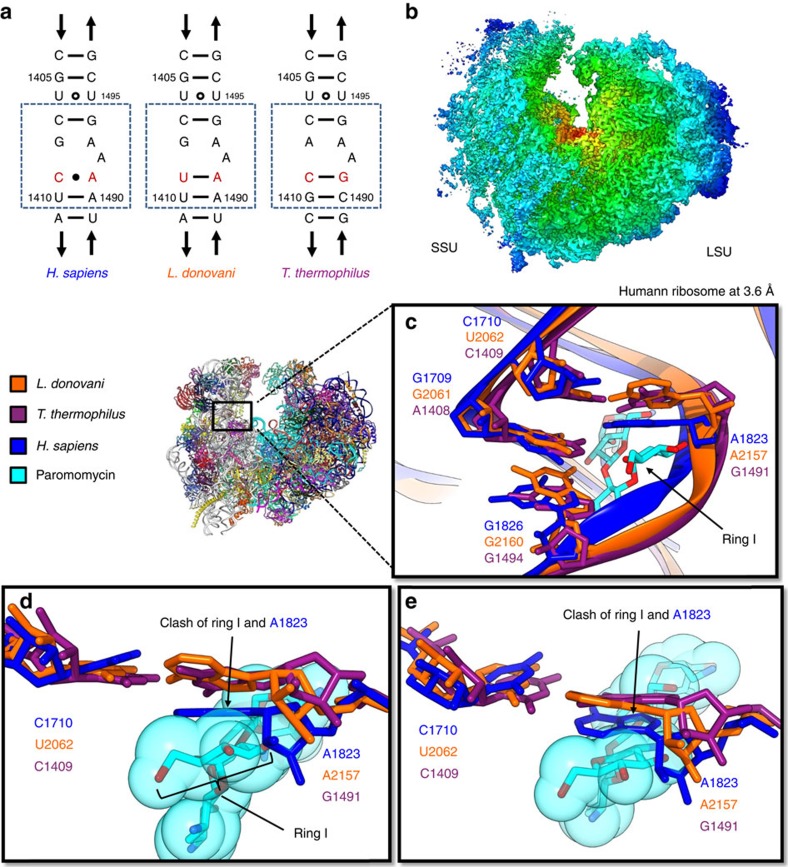
Human ribosome structure and insights into paromomycin specificity. (**a**) Decoding A-site sequences of *Homo sapiens*, *L. donovani*, and *Thermus thermophilus* ribosomes. The binding site of paromomycin is boxed in this sequence representation. Red text denotes the 1,409–1,491 basepair which is broken in *H. sapiens* and other eukaryotes but restored in *L. donovani.* (**b**) CryoEM reconstruction of the human 80S ribosome at 3.6 Å coloured by radius. (**c**) Superimposition of the decoding A-sites of the human and the *L. donovani* ribosomes with that of the *T. thermophilus* ribosome complexed with paromomycin^30^. Bases of 1,492 and 1,493 are hidden for clarity. (**d**) Comparison of the 1,409–1,491 basepair displayed in (**c**) in *H. sapiens*, *L. donovani* and *T. thermophilus* ribosomes, highlighting the unfavourable bonding angle between 1,409 and 1,491 (C1710 and A1823 human numbering) in the human ribosome (blue). Paromomycin displayed as a space-filling model to illustrate the severe clash with A1823 of the human decoding site. (**e**) A 20° related view of (**d**) along the *x*-axis. Black text indicates *E. coli* numbering, and coloured text corresponds to the species colour legend (orange, *L. donovani*; dark purple, *T. thermophilus*; blue, *H. sapiens*).

Paromomycin is a broad-spectrum ribosome-targeting antibiotic commonly used to treat prokaryotic infections. The cytoplasmic target of paromomycin is the decoding A-site of the SSU, where it increases misreading and causes translation inhibition^23^,^24^. Paromomycin does not bind most eukaryotic cytosolic ribosomes, which has been attributed to a steric hindrance caused by a 1408A to G mutation in the 18S rRNA helix h44 forming part of the A-site (Fig. 7a). Surprisingly, despite the decoding A-site of the leishmanial ribosome also containing 1408G, paromomycin binds the leishmanial ribosome and has in fact emerged as a promising new drug for treating visceral leishmaniasis due to drug resistance to traditional treatments. The large differential in its effects on translation in *Leishmania* versus most mammalian cells gives it a favourable safety profile for clinical use^23^. The sequences at the decoding A-sites of *Leishmania* (1404–1411 and 1489–1497, *Escherichia coli* numbering) and most other eukaryotes differ only by a single base pair (1409U to C) (Fig. 7a). A wealth of biochemical and structural data has established that mutations at positions 1408 and 1491 are critical for determining sensitivity^16^,^25^,^26^ to paromomycin and other aminoglycosides^16^,^23^,^27^,^28^,^29^; however the structural basis underlying this specificity is not well understood.

The paromomycin binding pocket within the decoding centre consists of ∼9 nucleotides in the major groove of a highly conserved SSU rRNA helix h44 (Fig. 7a). Direct structural comparison of the paromomycin binding sites between sensitive and resistant ribosomes is necessary to identify structural factors which affect its binding. To eliminate subtle structural variability in the binding pocket that could arise from sample preparation, imaging and computing processes, we have determined a 3D structure of the human ribosome with the same sample freezing method, instrument and data processing protocol to 3.6 Å resolution. Our human structure shows great similarity to that published by Khatter *et al*.^4^, with an r.m.s.d. of 0.6 Å between the structures.

For clarity of discussion, we decided to use one (that of the *E. coli* ribosome) sequence numbering to designate the corresponding nucleotides in all three species. The strong base pairing between C1409 (U2062 in *Leishmania donovani*) and G1491 (A2157 in *L. donovani*) nucleotides present in the leishmanial ribosome is attenuated in the human ribosome as judged by an unfavourable hydrogen-bonding angle and the electron density (Fig. 7b and Supplementary Fig. 12). Superimposition of the 18S rRNA structures of the human ribosome, the *L. donovani* ribosome and the *Thermus thermophilus* ribosome^30^ reveals that the core regions of the SSU match one another closely. However, nucleotide A1491 at the decoding site would clash with paromomycin in the human ribosome (Fig. 7d,e), thus providing a structural explanation for paromomycin specificity in addition to the various previously proposed mechanisms, including destabilization of ring stacking with paromomycin^31^, allosteric effects^28^ and clashing with hydroxyl groups^16^,^28^. This interpretation is supported by biochemical studies performed on the decoding site which indicate that the presence of non-canonical basepairing at positions 1,409–1,491 leads to reduced sensitivity to paromomycin^32^,^33^.

In summary, by taking advantage of the recent technology breakthrough in cryoEM, we obtained the ribosome structure of the *Leishmania donovani*, a medically important pathogen. The structure reveals many rRNA and protein domains unique to the kinetoplastid ribosomes. In particular, the structure of the six fragments of its large subunit rRNA (as opposed to the single 28S rRNA of most eukaryotes, including humans) elucidates how the ends of the fragmented LSU rRNA are stabilized by RNA–RNA and protein–RNA interactions. Such fragmented rRNA holds significance to the biogenesis and diversity of the highly conserved ribosomes. Structural comparison at the decoding A-sites among our leishmanial and human ribosome structures and that of *T. thermophilus*^30^ provide insights into susceptibility to paromomycin.

## Methods

### Isolation of cytosolic ribosomes from *Leishmania donovani*

Cells of *L. donovani*, strain 1S LdBob (MHOM/SD/62/1S-CI2D) obtained from S. Beverley at Washington University in St Louis, were authenticated by sequencing of minicircles and maxicircles of kinetoplast-mitochondrial DNA (ref. 34). The cells were propagated in 3L of the M199 medium with 10% FBS at 26 °C in Hepes buffer (pH 6.9) (ref. 35). Cells were pelleted and washed twice with SKS buffer (0.25 M sucrose, 5 mM KCl, 20 mM HEPES-KOH, pH 7.6) in a Beckman JA10 rotor at 6,000 r.p.m. for 20 min. Washed cells were suspended in SKS and disrupted using Stansted homogenizer (model A09512/716) operated at 80–100 p.s.i.; several passages of the suspension through the disruptor were required to achieve a near complete rupture. Unbroken cells and the cellular debris were removed by centrifugation in a JA14 rotor at 12,000 r.p.m. for 20 min. The S30 fraction was then obtained by centrifugation in a Beckman 60Ti rotor at 17,500 r.p.m. for 30 min. A crude ribosomal pellet was then obtained from the S30 fraction by centrifugation in a 60Ti rotor at 26,500 r.p.m. for 22.5 h. The ribosomal pellets were resuspended in a minimal volume of the RS buffer (100 mM KCl, 10 mM Mg Cl2, 3 mM DTT, 0.1 mM EDTA, 50 mM HEPES-KOH, pH 7.6). The aggregated material was removed from the suspension by pelleting 14,000 r.p.m. for 10 min in a benchtop microcentrifuge. The ribosomes present in the supernatant material were then sedimented through a 7–30% sucrose gradient (made with the RS buffer) in a Beckman SW28 rotor at 17,500 r.p.m. for 16 h. Each gradient was fractionated into 20 fractions using a Bio-Comp gradient station (model 153). A260 values in each fraction were measured using NanoDrop 2,000 spectrophotometer. Under these conditions most of the ultraviolet-adsorbing material was found in the bottom third of the gradient (fractions 13–20). This material was pooled, recovered by sedimentation in a Ti60 rotor (26,500 r.p.m., 22 h), resuspended in a minimal volume of the RS buffer and sedimented through 7–30% sucrose gradients (SW20 rotor, 16,000 r.p.m., 16 h). After measuring the A260 the ribosomal fractions were pooled, concentrated using Amicon Ultra-15 centrifugal filter device (Millipore), and dialysed overnight against 0.5 L of the RS buffer. The final preparation with RNA concentration of 0.86 mg ml^−1^ was used for microscopy.

### Isolation of cytosolic ribosomes from *H. Sapiens*

Human ribosomes were isolated from Jurkat cells, obtained from ATCC, grown in R10 medium (RPMI with 10% FBS and 1 × Pen/Strep/Glut). The cells were authenticated functionally by susceptibility to HIV infection and correct HLA, and were routinely tested negative for mycoplasma. A pellet of 0.4 g of cells was collected and lysed on ice for 10 min in 1 ml lysis buffer (1% Triton-x-100, 10 μl Protease Inhibitor (Sigma) and 4 μl 1 M DTT added to 1 ml ribosome prep buffer (40 mM HEPES pH 7.6, 100 mM KOAc, 20 mM Mg(OAc)2 with pH adjusted using KOH pellets)). Cell lysate was centrifuged at 20,000*g* at 4 °C for 20 min. The supernatant was further clarified by centrifugation at 20,000*g* at 4 °C for 20 min.

A stepwise (20, 30, 40, 45, 50 and 60%) sucrose gradient was created by dissolving sucrose (Fisher Biotech) in ribosome prep buffer with 4 mM DTT. We overlaid the supernatant containing human ribosomes on the top of the sucrose gradient and subsequently centrifuged with a SW41 rotor (Beckman Coulter) at 25 K at 4 °C for 16 h. The 40 and 45% sucrose fractions were collected, pooled together and dialysed with 1 MDa membrane tubing (Spectrum Laboratories) against the ribosome prep buffer with 3 mM DTT for 1 day at 4 °C. The dialysed sample was evaluated by negative stain EM and used for cryoEM.

### CryoEM imaging

A 2.5 μl aliquot of purified ribosome sample was applied to a Lacey holey grid with thin continuous carbon film for 1 min, blotted for 15 s with an FEI vitrobot in 100% humidity, and then plunged into liquid ethane. CryoEM images were recorded in an FEI Titan Krios cryo electron microscope, operated at 300 kV with a nominal magnification of × 75,000 or × 46,730 for the leishmanial and human ribosomes, respectively. The microscope was carefully aligned and electron-beam tilt was minimized by a coma-free alignment procedure. Image stacks (movies) were recorded on a Gatan K2 direct electron detection camera in its counting mode with a pixel size of either 0.8 or 1.07 Å per pixel on the specimen for the leishmanial and human ribosomes, respectively. The dose rate of the electron beam was set to ∼8e- per pixel per s, and the image stacks were recorded at 4 frames per s for 8 s. The drift between frames in each image stack was corrected with the UCSF drift correction software^36^, and frames in each stack were merged to generate a final image with an accumulated electron dose of ∼22 and ∼25e− Å^−2^, for the leishmanial and human ribosomes, respectively.

### Image processing and 3D reconstruction

We used DoGPicker^37^ to pick 244,170 and 260,692 particles automatically from 6,044 to 4,425 movies for the leishmanial and human ribosomes, respectively. The underdefocus values of these images were determined to be 1.1–2.5 μm and 1.0–3.6 μm for the leishmanial and human ribosomes, respectively, with CTFFIND (ref. 38). Image processing and reconstruction were carried out with RELION^39^. 2D and 3D classifications were used to screen particles. For the leishmanial ribosome, all particles were divided into 50 classes, and after 20 iterations of 2D classification, 17 good classes were selected for the following 3D classification (Supplementary Fig. 13). Three classes were used in the 3D classification, and starts from 60 Å resolution with a starting model generated by low-pass (cosine) filtering the cryoEM structure of the yeast 80S ribosome^40^ (EMD-2275) to 60 Å resolution. After 25 iterations, 2 of the 3 classes were basically identical, while the third class exhibited poor resolution and did not resemble a ribosome, suggesting partially broken or less-organized structures. Therefore, 213,108 particles screened from the 2D and 3D classifications were used for 3D refinement. The 3D refinement also starts from 60 Å resolution and uses the same initial model in the 3D classification.

The map of the whole ribosome of *L. donovani* was reconstructed from 213,108 particles selected from the original dataset of 244,170 particles. After 16 cycles of 3D refinement, the averaged resolution was estimated to be ∼2.9 Å based on the ‘gold-standard FSC' criterion with RELION and the estimation with ResMap (Supplementary Fig. 1c)^39^,^41^. This estimate is consistent with the base and sidechain features visible in the density maps (for example, Supplementary Figs 1–6, Supplementary Videos 1–5) and a calculated correlation coefficient of 0.5 at about ∼2.9 Å between the density map and atomic model calculated with PHENIX (Supplementary Table 1)^42^,^43^.

Because the resolution of the SSU in this map was worse than that of the LSU, we carried out a localized structure refinement to improve resolution of the SSU as follows. We first segmented out the SSU from the whole ribosome map. This segmented SSU map was low-pass-filtered to 15 Å and converted into a binary map. The [1-0] sharp edges in this binary map were ‘softened' by cosine function extending to six pixels with RELION to create a 3D soft mask. The whole ribosome map was then multiplied by this 3D soft mask to create an SSU map. This SSU map was then used to refine the orientation and centre parameters from the refinement result of the whole ribosome with RELION^39^. This localized 3D refinement was repeated with the latest masked SSU map for 10 times iteratively until the SSU map converged at a resolution of 3.39 Å, which is slightly better than that of the SSU in the whole ribosome map obtained above.

The human 80S ribosome images are processed following the same procedure, except that its SSU was not refined individually using the above masking protocol. The final map of the human was reconstructed from 175,708 particles selected from the original dataset of 260,692 particles after 2D and 3D screening. Of the five 3D classes, four were highly similar, while the fifth exhibited a much lower resolution, suggesting partially broken or less-organized structures. Thus, only particles from the four good classes were merged for 3D refinement. Based on the ‘gold-standard FSC' criterion with RELION and the estimation with ResMap, the resolution of the human ribosome structure converged to a resolution of 3.6 Å after 25 cycles of 3D refinement (Supplementary Fig. 1d).

The density maps of both leishmanial and human ribosomes were sharpened with a reverse B-factor of −80 Å^2^. Visualization and segmentation of density maps were done with UCSF Chimera^36^.

### Atomic modelling and visualization

Map interpretation was performed with UCSF Chimera^36^ and COOT^44^. Available homology models of the *Saccharomyces cerevisiae*^14^,^15^ and *Trypanosoma brucei*^3^ (6) ribosomes were utilized as initial references to assist with model building. *L. donovani* protein sequences were obtained from the National Center for Biotechnology Information (NCBI) protein databases^45^ (http://www.ncbi.nlm.nih.gov/). *Leishmania infantum*, *Leishmania braziliensis* and *Leishmania major* sequences were used for proteins where no *L. donovani* sequence was available or where the *L. donovani* sequence was incomplete.

Due to the presence of numerous flexible regions of rRNA, the proteins of the LSU and SSU of the Leishmania donovani ribosome were first modelled manually in COOT to ensure accurate tracing of the rRNA backbone. There are several regions where secondary structures were clearly resolved, however the local resolution of these regions is insufficient for accurate registration of specific amino acids and bases. eL31, eL40 and eL24 contain such regions and are thus not fully modelled. After the correct placement for each amino acid residue was ensured, the backbone was converted to a purely alanine backbone by the COOT function ‘Mainchain,' and mutated to the corresponding amino acids through the function ‘Mutate Residue Range.' With the initial model now completed, the ‘Density Fit Analysis' validation tool was used to screen for regions of the model that did not fit the density map. When identified, these sequences and the amino acids surrounding them were examined for any other possible conformations that better fit the density. Refinement was also performed based on the Ramachandran plot: any residues with disallowed values were selected, and the stereochemistry of that residue along with its surrounding residues was optimized with the refinement tool ‘Regularize Zone.' After reasonable Ramachandran values were obtained (<10% outliers), the refinement function ‘Rotamers' was used to select the rotamer, or low energy side chain conformation, that best fit the density. Additionally, PHENIX^42^ was used to improve fit of protein into the density through real-space refinement. Several expansion segments are less ordered, with secondary structures resolved but without sufficiently detail for modelling, as were some protein extensions.

For RNA modelling, homologous rRNA sequences were fetched from GenBank^46^ and aligned using Clustal Omega^47^ for analysis of expansion segments. Available homology models of the rRNA molecules from *S. cerevisiae*^14^,^15^ and *T. brucei*^3^ ribosomes were modified according to the RNA sequences of the *L. donovani* ribosome. Bases were manually adjusted with COOT using the ‘real-space refine' function, and all models were then refined with PHENIX^42^ as done previously^48^.

Modelling of the human ribosome followed the above procedures with COOT and PHENIX. Our cryoEM map is highly similar to the existing human ribosome structure in the pre-translocation state^4^, therefore, only the h44 structure at the decoding A-site was manually built from scratch in COOT and the existing models of the other human ribosome components in the pre-translocation state^4^ were fit into our cryoEM map to obtain crude models for both the large and small subunits. These LSU and SSU models were subsequently refined with PHENIX using an established procedure^48^.

All figures and movies were prepared with UCSF Chimera^36^ and Resmap^41^. Superimposition of ribosome structures was done with Matchmaker function in Chimera.

### Data availability

3D cryoEM density maps have been deposited in the Electron Microscopy Data Bank under the accession numbers EMD-8343 (leishmanial ribosome) and EMD-8345 (human ribosome). The coordinates of atomic models of the leishmanial and human ribosomes have been deposited in the Protein Data Bank under the accession number 5T2A and 5T2C, respectively. The data that support the findings of this study are available from the corresponding author on request.

## Additional information

**How to cite this article:** Zhang, X. *et al*. Structures and stabilization of kinetoplastid-specific split rRNAs revealed by comparing leishmanial and human ribosomes. *Nat. Commun.* **7,** 13223 doi: 10.1038/ncomms13223 (2016).

## Supplementary Material

Supplementary Information Supplementary Figures 1 - 13, Supplementary Tables 1 - 3 and Supplementary References

Supplementary Movie 1 Typical density (gray) and atomic model (backbone as ribbon and bases as sticks) of an rRNA strand in the *L. donovani* ribosome reconstruction.

Supplementary Movie 2 Typical density (gray) and atomic model (backbone as ribbon and sidechains as sticks) of an a-helix in the *L. donovani* ribosome reconstruction.

Supplementary Movie 3 Typical density (gray) and atomic model (backbone as ribbon and bases as sticks) of an rRNA double helix with canonical basepairing shown in the *L. donovani* ribosome reconstruction.

Supplementary Movie 4 Typical density (gray) and atomic model (backbone as ribbon and bases as sticks) of an rRNA double helix with canonical basepairing shown in the *L. donovani* ribosome reconstruction.

Supplementary Movie 5 Typical density (gray) and atomic model (backbone as ribbon and sidechains as sticks) of a protein loop in the *L. donovani* ribosome reconstruction.

Supplementary Movie 6 Zoomed-in view of the *L. donovani* ribosome homing in on extension uL-13 pin and its interactions with cleaved rRNA ends of srRNA2 and srRNA3. uL13 is colored green, srRNA2 red, srRNA3 orange, and LSU-β cyan.
