# Supplementary material for: Structures and stabilization of kinetoplastid-specific split rRNAs revealed by comparing leishmanial and human ribosomes
Source: Nat Commun. 2016 Oct 18;7:13223. doi: 10.1038/ncomms13223 (PMC5071889; doi:10.1038/ncomms13223)
Supplement: Supplementary Information — Supplementary Figures 1 - 13, Supplementary Tables 1 - 3 and Supplementary References [file ncomms13223-s1.pdf]

a

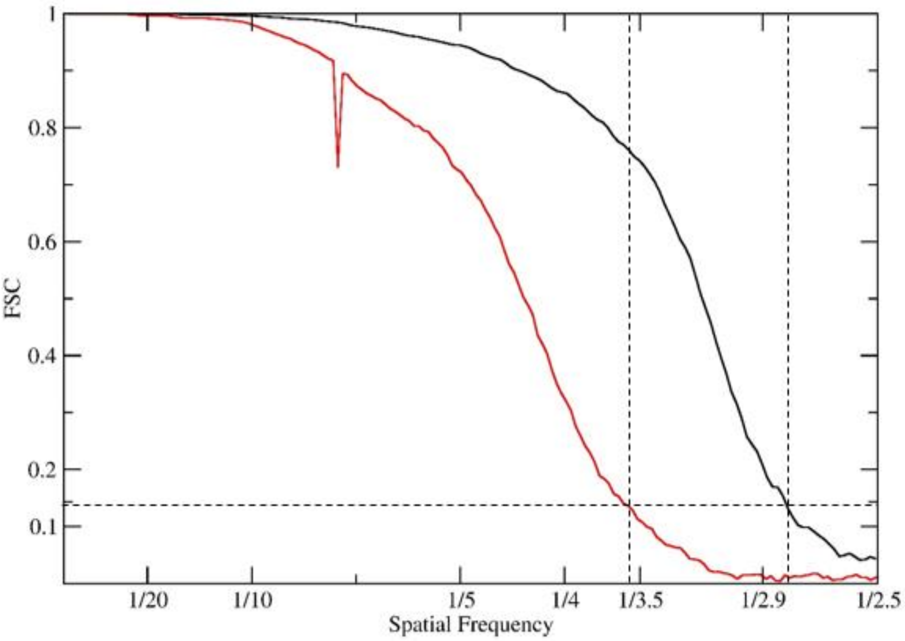
FSC
1
0.8
0.6
0.4
0.2
0.1
1/20
1/10
1/5
1/4
1/3.5
1/2.9
1/2.5
Spatial Frequency

b

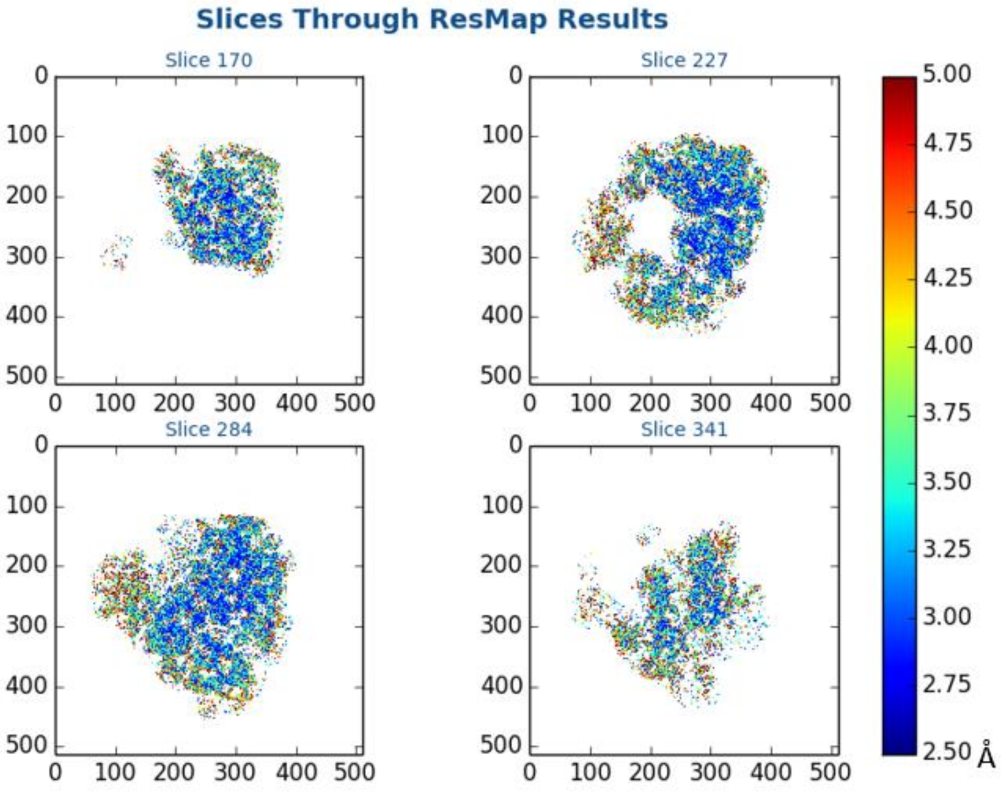
Slices Through ResMap Results
Slice 170
Slice 227
Slice 284
Slice 341
0
100
200
300
400
500
5.00
4.75
4.50
4.25
4.00
3.75
3.50
3.25
3.00
2.75
2.50 Å

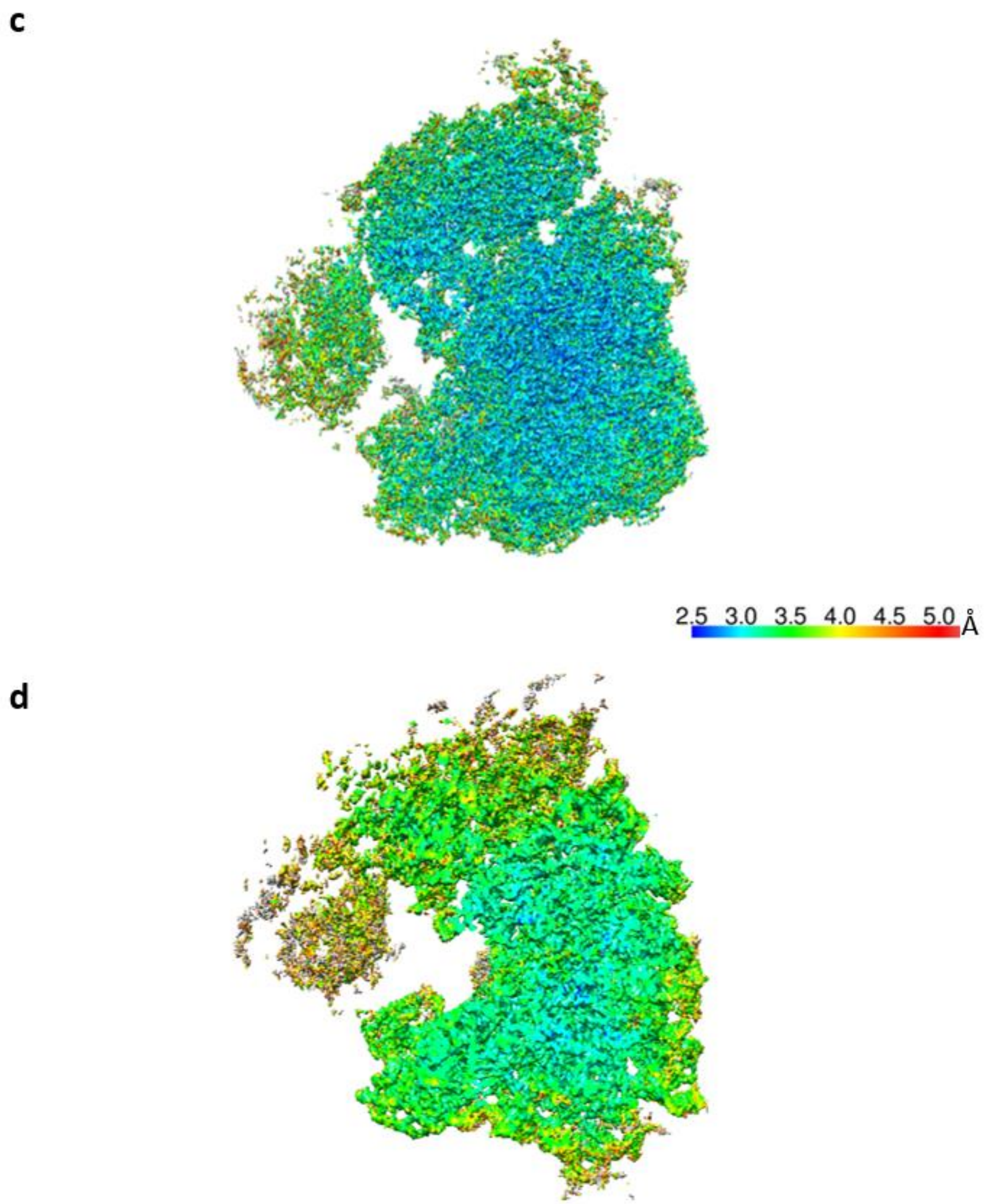

Supplementary Figure 1: Resolution assessment. (a) Gold standard Fourier shell cross-correlation coefficient of the leishmanial (black) and human ribosome (red) reconstructions by RELION[1]. (b) Local resolution assessment of the leishmanial ribosome reconstruction using Resmap[2]. (c) Colored surface representation of local resolution of the leishmanial ribosome (central section). (d) Colored surface representation of local resolution of the human ribosome (central section).

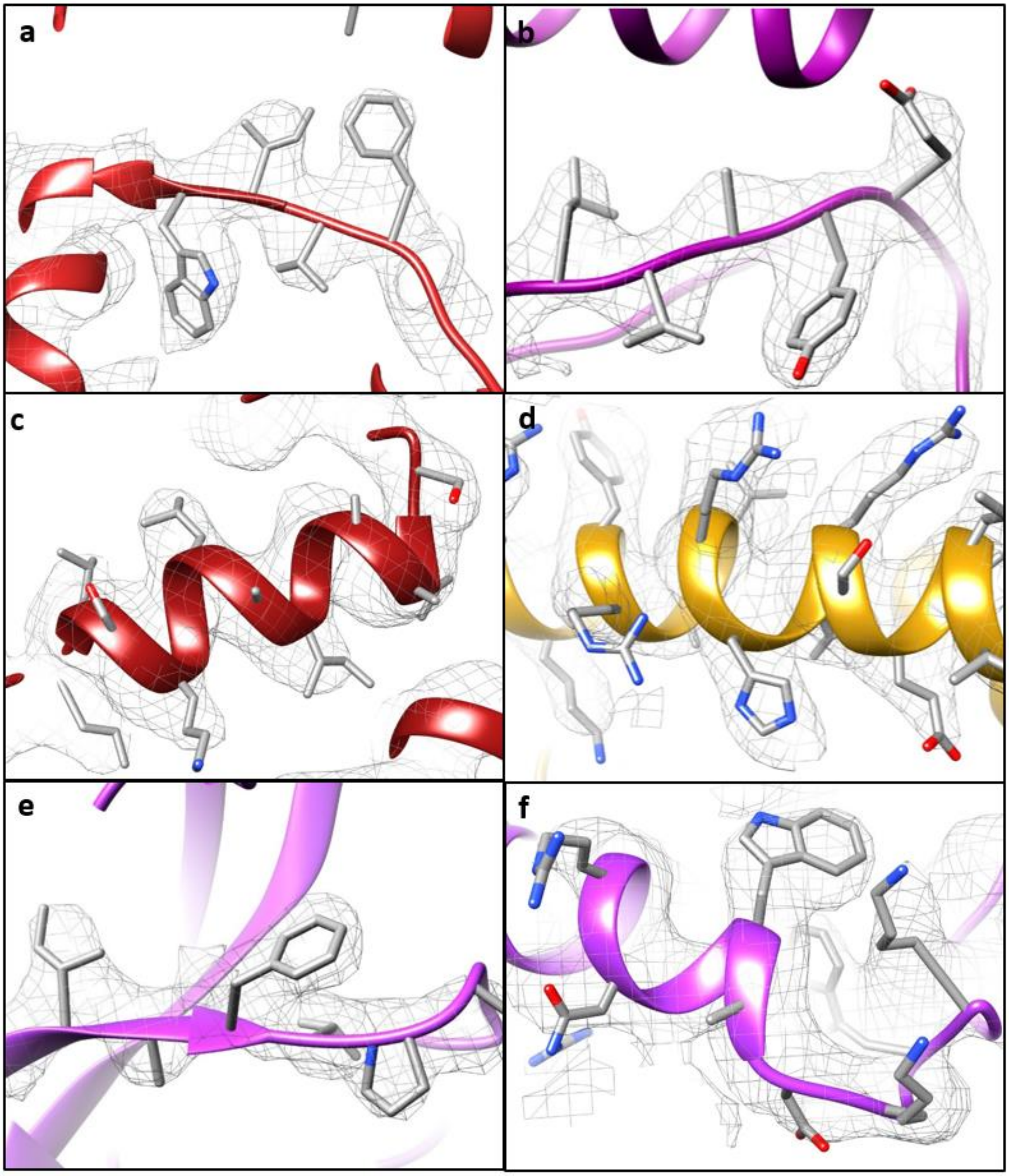

Supplementary Figure 2: Typical protein side chain densities in LSU (a-c) and SSU (d-f) of the *L. donovani* ribosome.

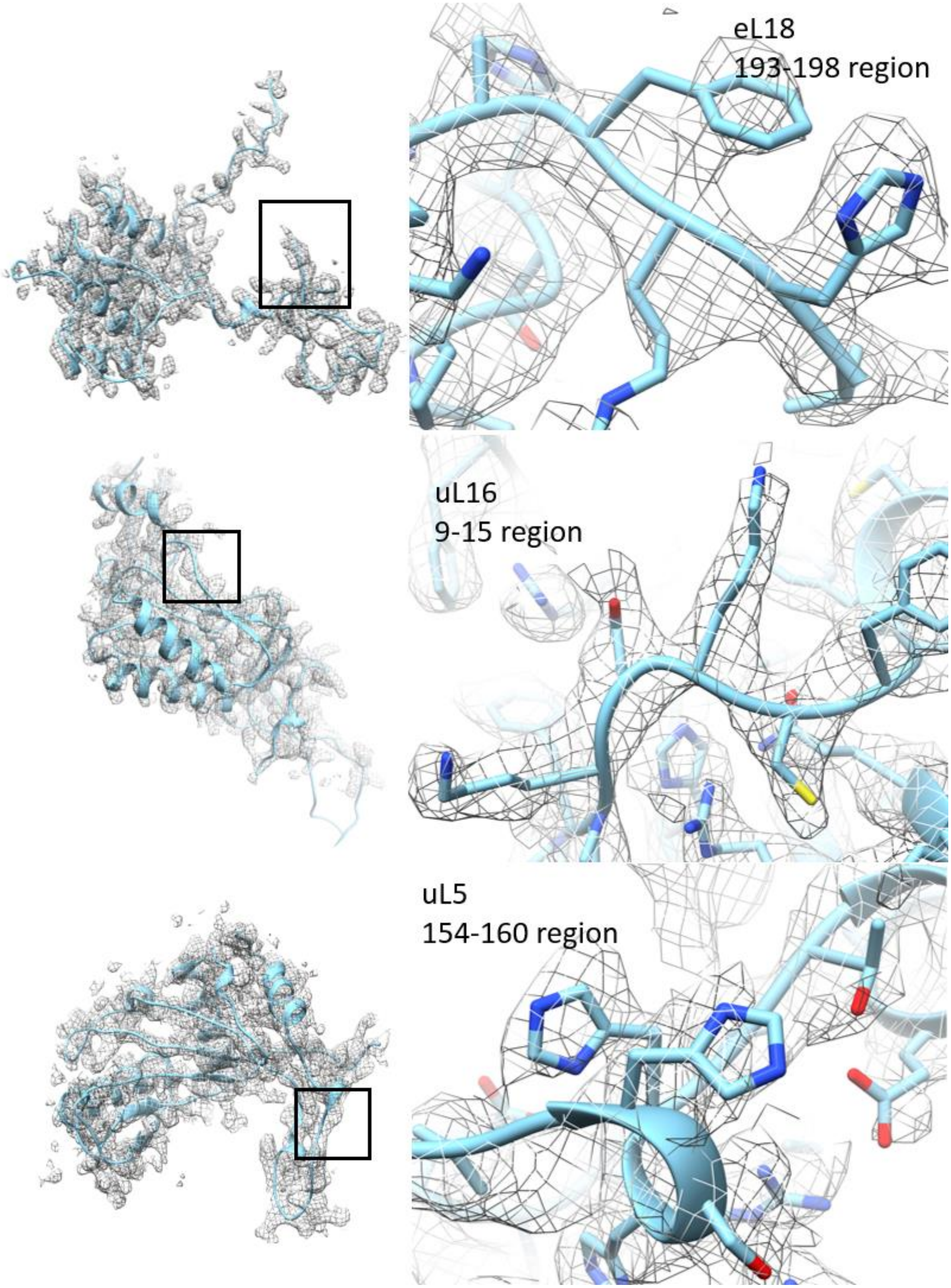
eL18
193-198 region
uL16
9-15 region
uL5
154-160 region

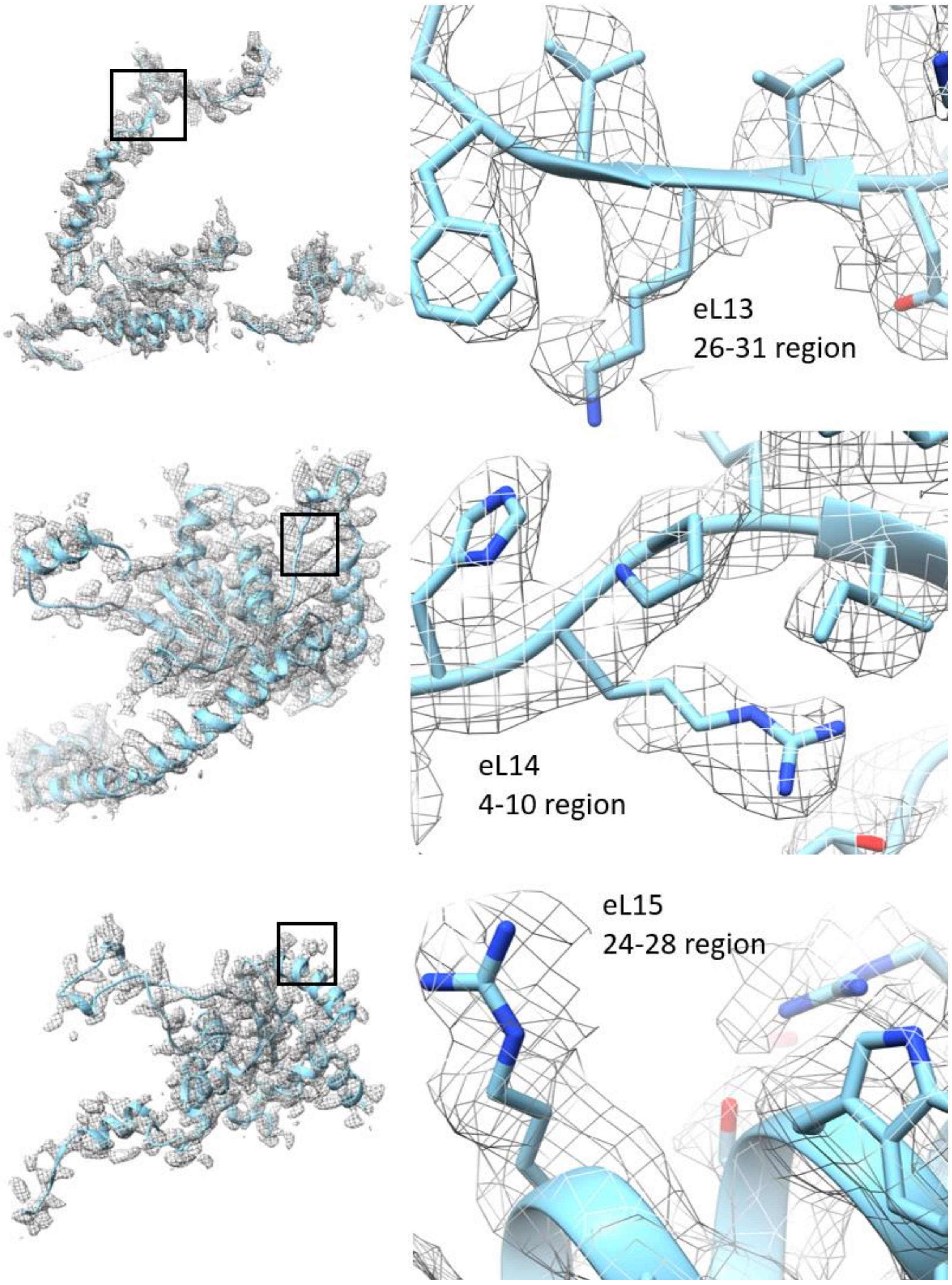
eL13
26-31 region
eL14
4-10 region
eL15
24-28 region

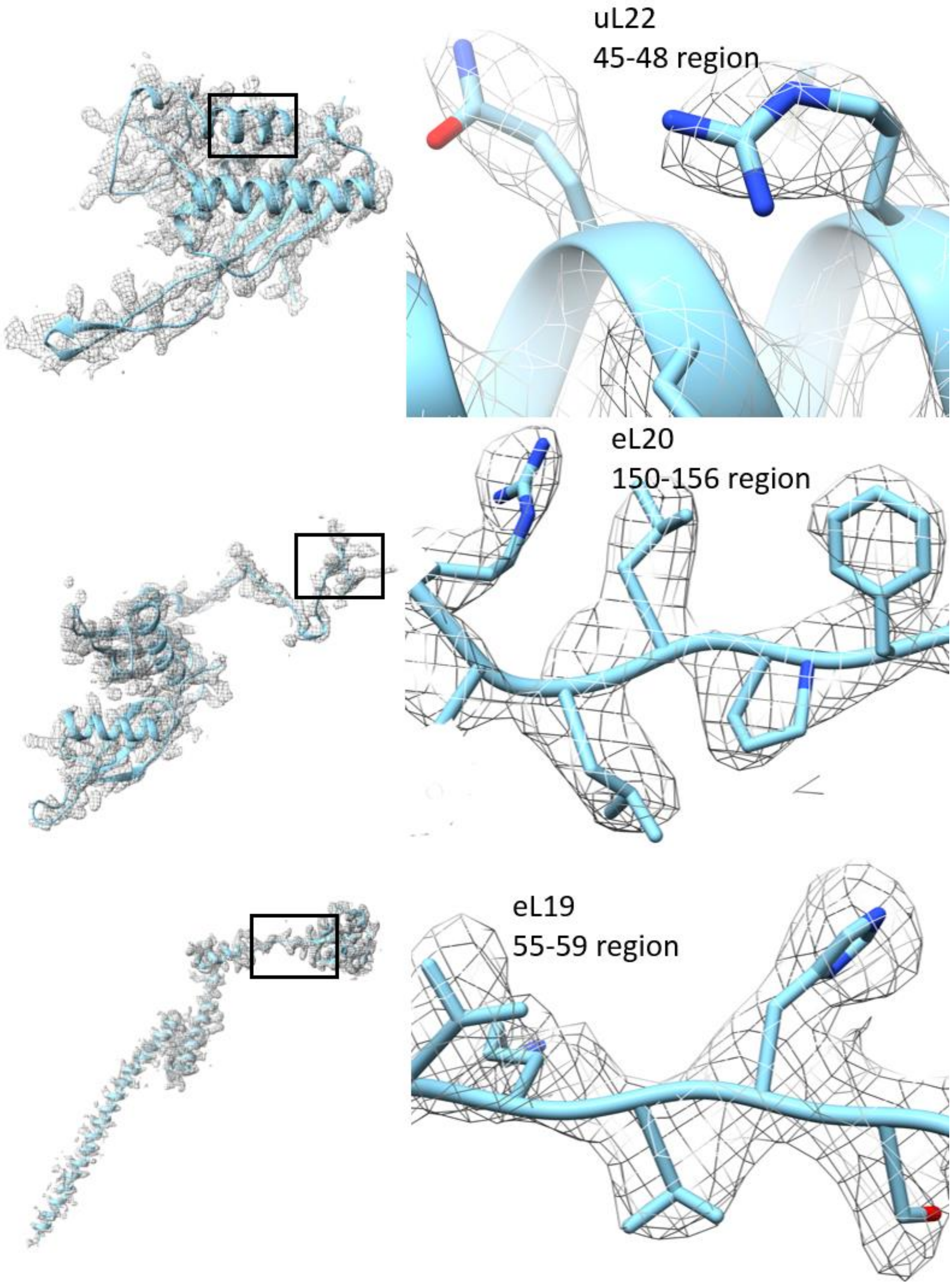

uL22
45-48 region
eL20
150-156 region
eL19
55-59 region

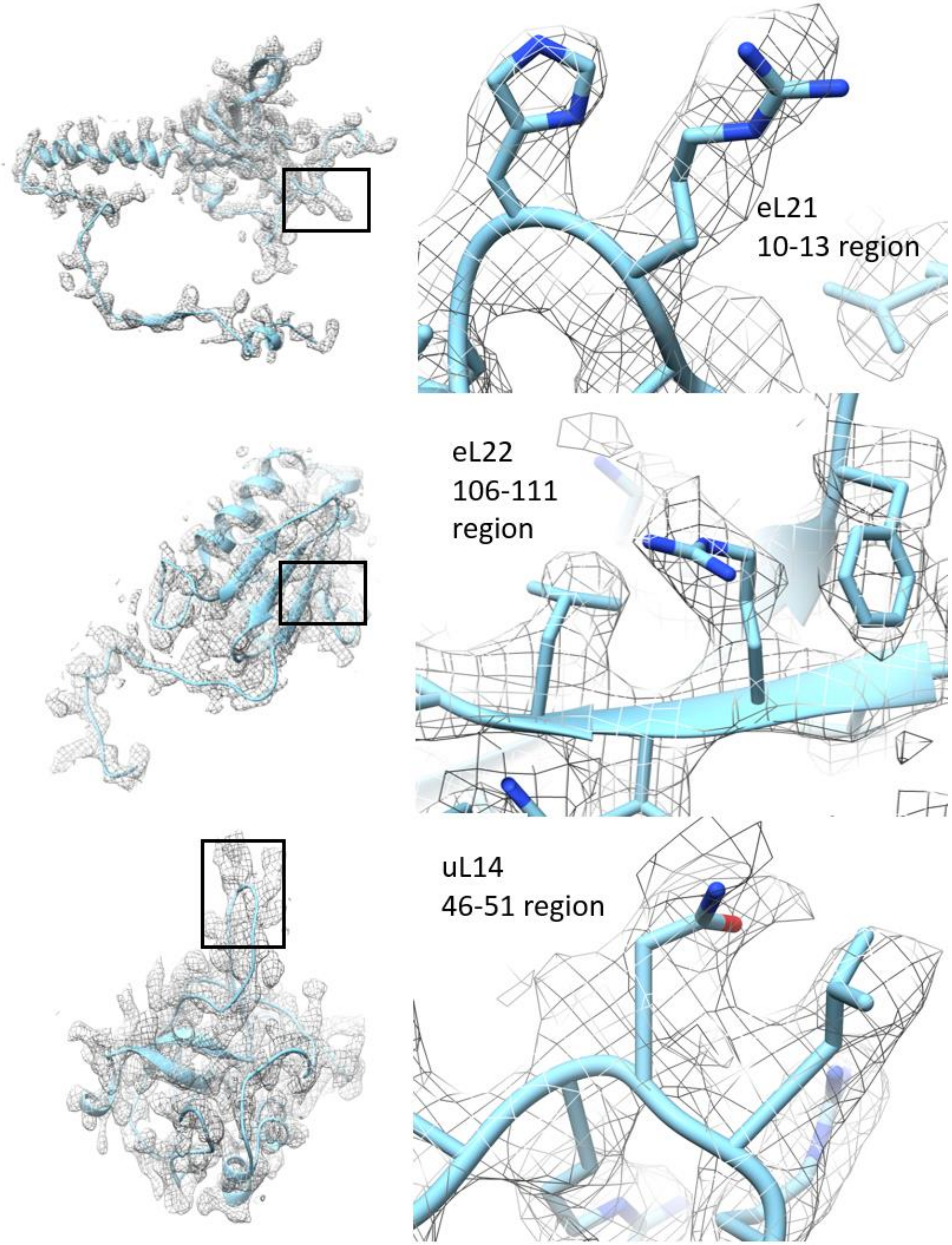
eL21
10-13 region
eL22
106-111
region
uL14
46-51 region

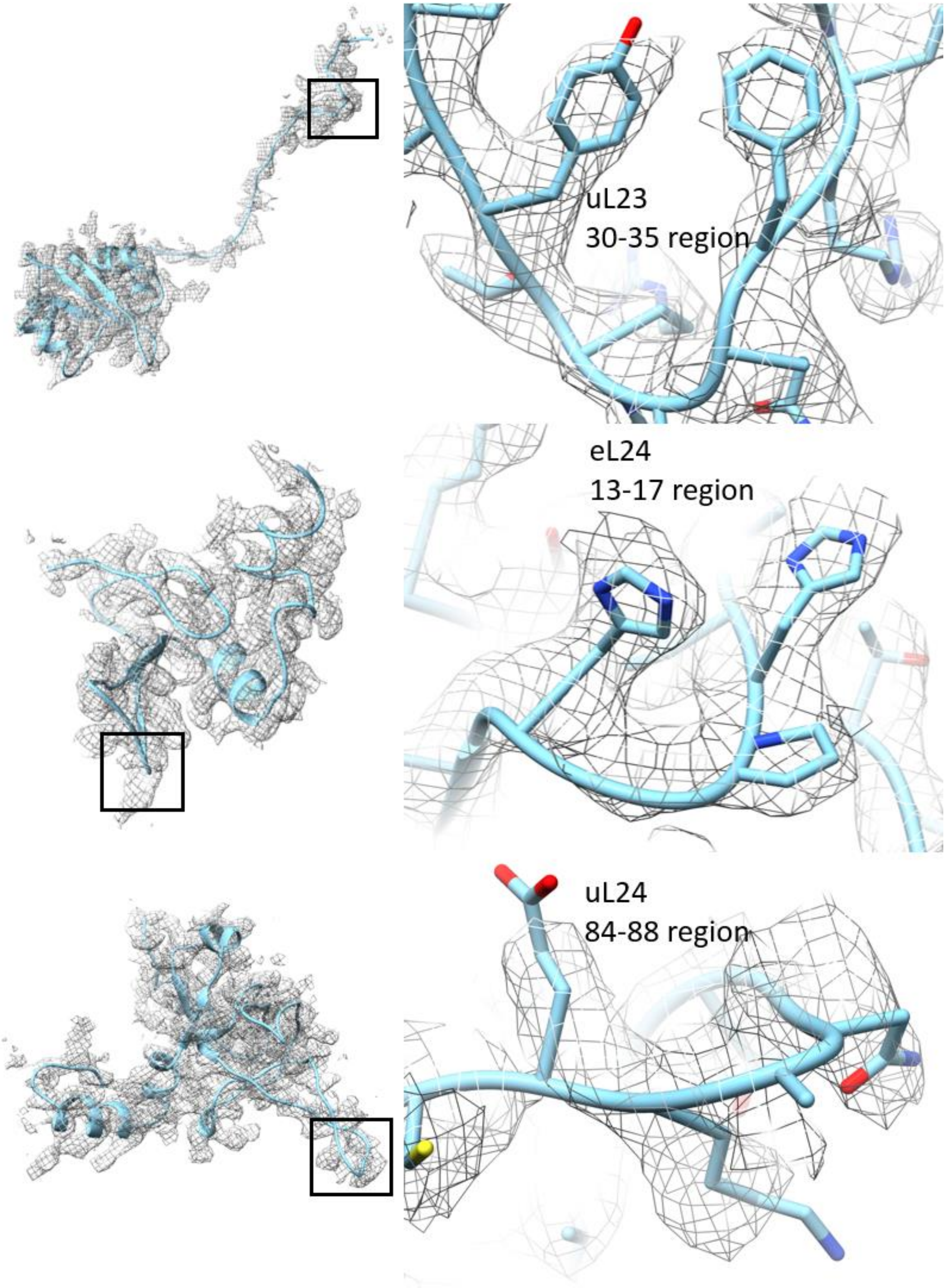
uL23
30-35 region
eL24
13-17 region
uL24
84-88 region

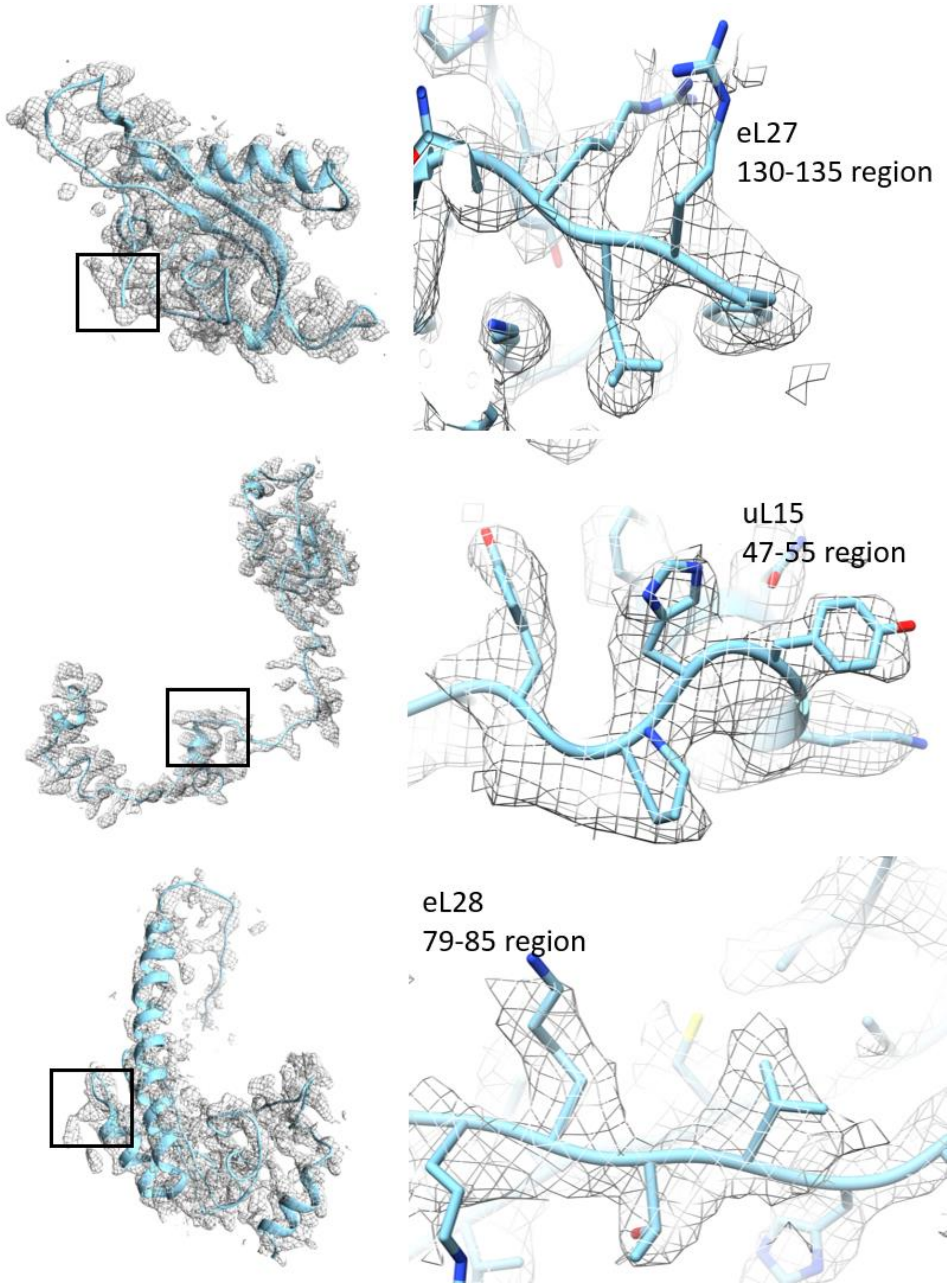
eL27
130-135 region
uL15
47-55 region
eL28
79-85 region

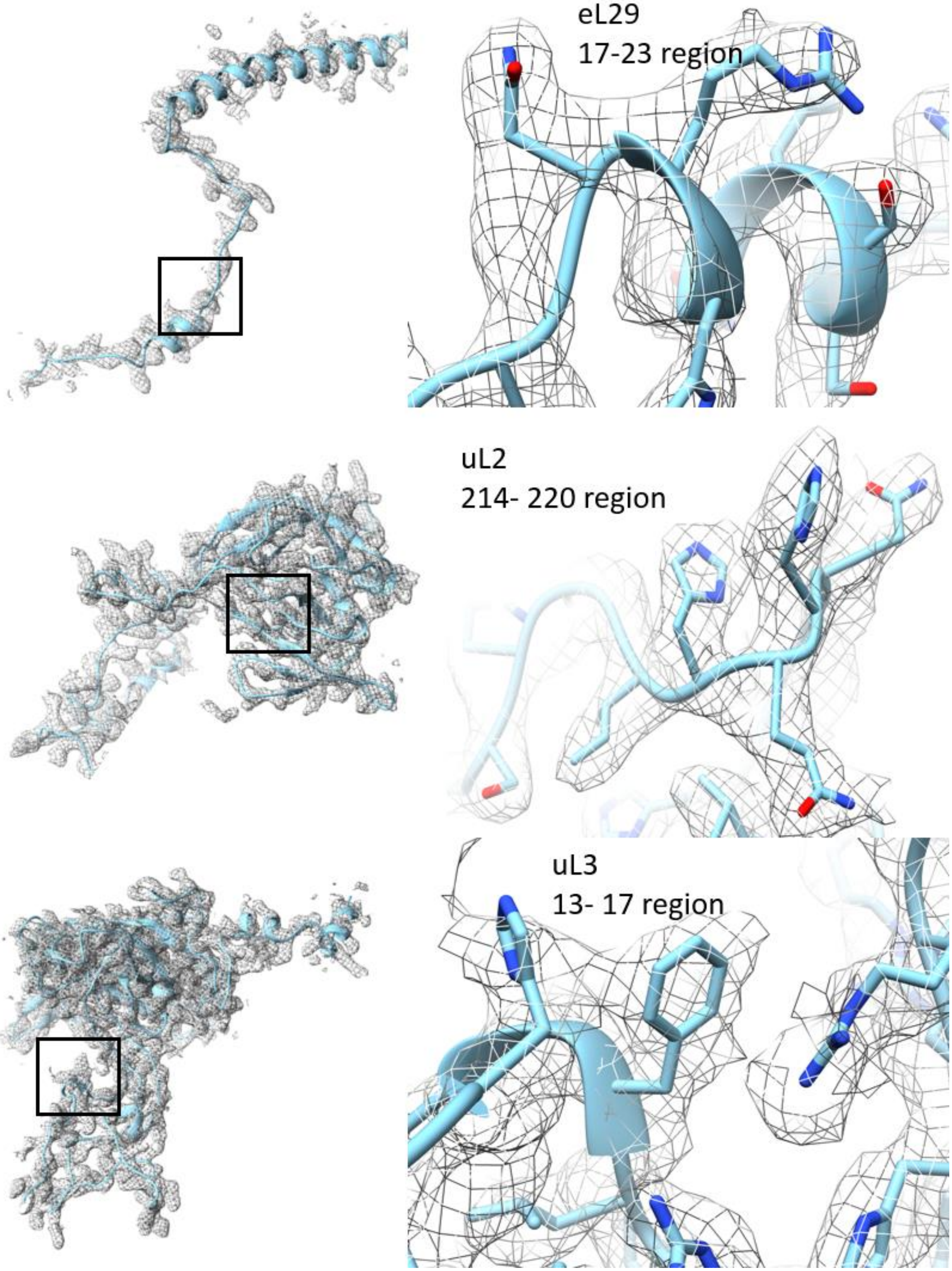
eL29
17-23 region
uL2
214- 220 region
uL3
13- 17 region

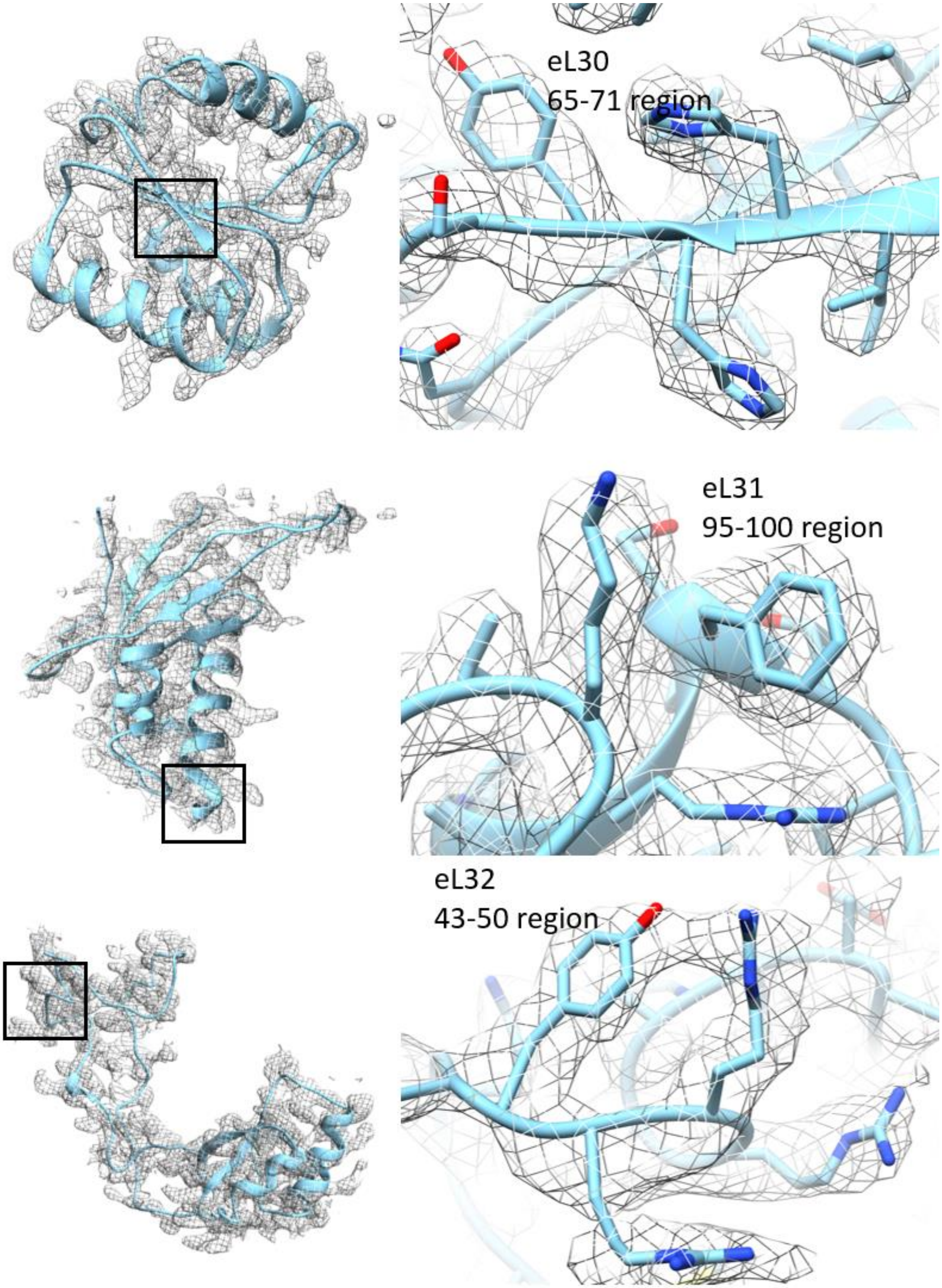
eL30
65-71 region
eL31
95-100 region
eL32
43-50 region

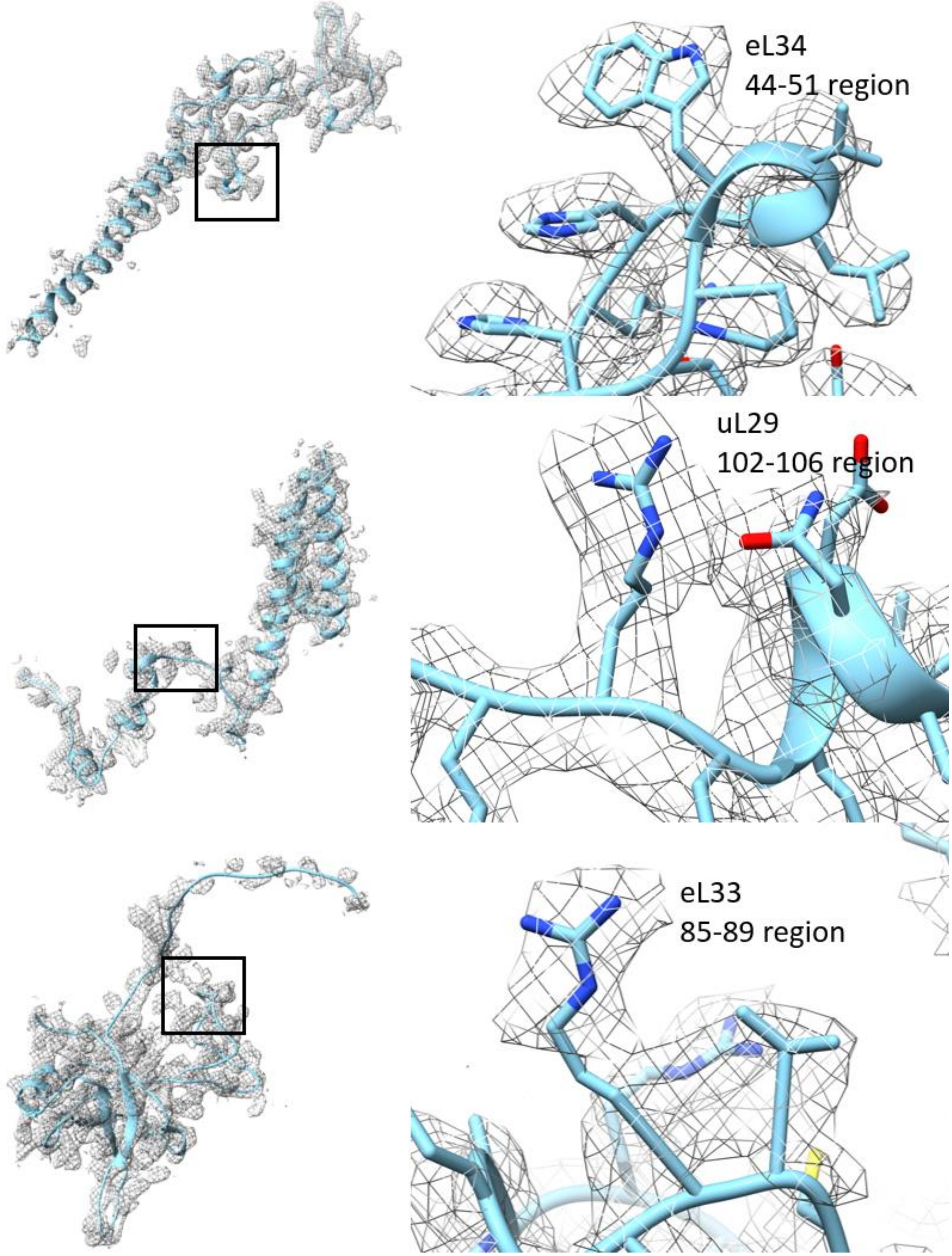
eL34
44-51 region
uL29
102-106 region
eL33
85-89 region

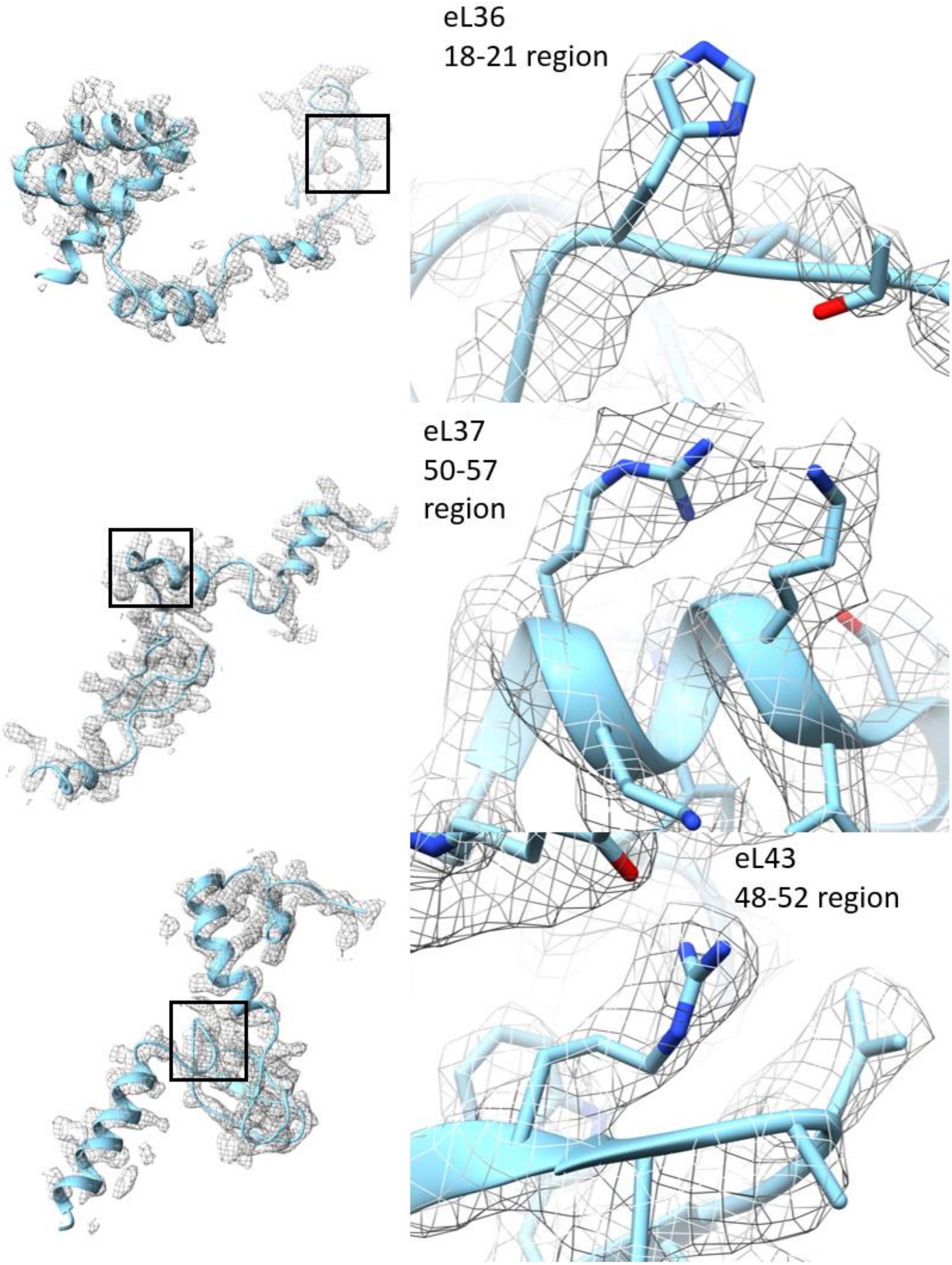
eL36
18-21 region
eL37
50-57
region
eL43
48-52 region

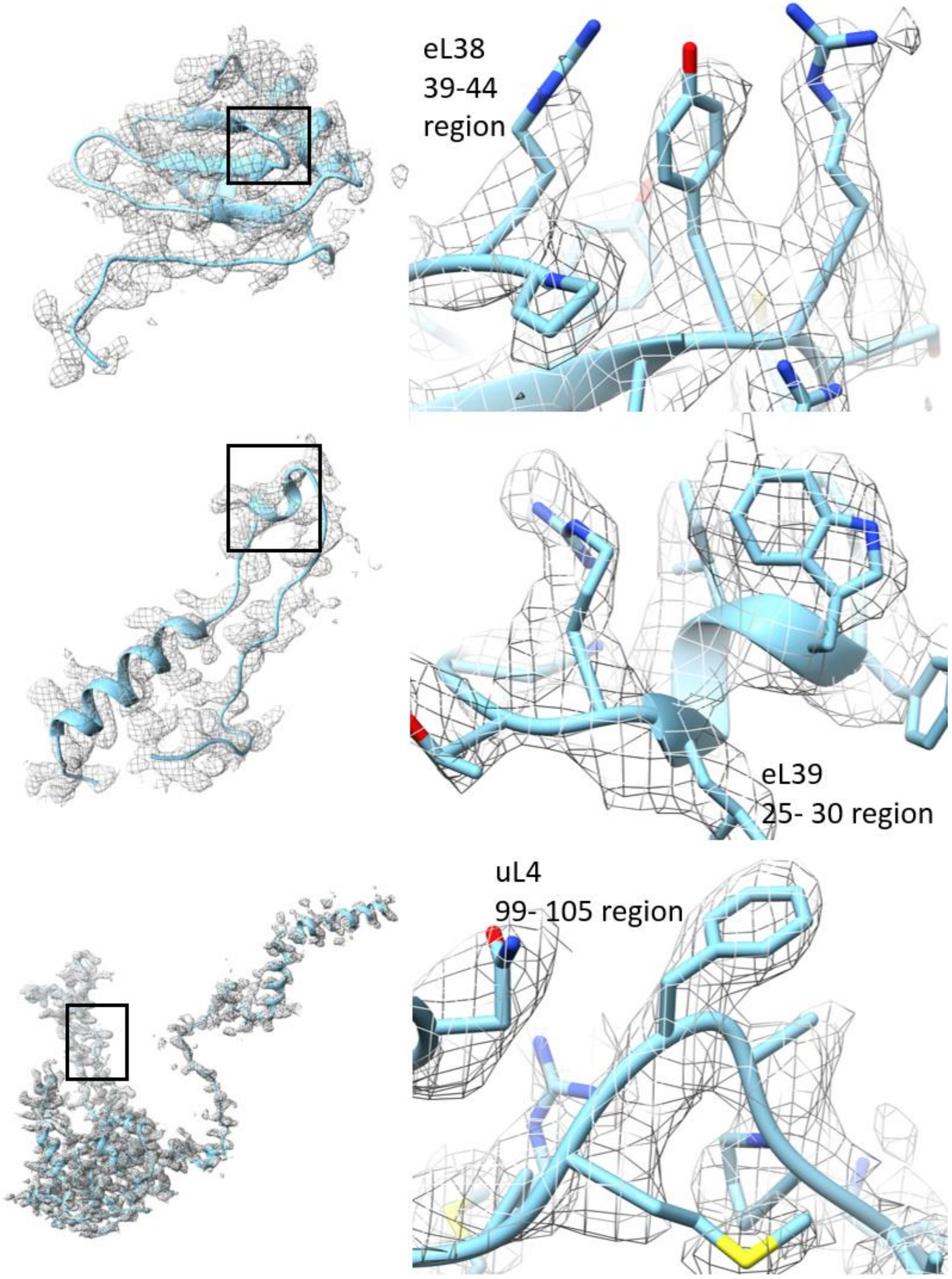
eL38
39-44
region
eL39
25- 30 region
uL4
99- 105 region

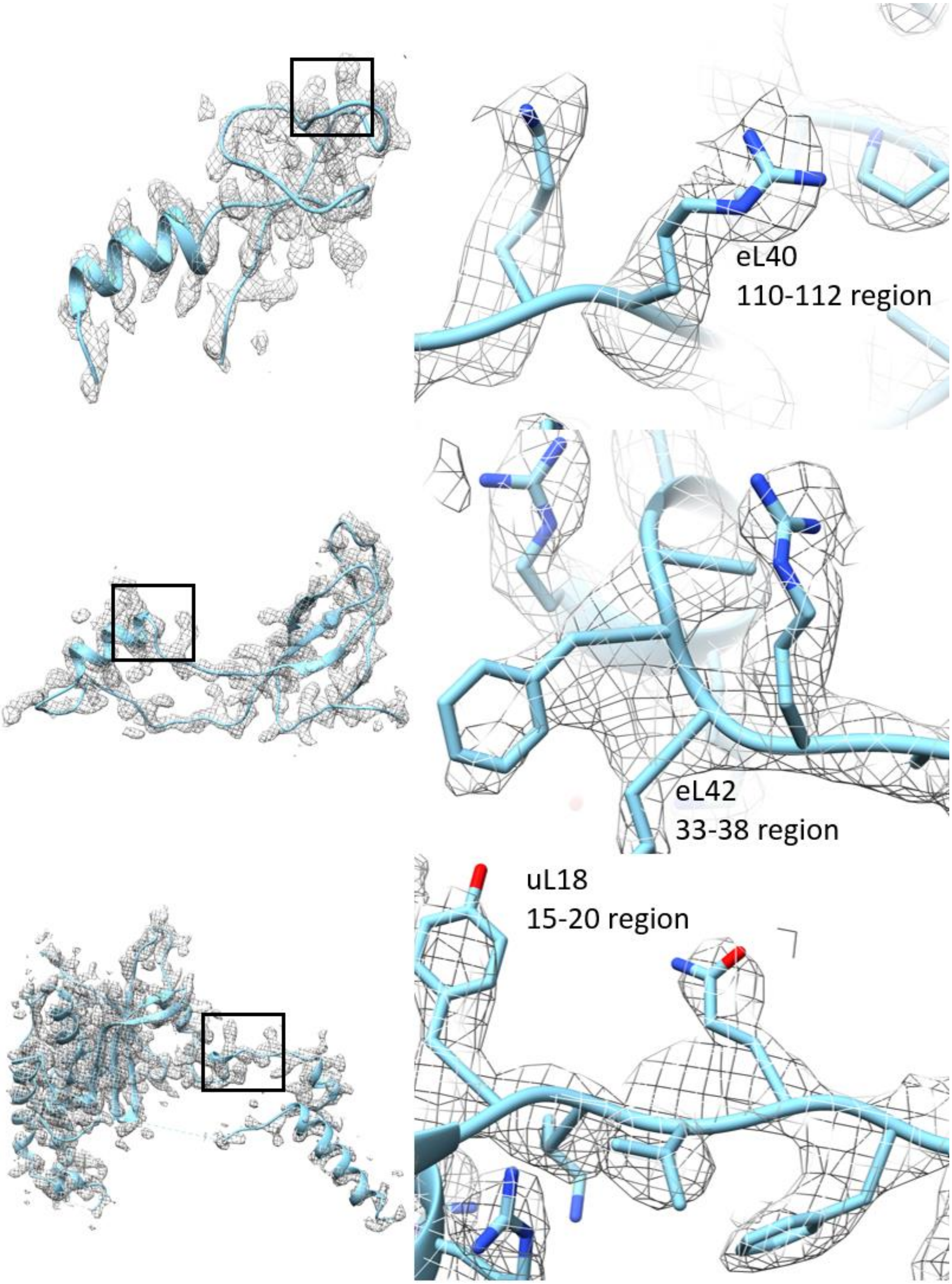

eL40
110-112 region
eL42
33-38 region
uL18
15-20 region

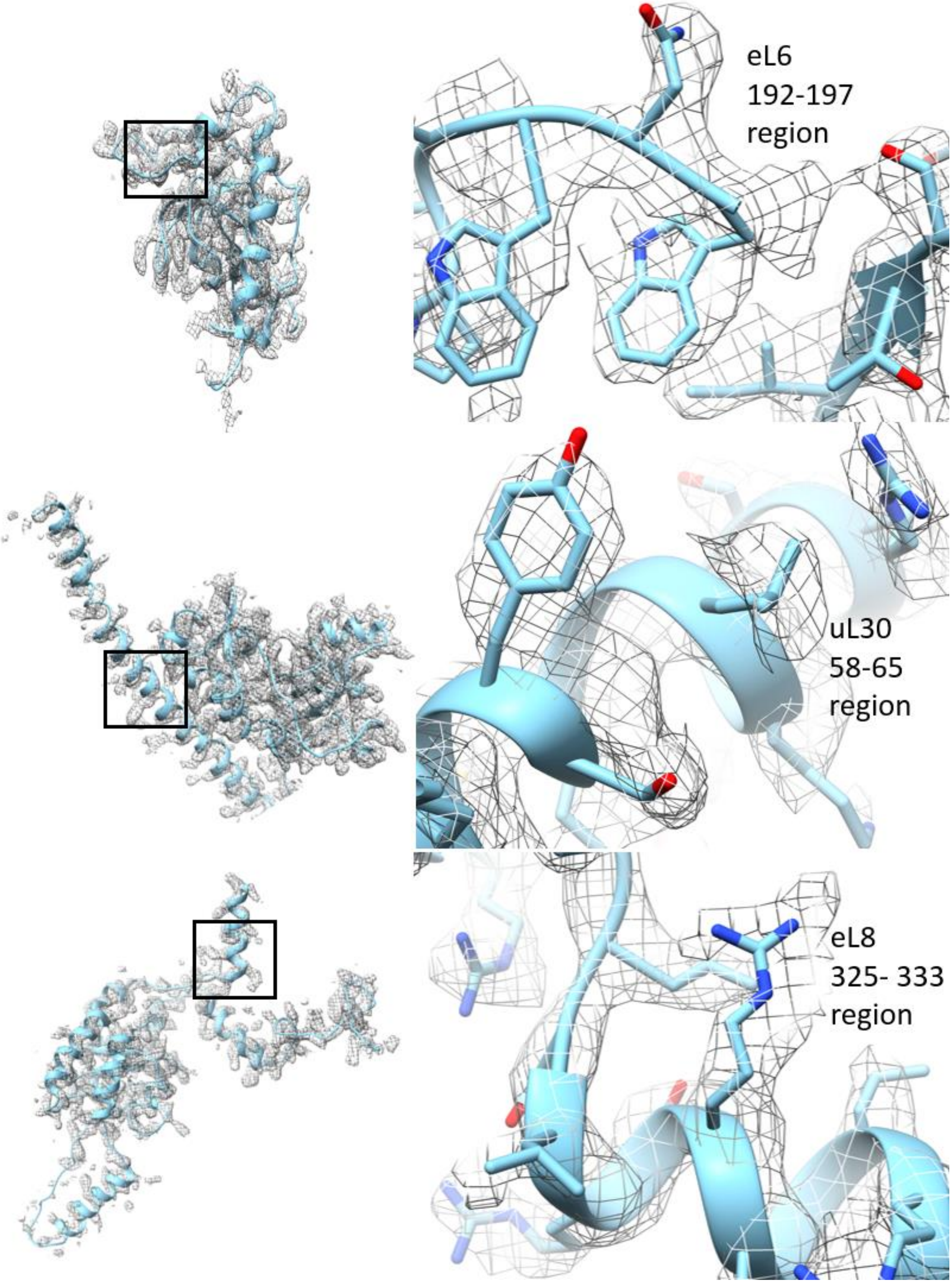
eL6
192-197
region
uL30
58-65
region
eL8
325- 333
region

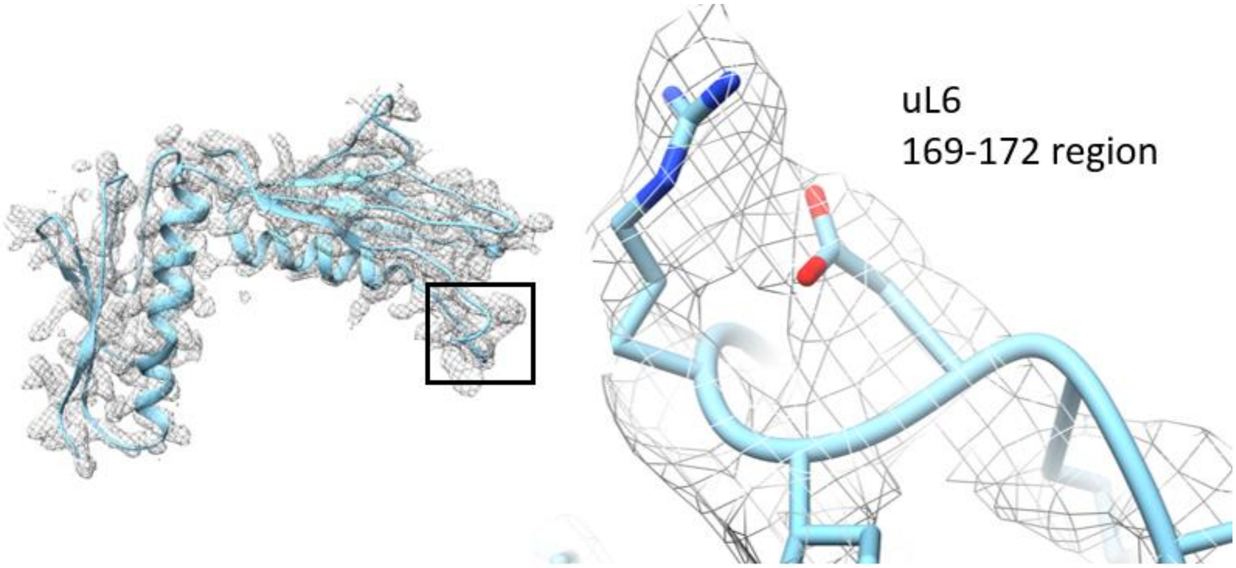

Supplementary Figure 3: Density fits of modeled LSU proteins and selected regions showcasing high-resolution features. Locations of selected regions on the protein are indicated by black box and correspond to the high-resolution features displayed adjacent to the protein. Region numberings correspond to amino acid sequence.

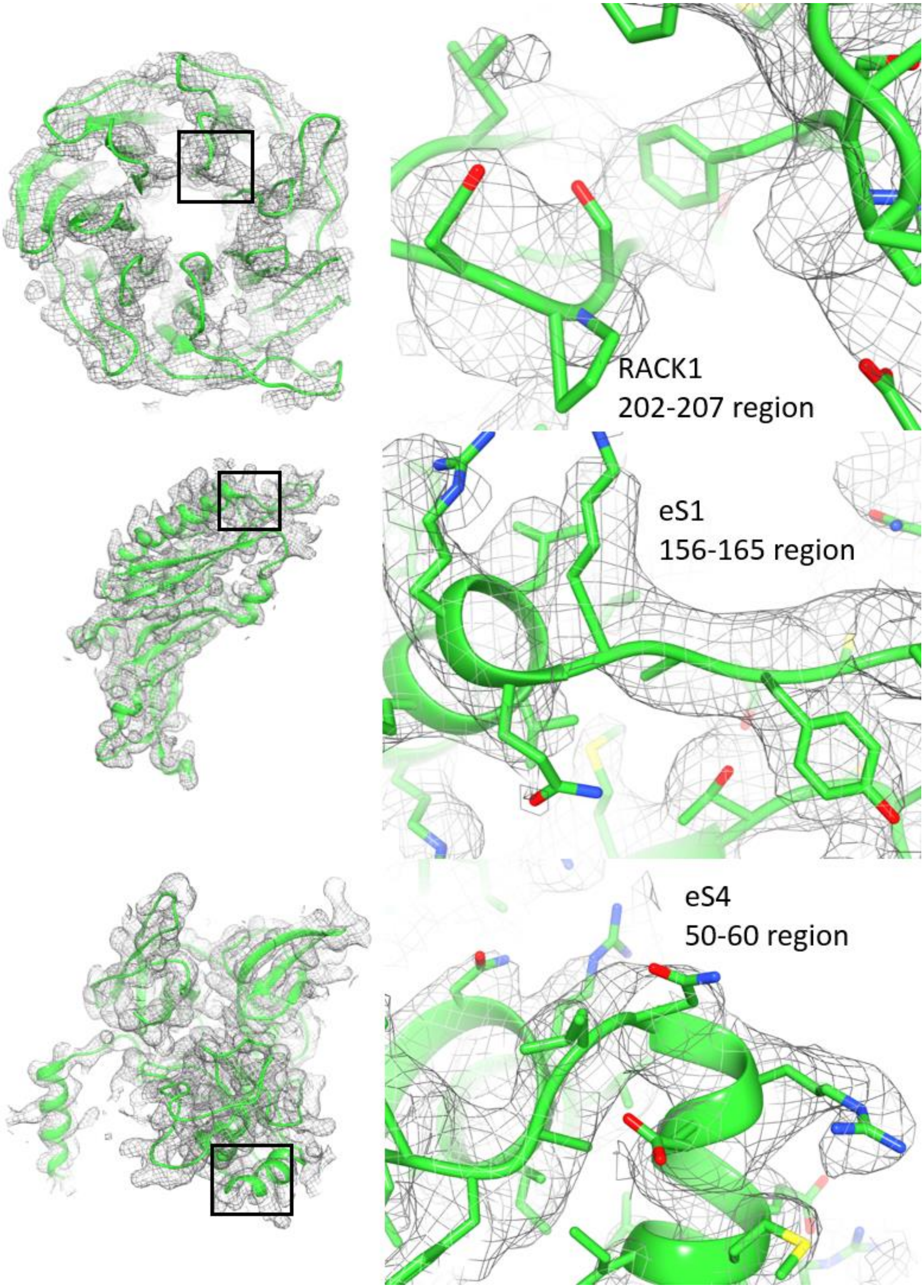

RACK1
202-207 region
eS1
156-165 region
eS4
50-60 region

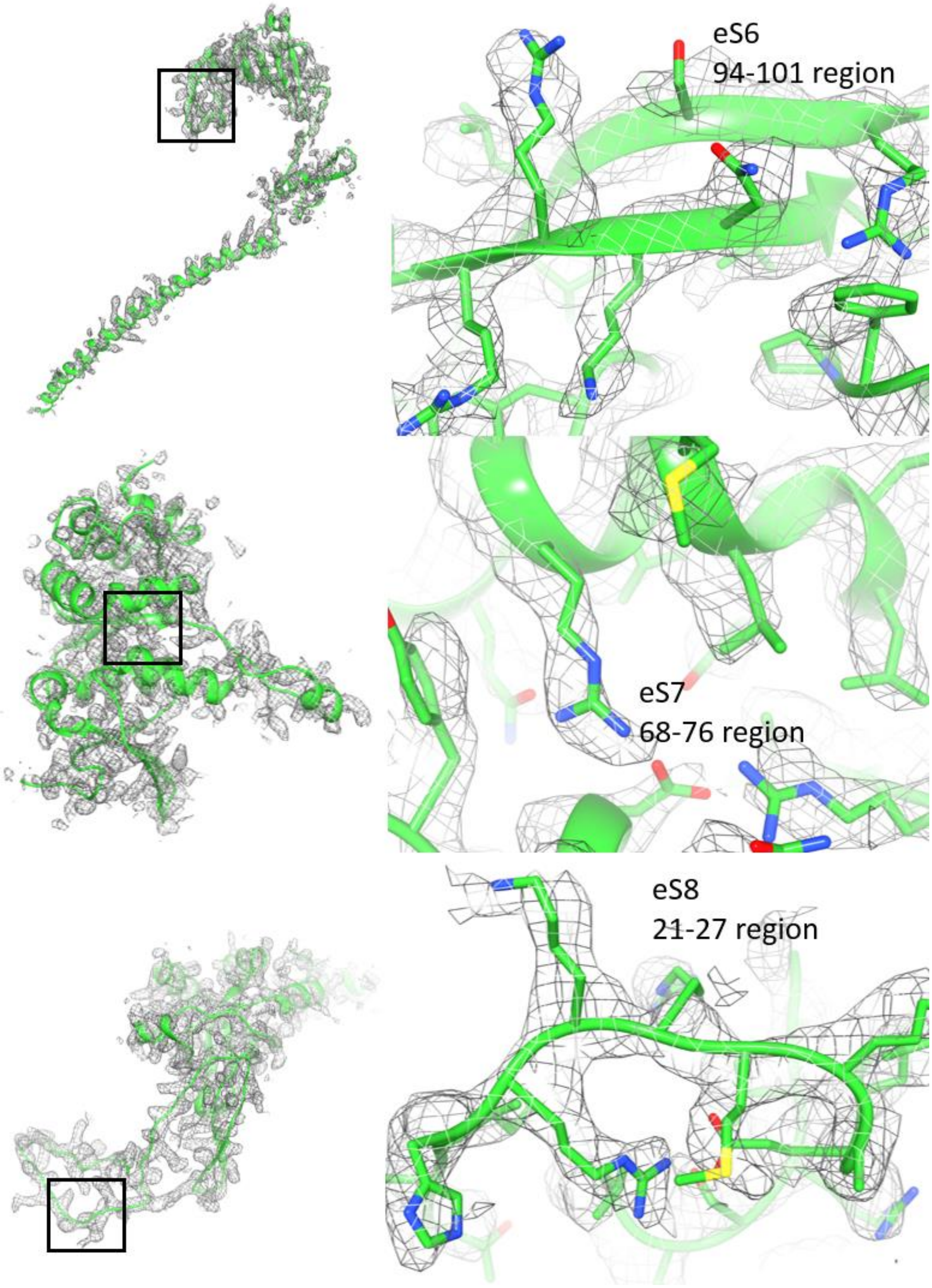
eS6
94-101 region
eS7
68-76 region
eS8
21-27 region

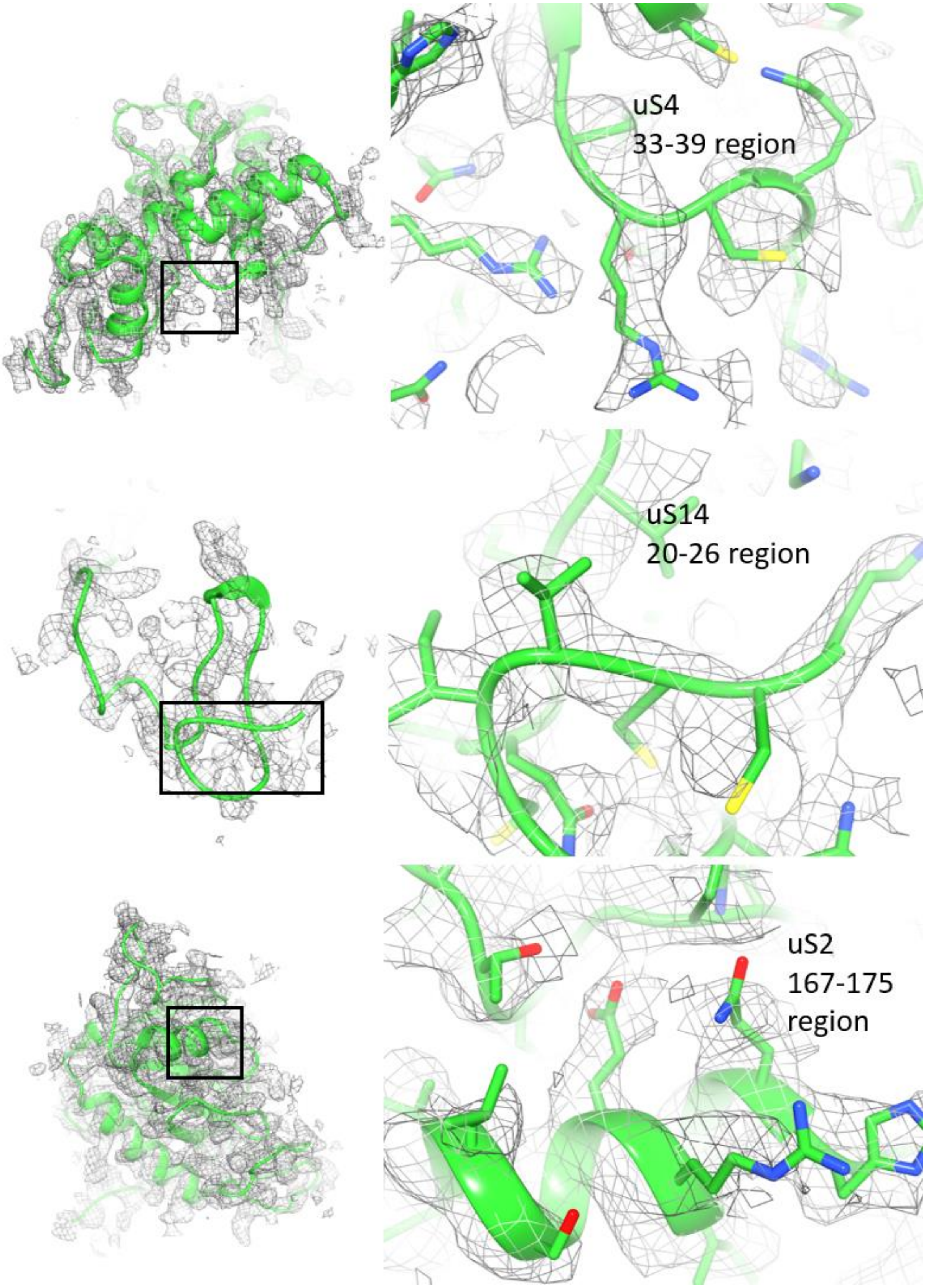
uS4
33-39 region
uS14
20-26 region
uS2
167-175
region

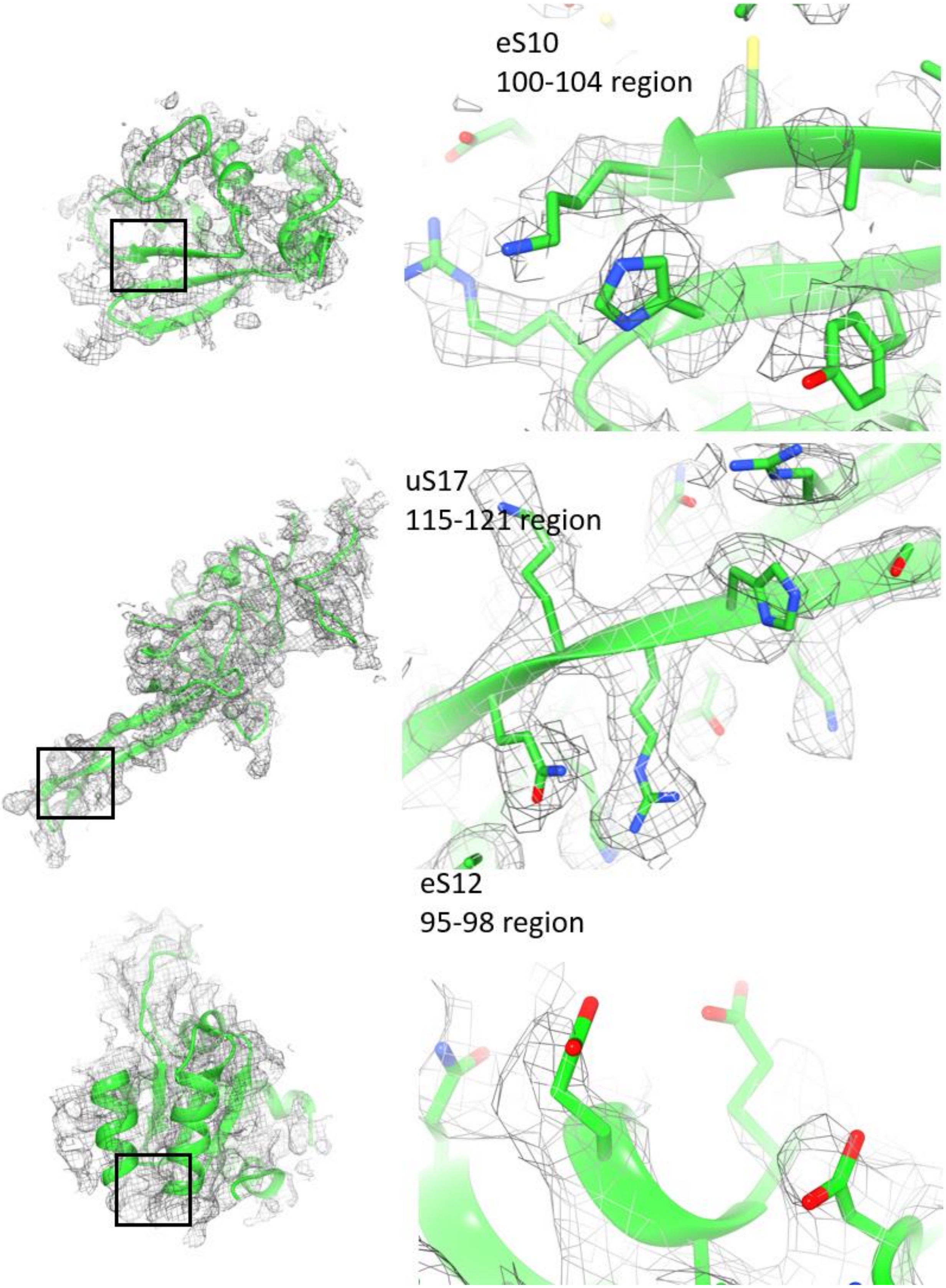
eS10
100-104 region
uS17
115-121 region
eS12
95-98 region

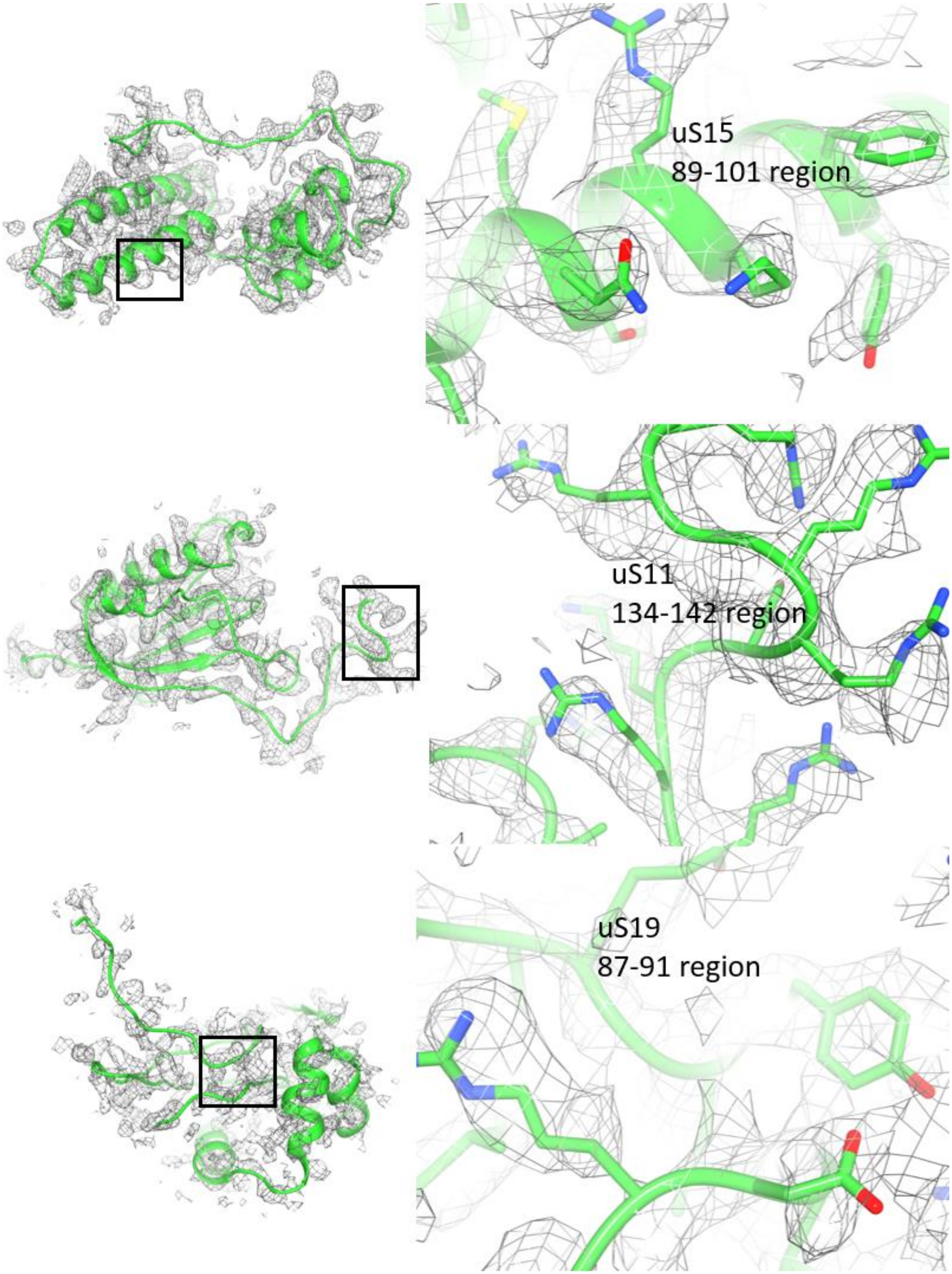
uS15
89-101 region
uS11
134-142 region
uS19
87-91 region

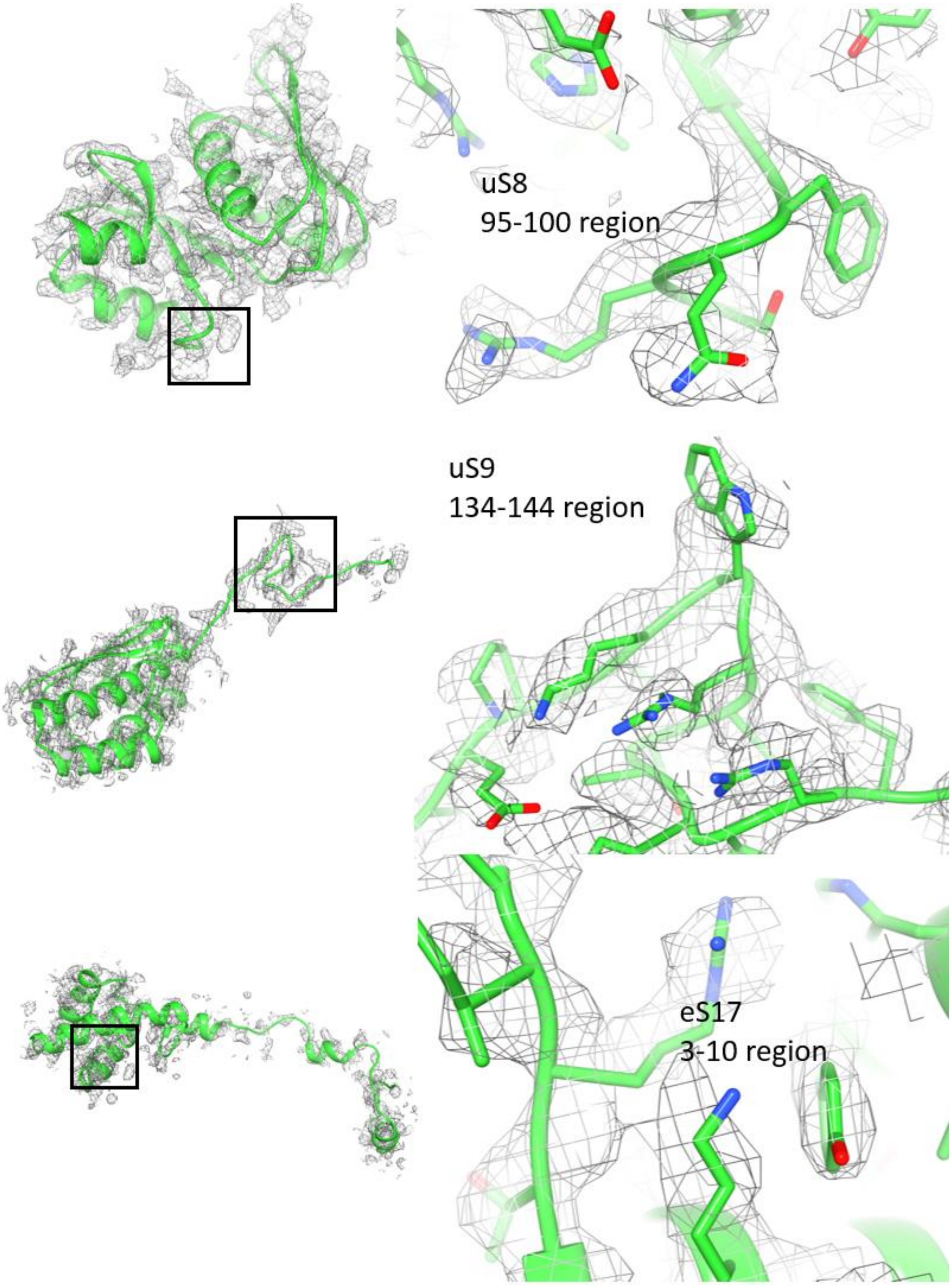
uS8
95-100 region
uS9
134-144 region
eS17
3-10 region

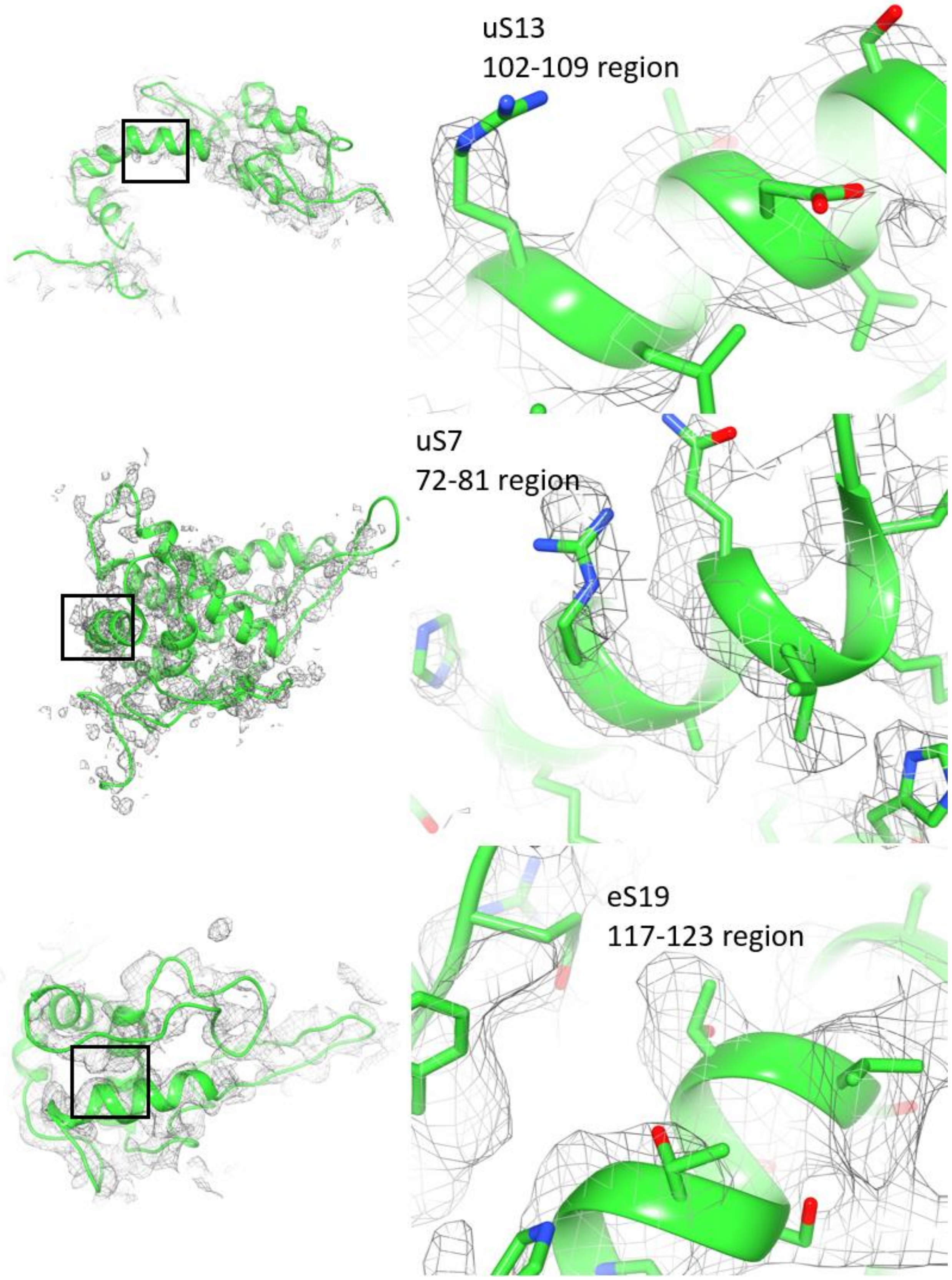
uS13
102-109 region
uS7
72-81 region
eS19
117-123 region

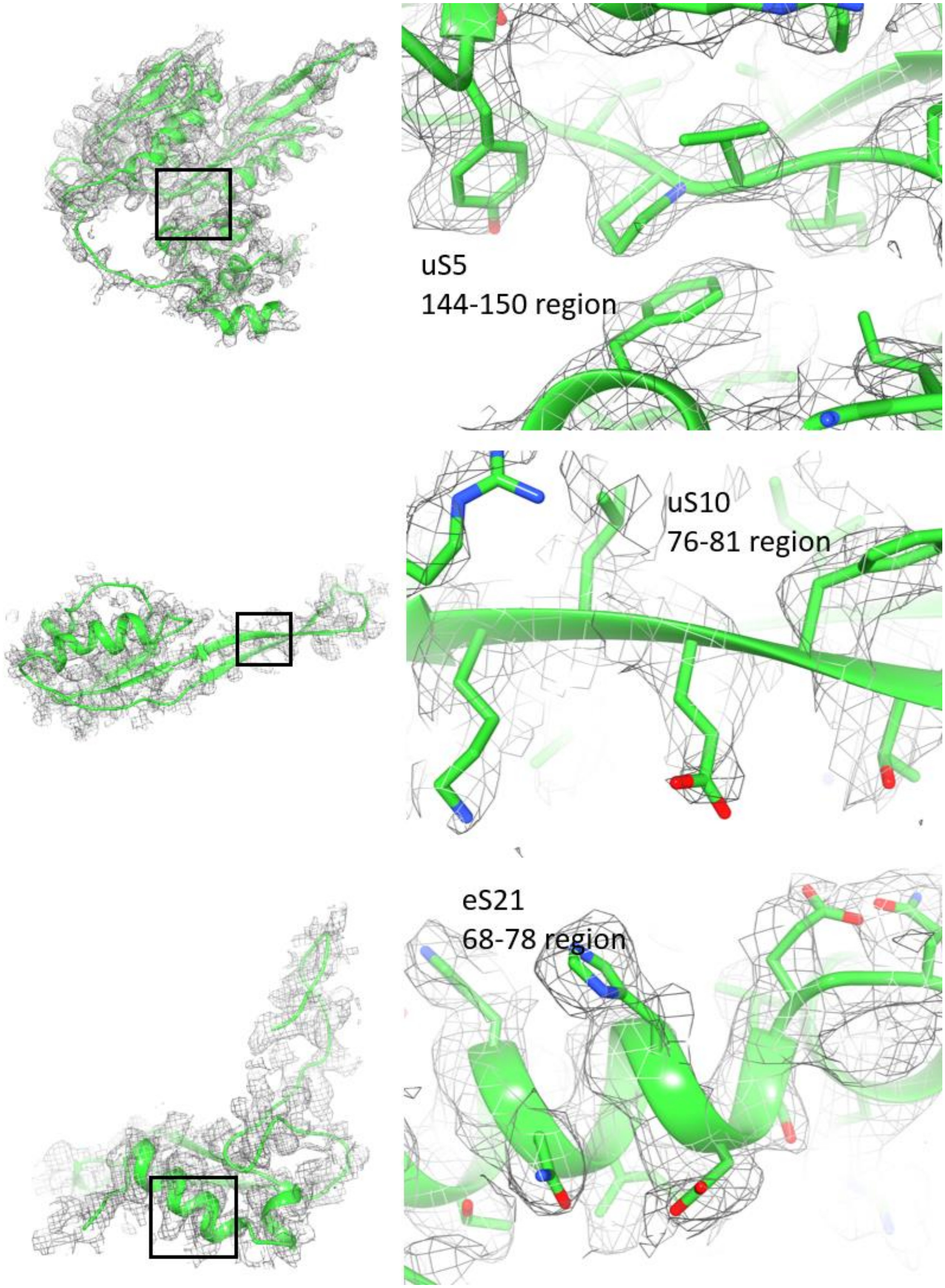
uS5
144-150 region
uS10
76-81 region
eS21
68-78 region

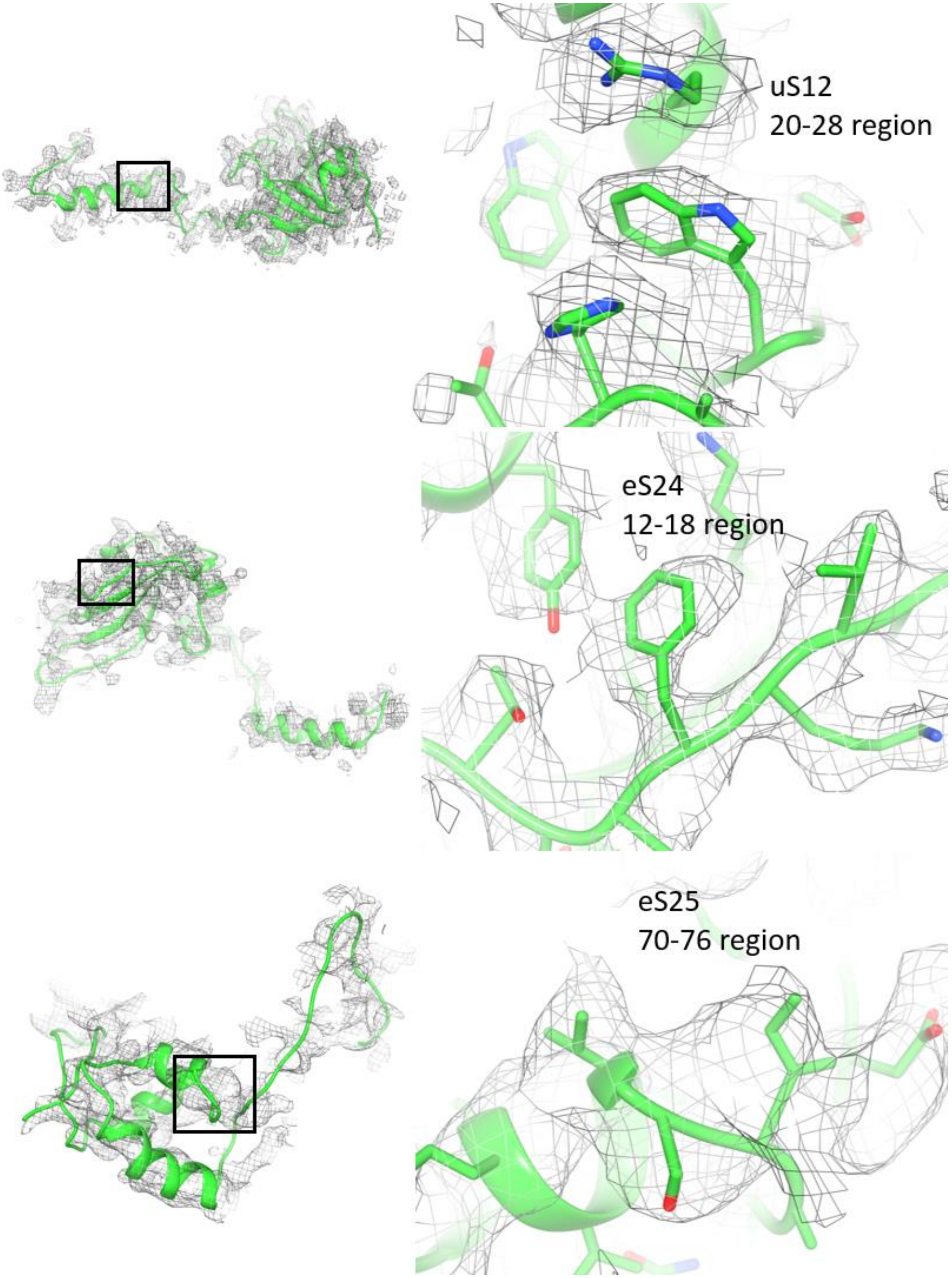
uS12
20-28 region
eS24
12-18 region
eS25
70-76 region

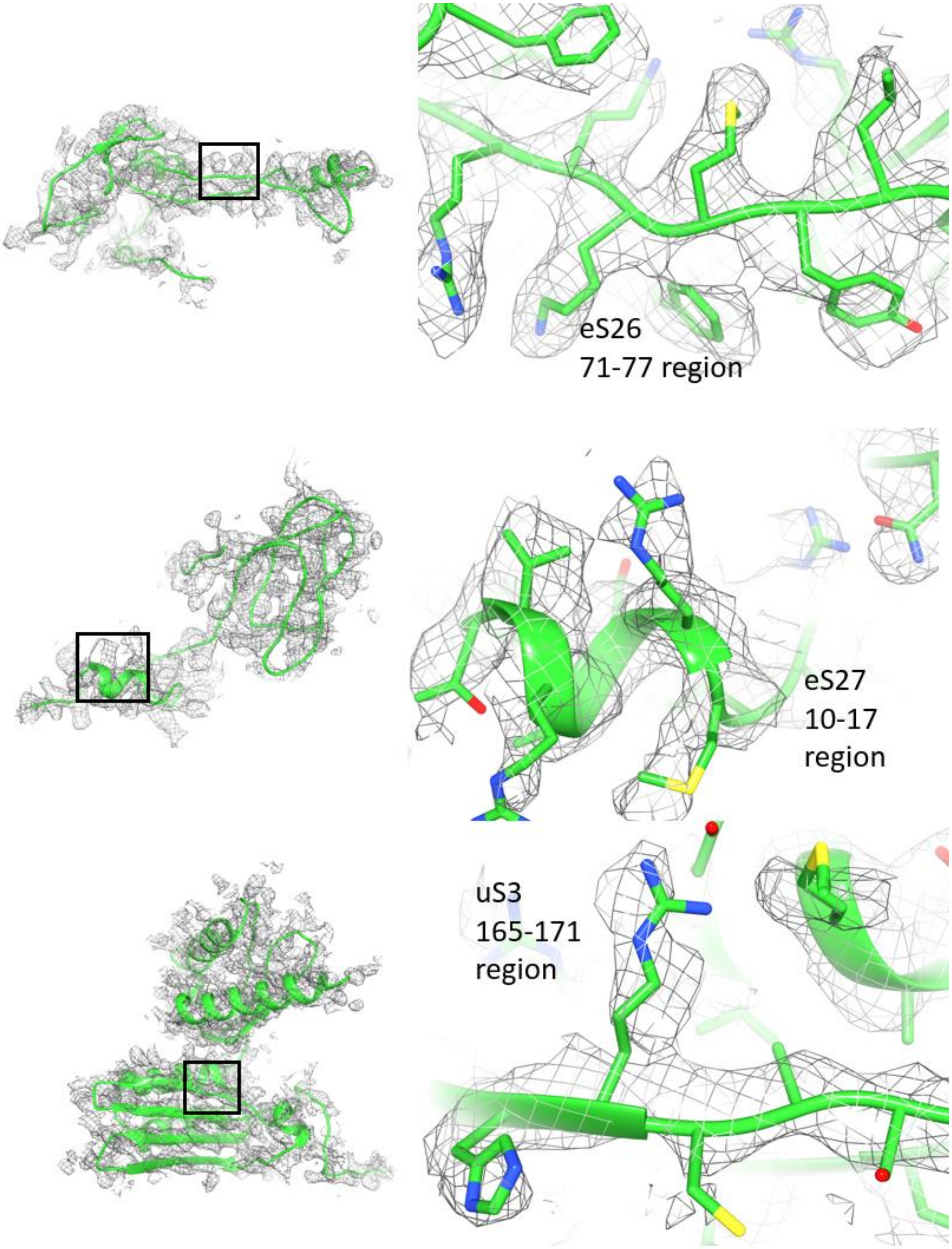
eS26
71-77 region
eS27
10-17
region
uS3
165-171
region

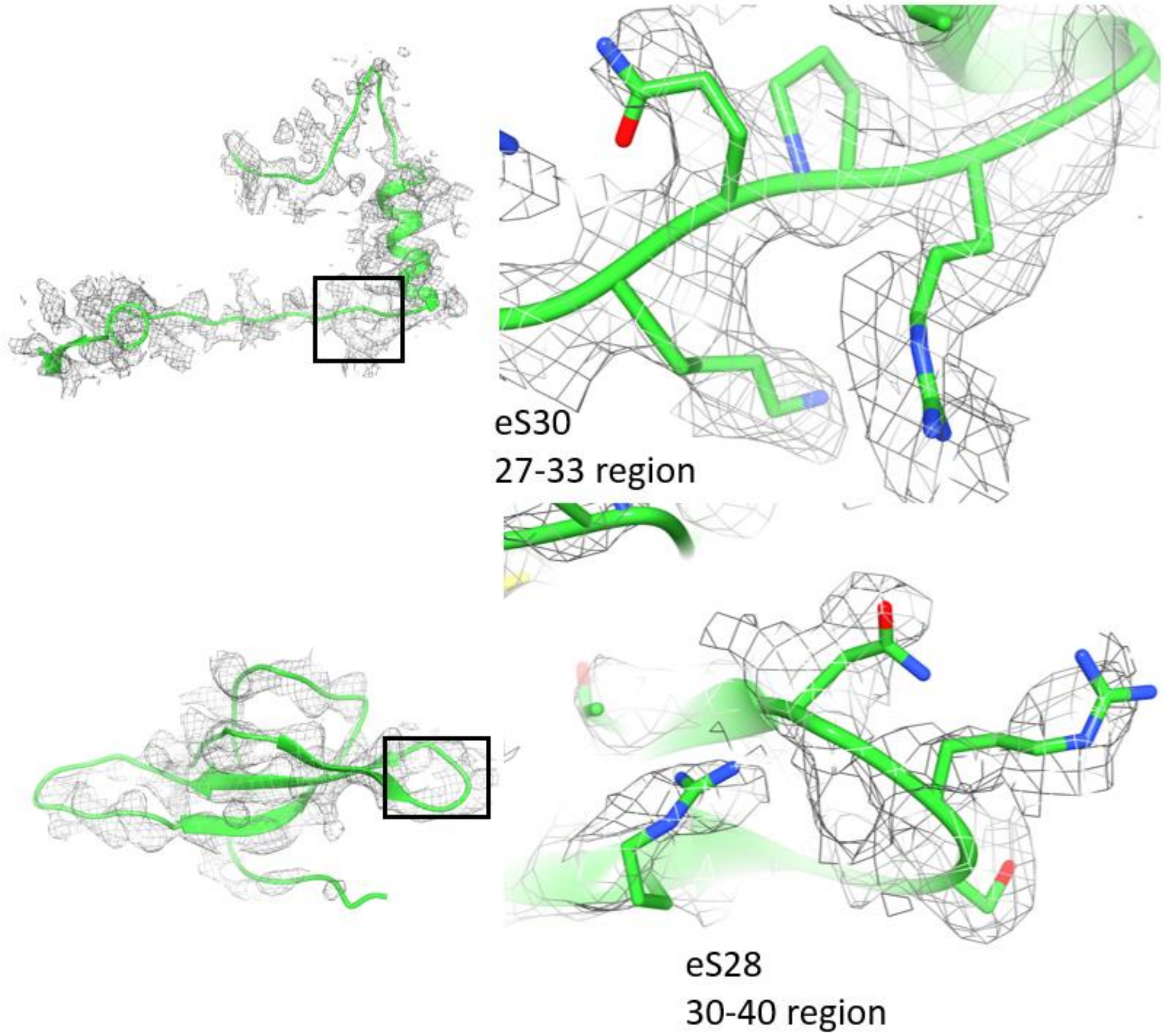

Supplementary Figure 4: Density fits of modeled SSU proteins and selected regions showcasing high-resolution features. Locations of selected regions on the protein are indicated by a black box and correspond to the high-resolution features displayed adjacent to the protein. Region numberings correspond to amino acid sequence.

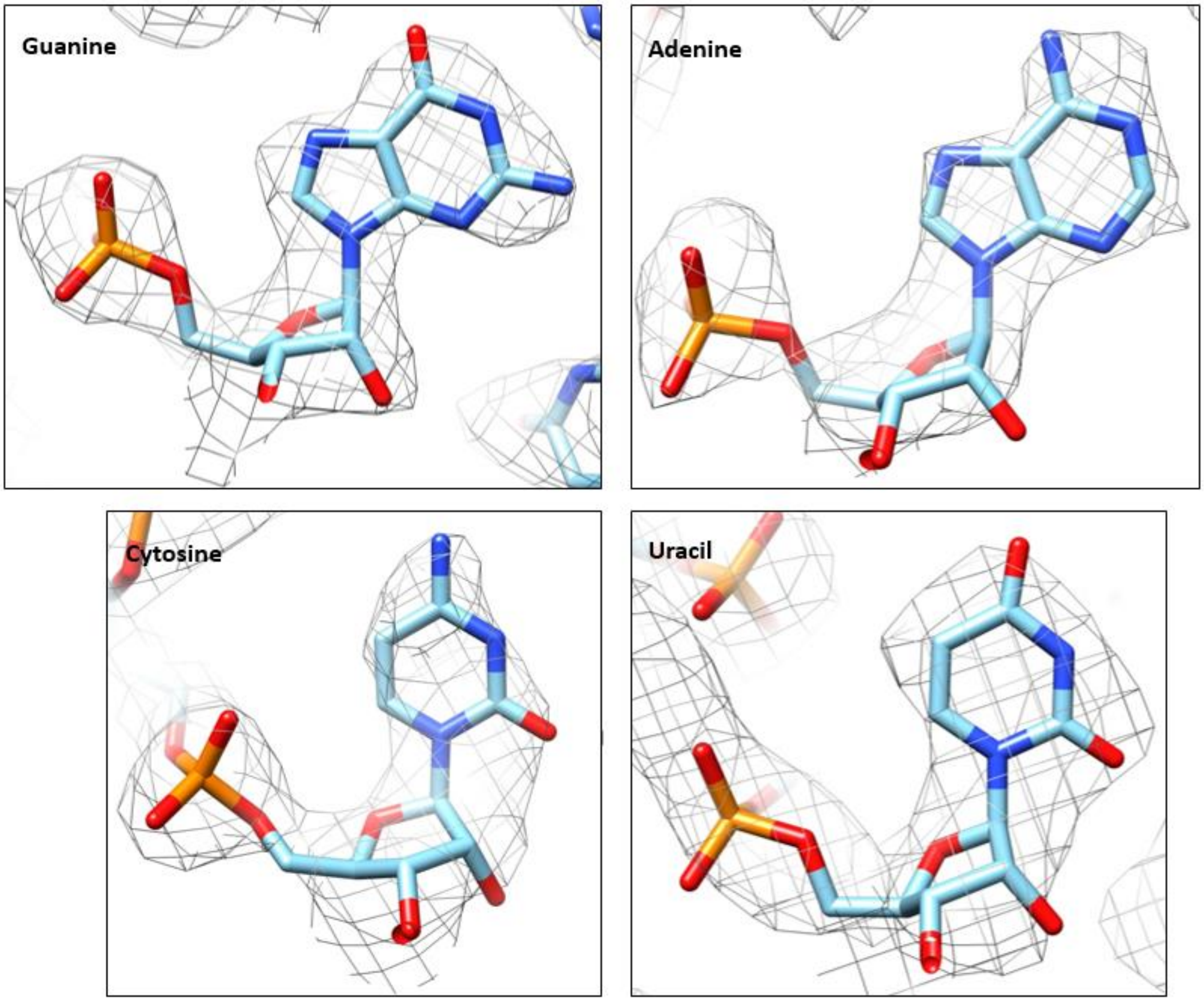

Supplementary Figure 5: Density of rRNA showcasing identification of individual nucleotides in the ribosome of *L. donovani*.

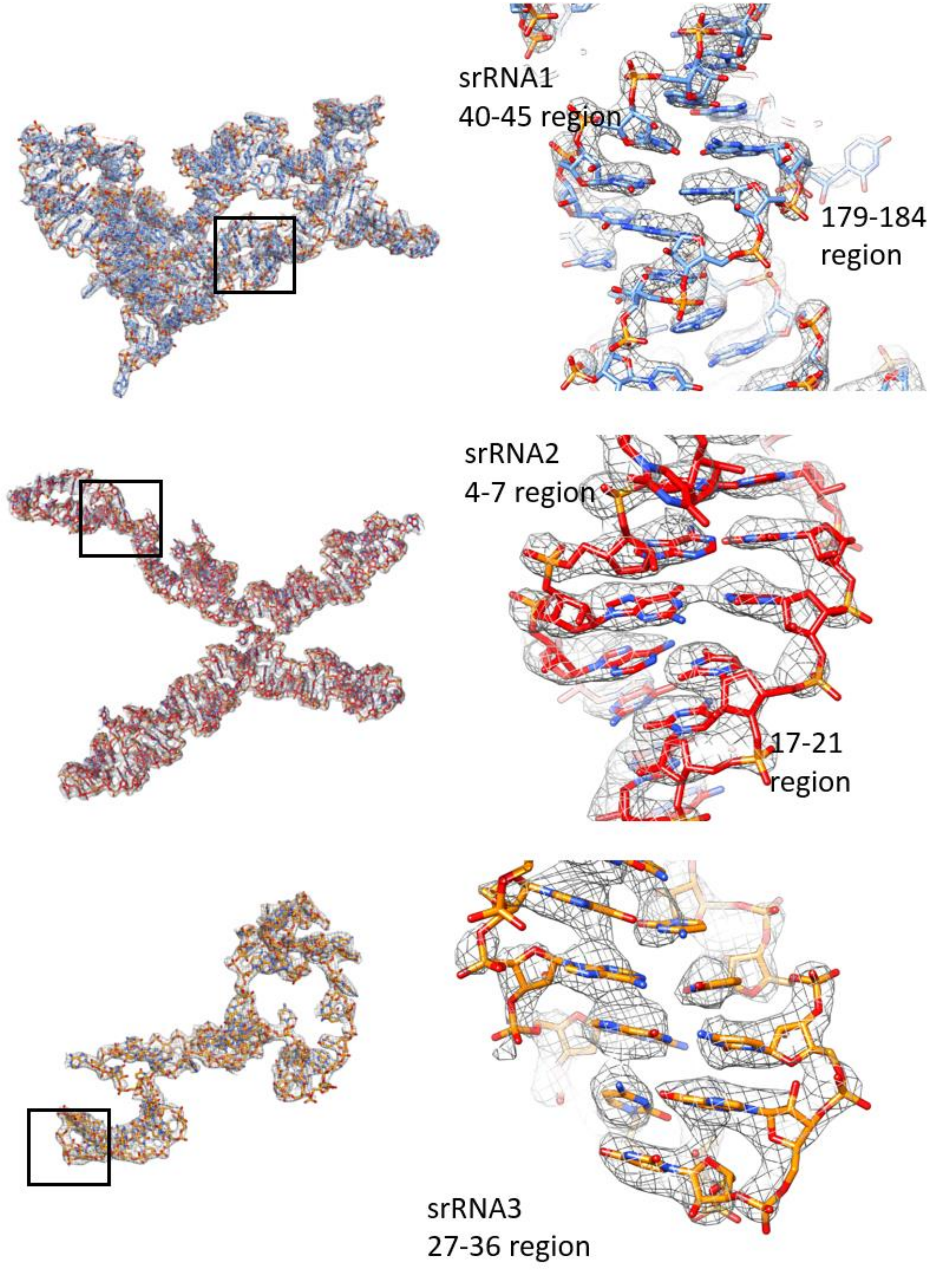
srRNA1
40-45 region
179-184
region
srRNA2
4-7 region
17-21
region
srRNA3
27-36 region

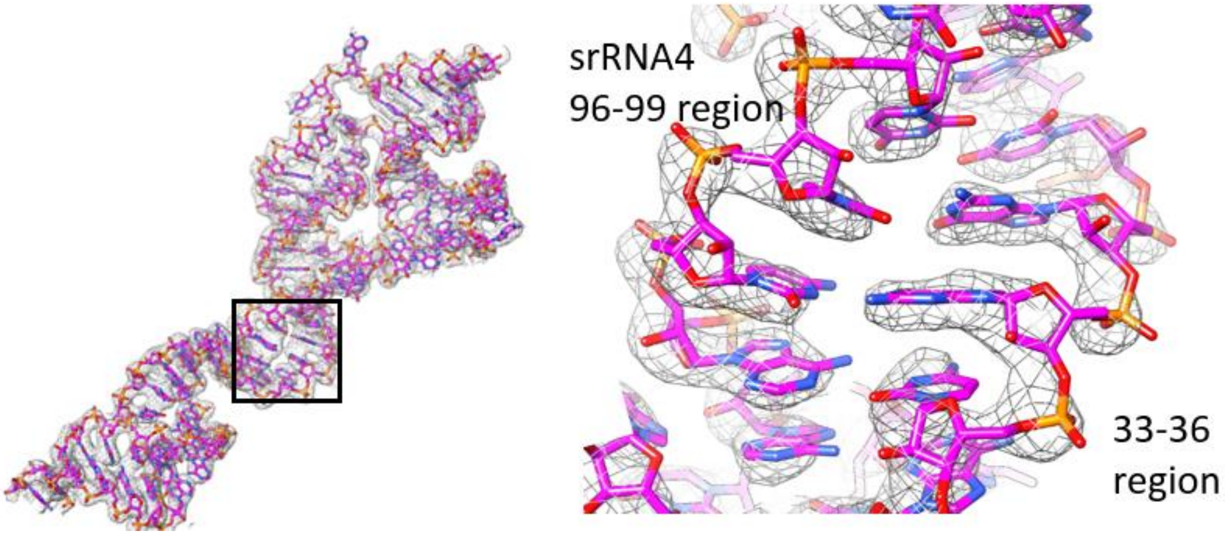
srRNA4
96-99 region
33-36
region

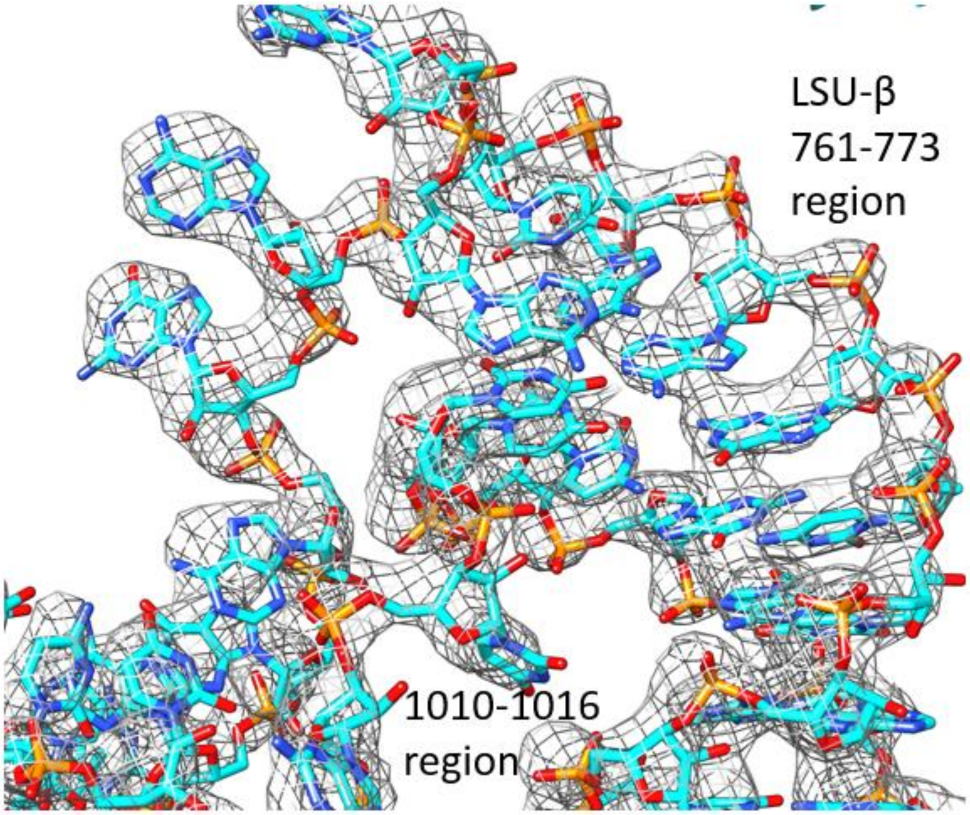
LSU-β
761-773
region
1010-1016
region

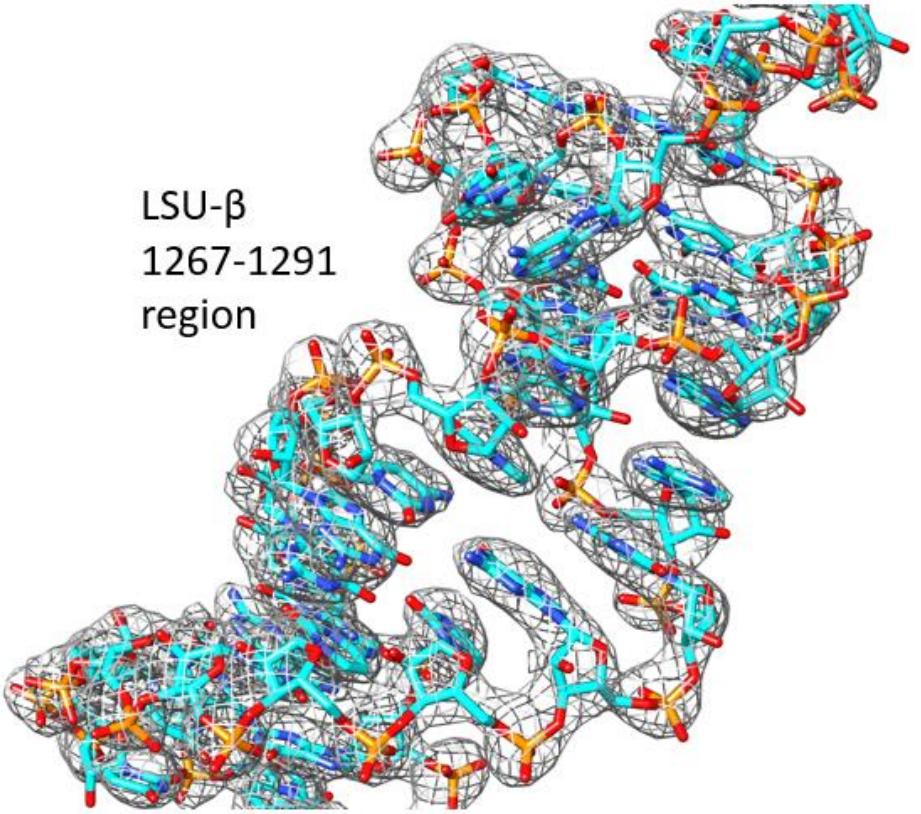
LSU-β
1267-1291
region

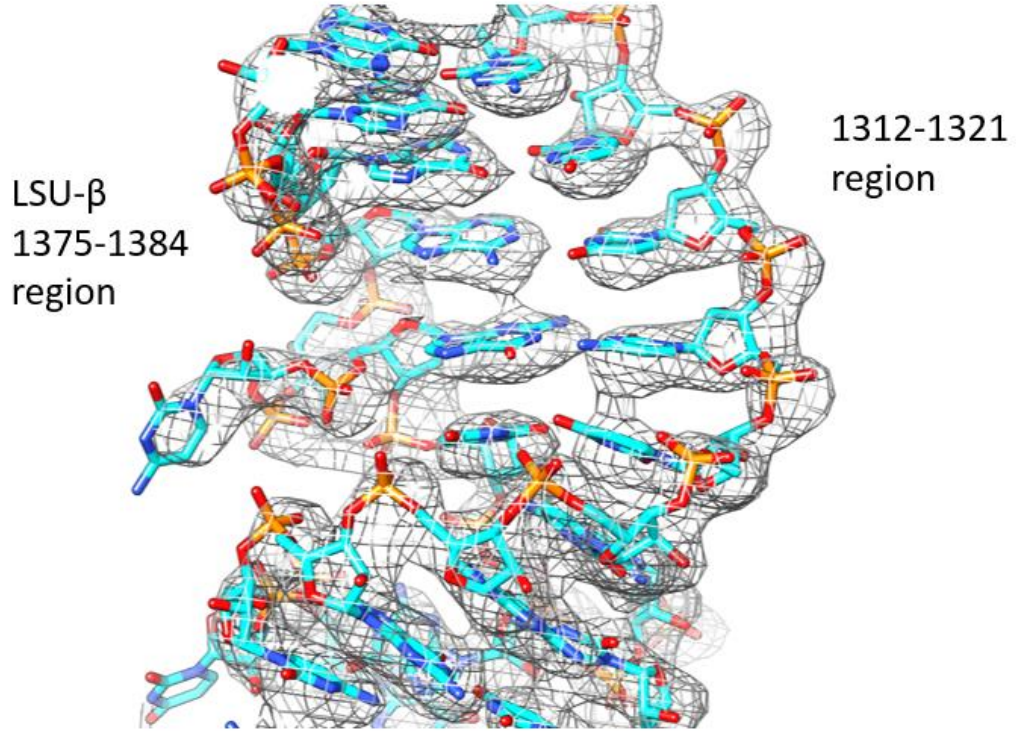

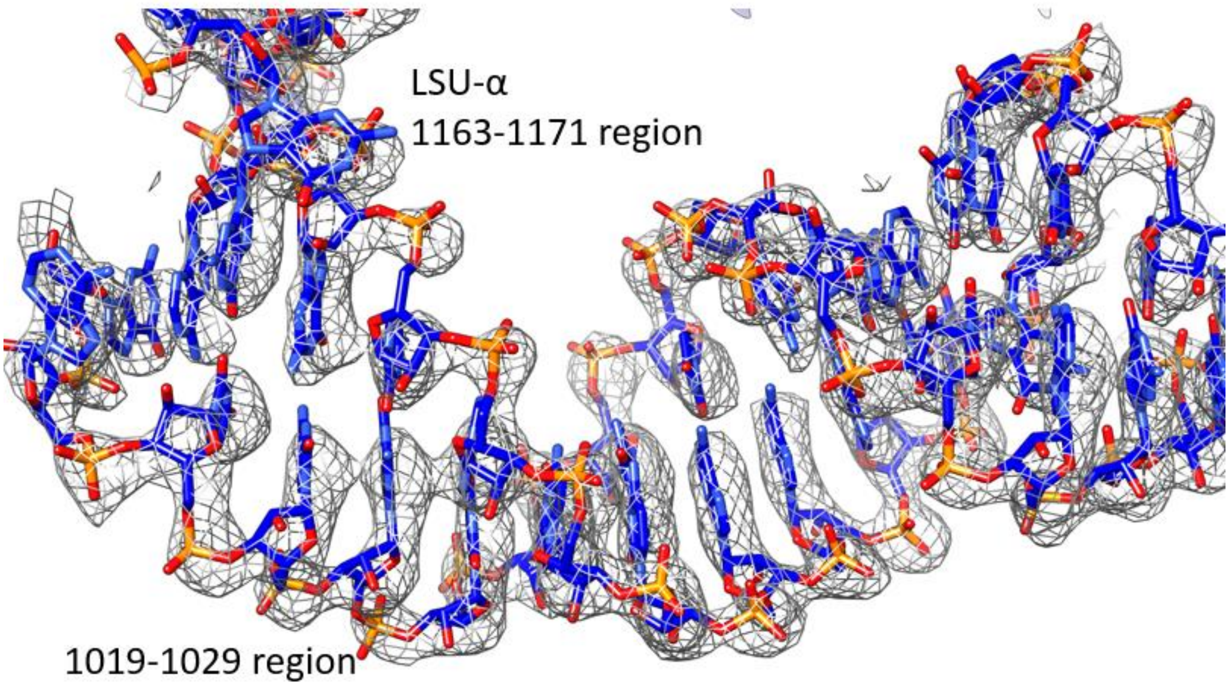

Supplementary Figure 6: Density fits of LSU trypanosomal-unique rRNAs with selected regions showcasing high-resolution features. Locations of selected regions on the rRNA are indicated by a black box and correspond to the high-resolution features displayed adjacent to the srRNA. Selected LSU-β and LSU-α densities shown also. Region numberings correspond to nucleotide sequence.

a
b
c
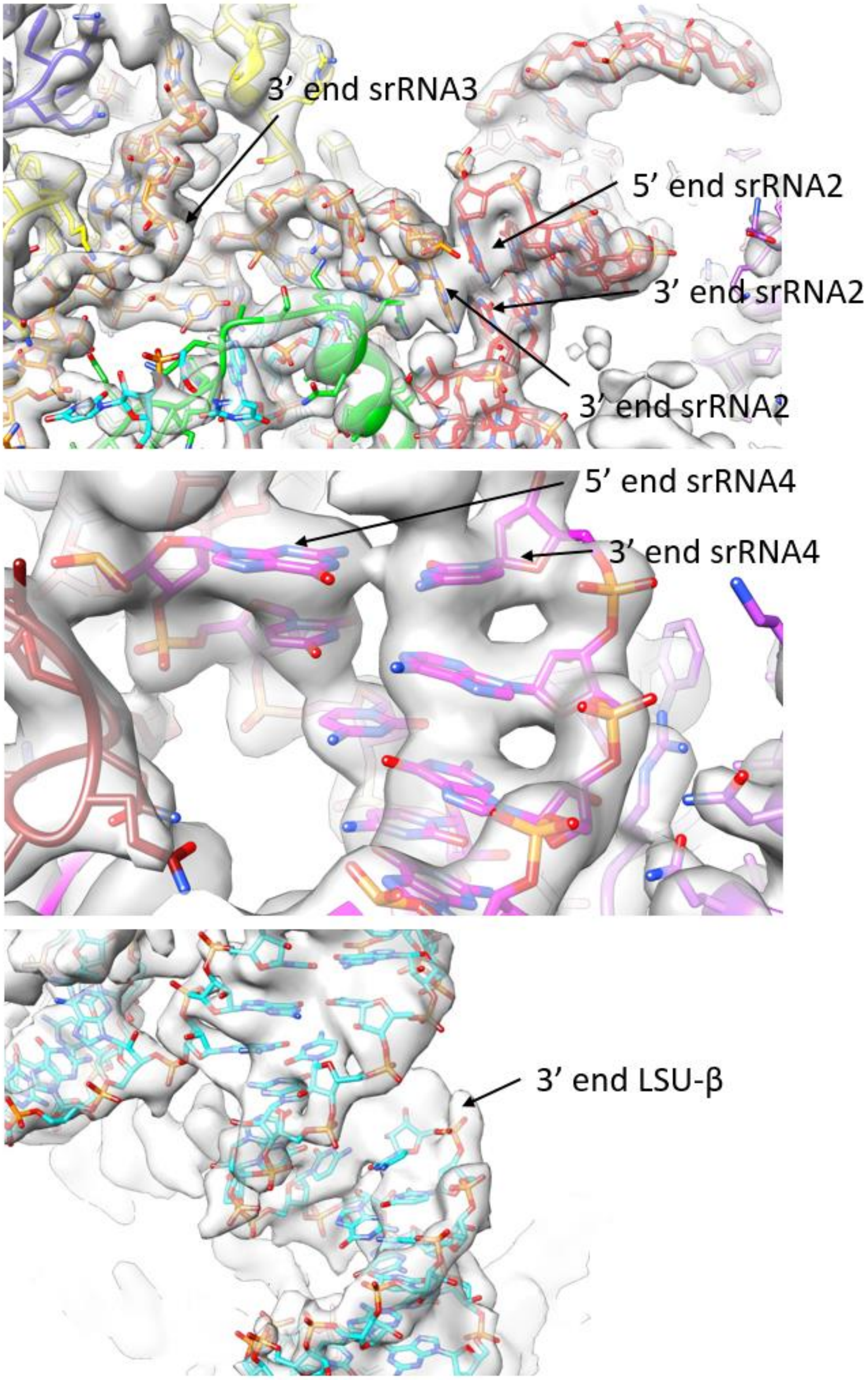
3’ end srRNA3
5’ end srRNA2
3’ end srRNA2
3’ end srRNA2
5’ end srRNA4
3’ end srRNA4
3’ end LSU-β

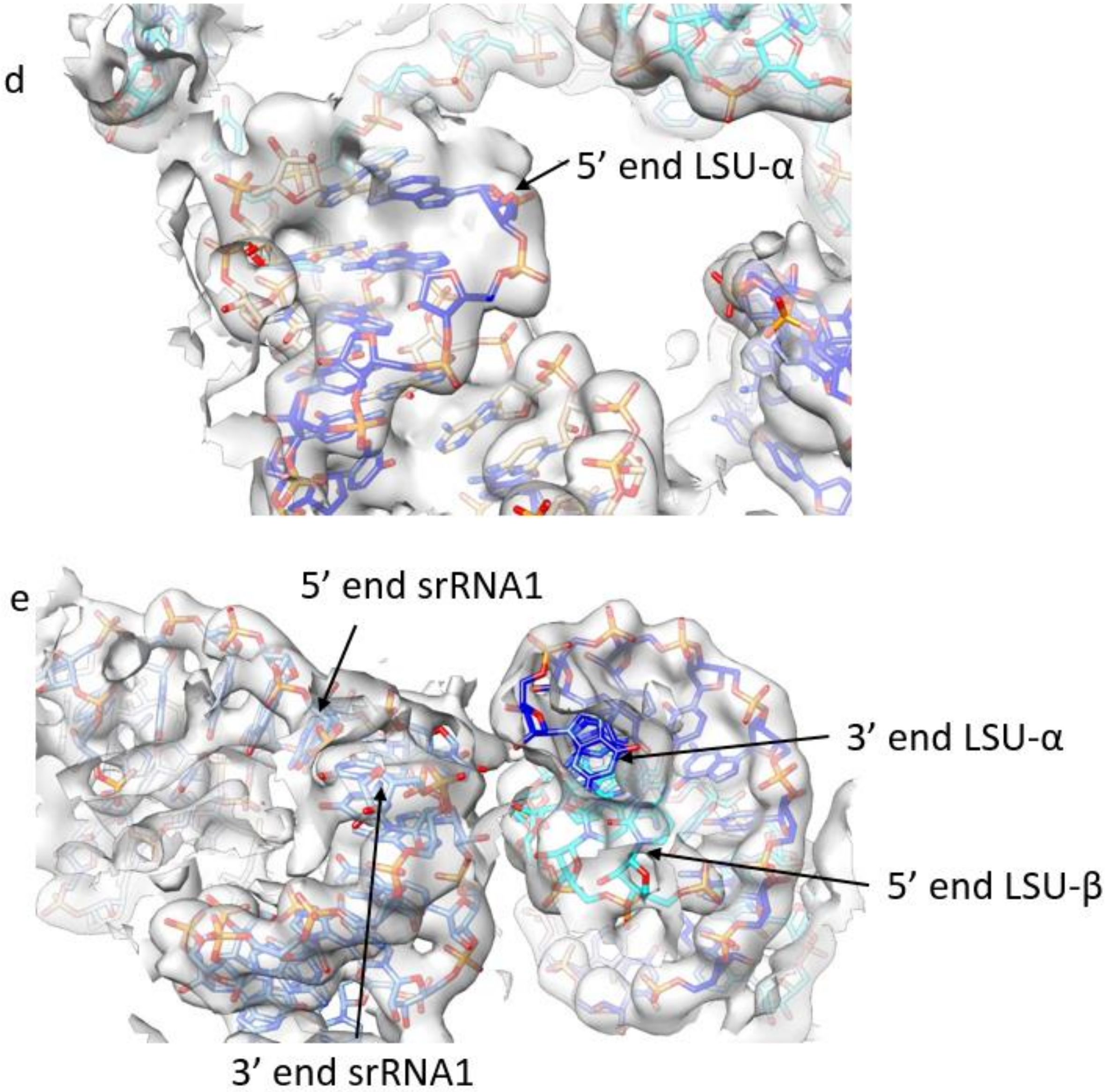

Supplementary Figure 7: Full model with density highlighting various rRNA components in *L. donovani*. (a) Region surrounding both ends of srRNA2 and srRNA3. (b) Region surrounding both ends of srRNA4. (c) Region surrounding the 3’ end of LSU-β. (d) Region surrounding the 5’ end of LSU-α (e) Region surrounding both ends of srRNA1, the 5’ end of LSU-β, and the 3’ end LSU-α.

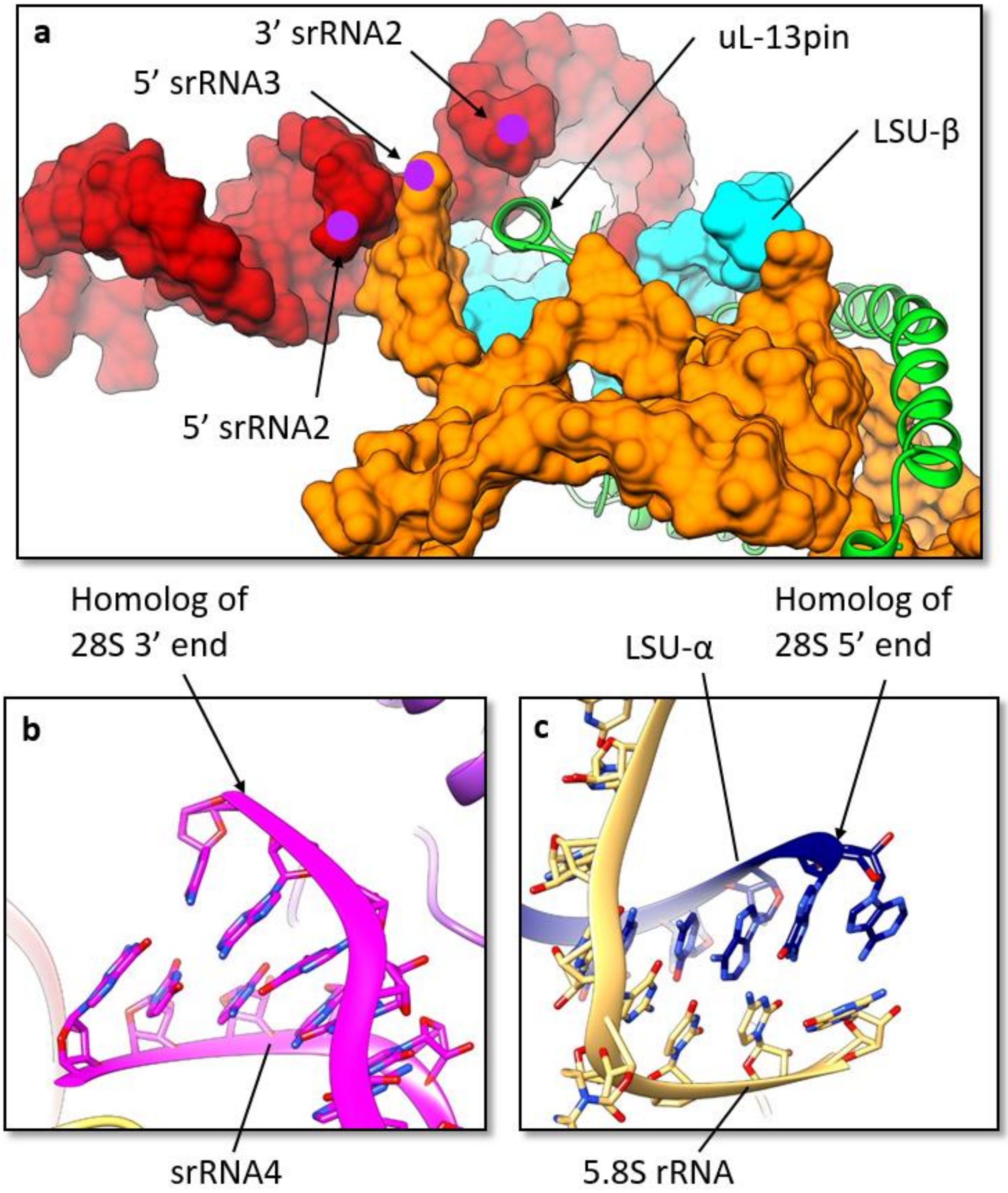

Supplementary Figure 8: View of uL13-pin and rRNA fragment pinned ends as compared to basepair stabilized rRNA ends. (a) Three sided binding pocket formed by srRNA2(red), srRNA3(orange), and LSU-β(cyan) of uL-13pin(green). rRNA fragment ends are highlighted by magenta circles. KSD is hidden for clarity. (b-c) Basepair stabilization of the 5’ and 3’ LSU rRNA ends homologous to the ends of 28S rRNA in non-trypanosomal ribosomes.

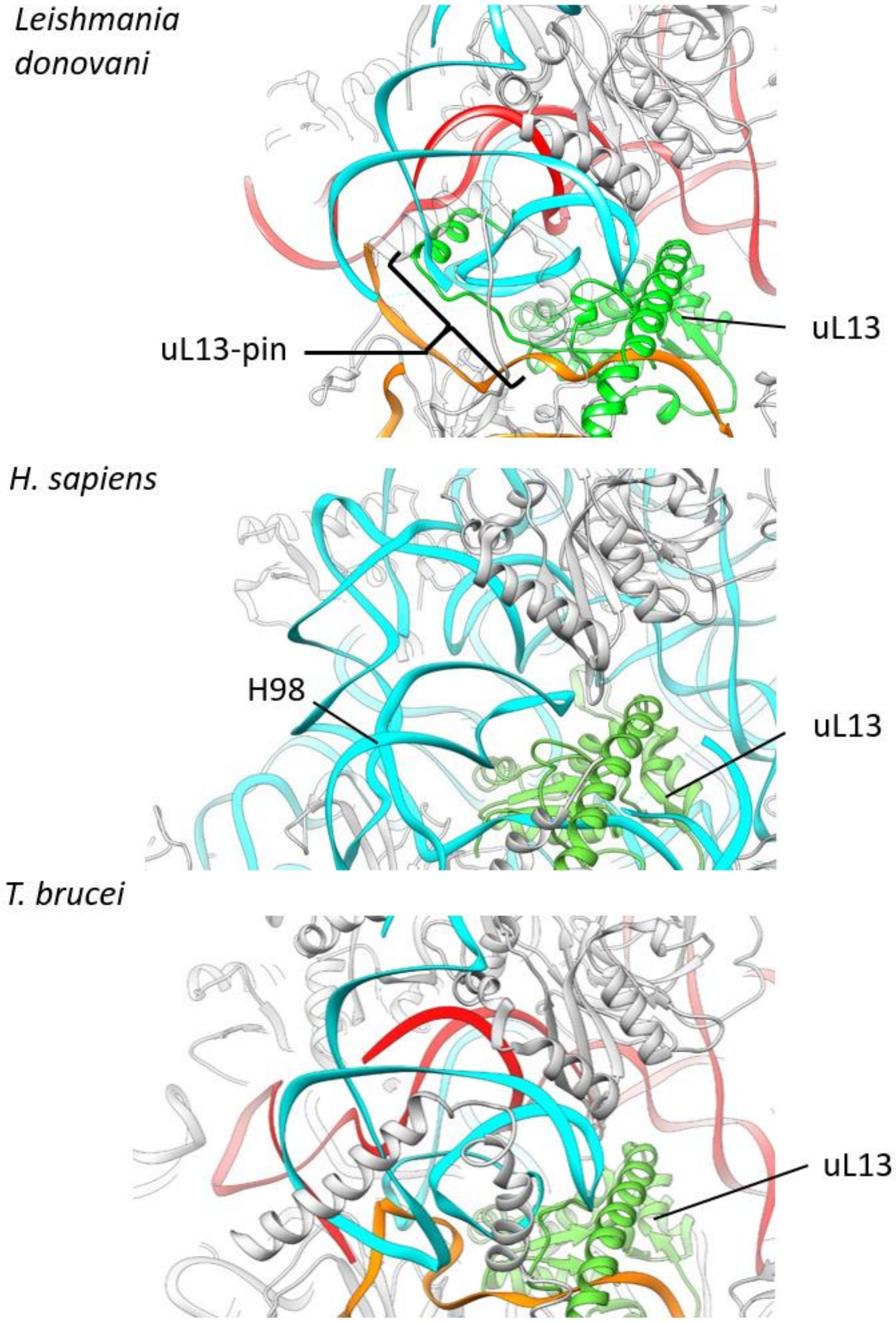

Supplementary Figure 9: Comparison of the uL13-pin region between *L. donovani,* human, and *T. brucei*[3] ribosomes.

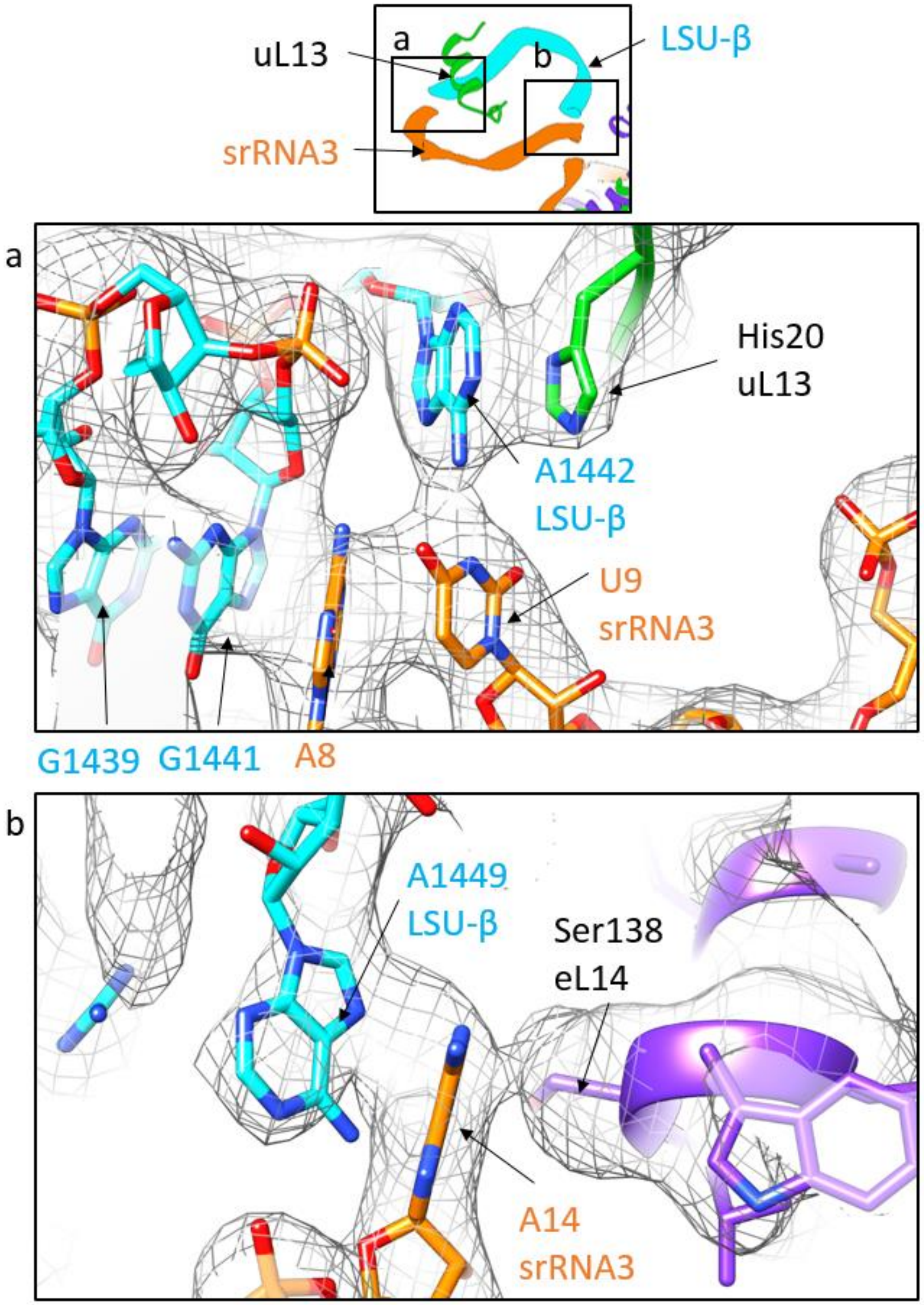

Supplementary Figure 10: rRNA ring formation details. Schematic of the ring formed by LSU-β (cyan) and srRNA3 (orange) through interactions with uL13 and eL14. (a) Multiple stacking interactions between bases of LSU-β and srRNA3, and A1442 of LSU-β and His20 of uL13 along with an interaction between A1442 of LSU-β and U9 of srRNA3 closing the other side of the rRNA ring shown in Fig. 6b. (b) LSU-B and srRNA3 interacting with eL14 to close one side of the rRNA ring shown in Fig. 6b.

a

b

c

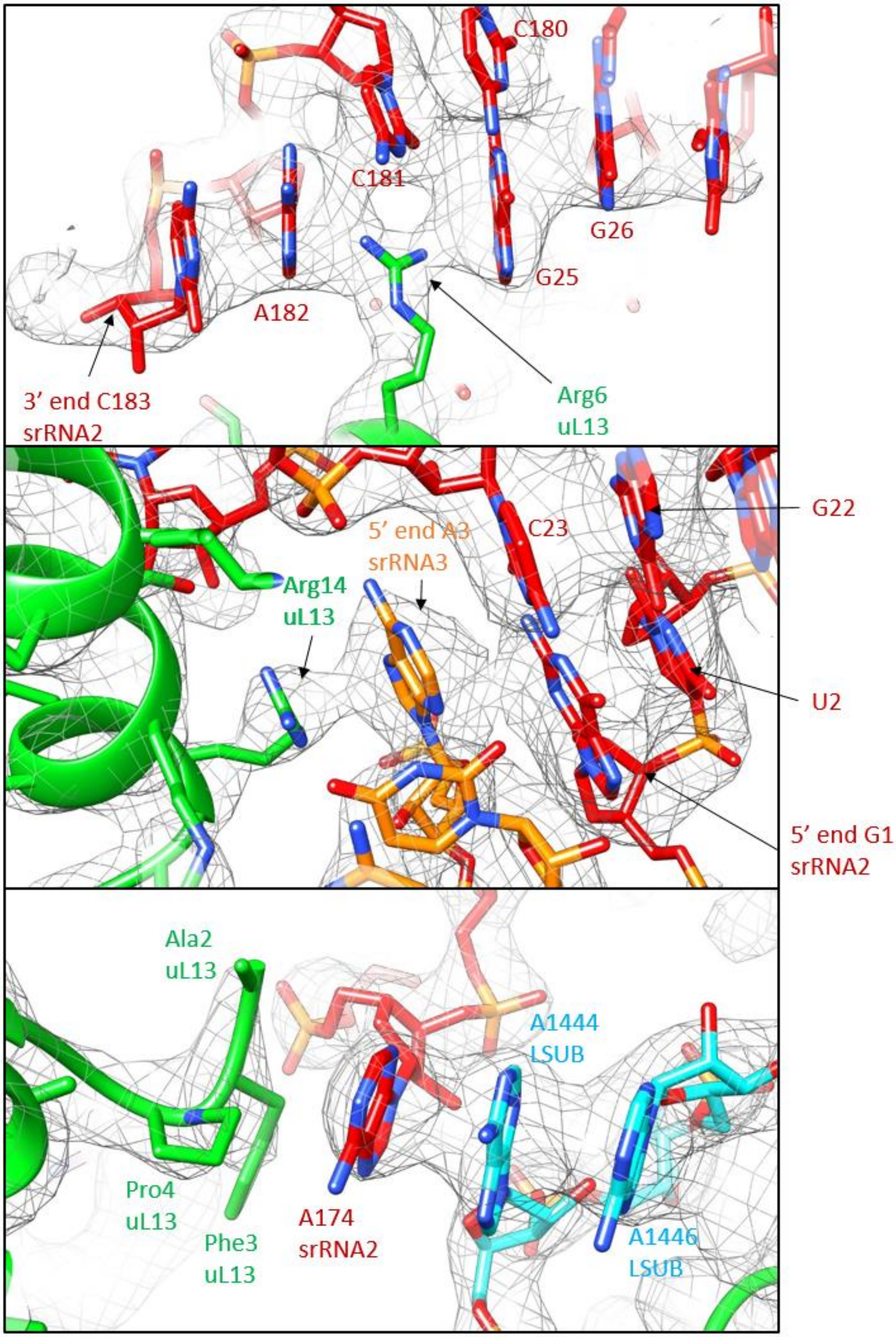

C180
C181
G26
G25
A182
3' end C183
srRNA2
Arg6
uL13
G22
C23
5' end A3
srRNA3
Arg14
uL13
U2
5' end G1
srRNA2
Ala2
uL13
A1444
LSUB
Pro4
uL13
Phe3
uL13
A174
srRNA2
A1446
LSUB

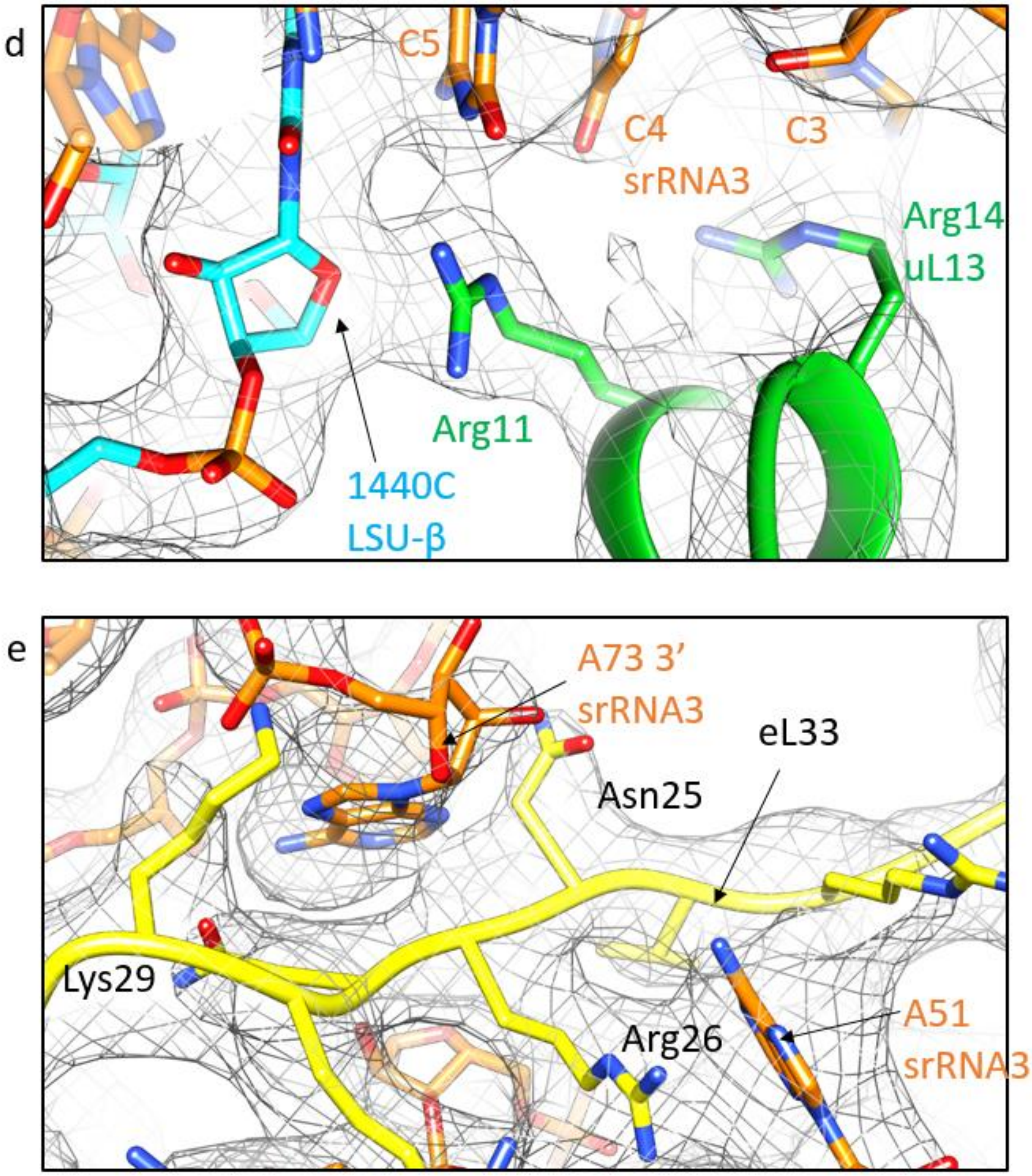

Supplementary Figure 11: Full models with density of selected interactions corresponding to regions from Figure 5. (a) Interaction of Arg6 of uL13 binding the 3’ end of srRNA2. (b) Sandwiching interaction involving Arg14 of uL13 and the 5’ end of srRNA2, G1, stacking with the 5’ end of srRNA3, A3. (c) Stacking between A174 of srRNA2 and A1444 and A1446 near the N-terminus of uL13. (d) Arg11 of uL13 binding C5 of srRNA3 and 1440C of LSU-β. (e) Backbone of eL33 between Asn25 and Arg26 binding the 3’ end of srRNA3, A73. Side chain of Arg26 binding A51 of srRNA3.

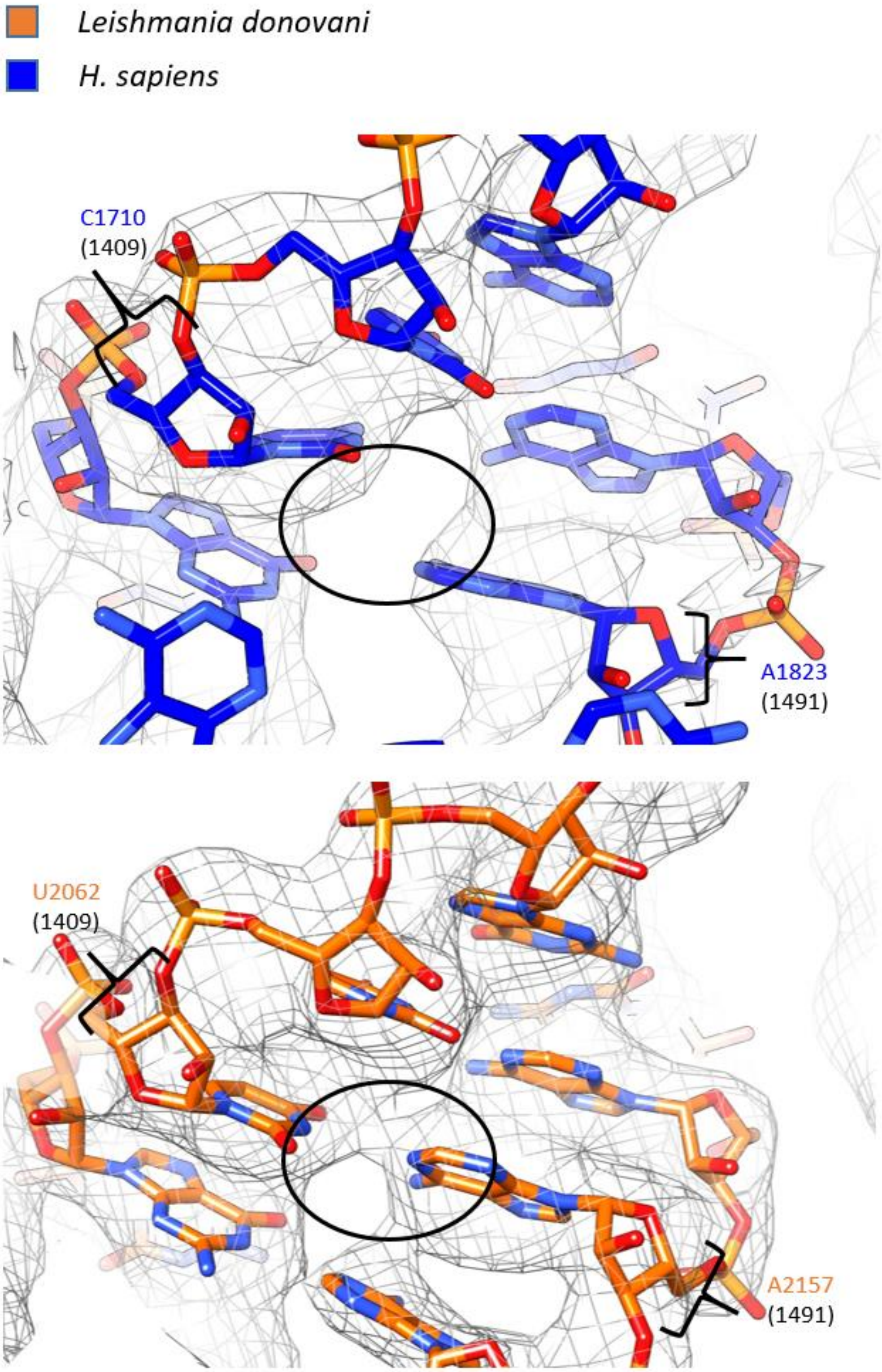

Supplementary Figure 12: Comparison of the 1409-1491 basepair in *L. donovani* and human ribosomes. Local resolution in the human structure is <3.5Å. Local resolution in the *L. donovani* structure is <3Å.

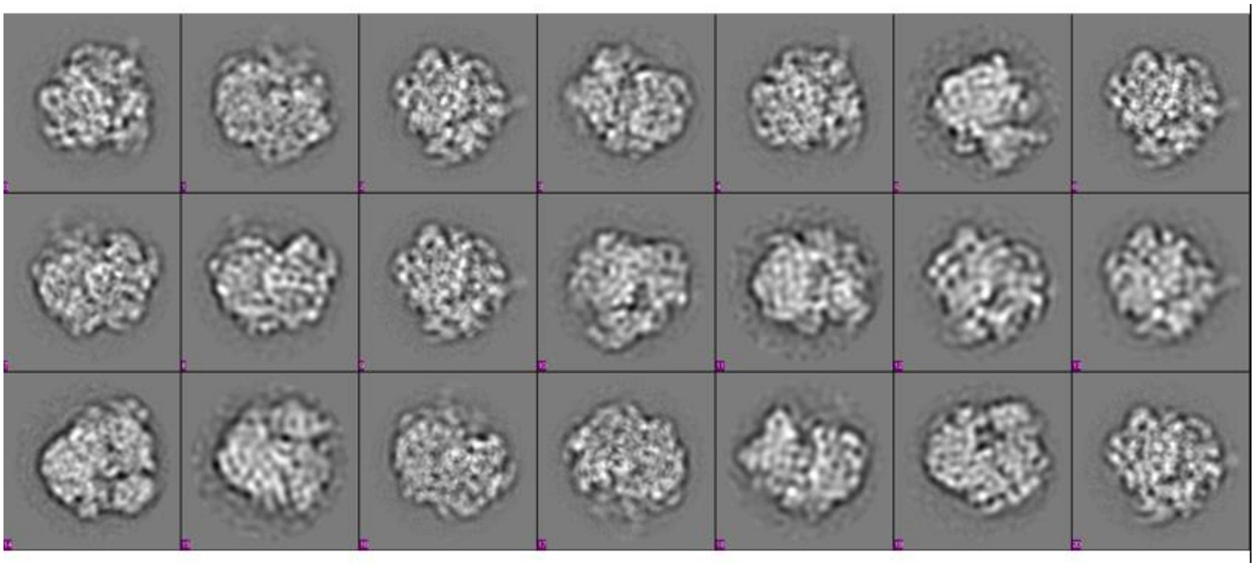

Supplementary Figure 13: Sample Class2D classes of the *L. donovani* ribosome.

Supplementary Table 1. Statistics of data processing and model building

Imaging parameters and model statistics for *L. donovani* and human ribosomes

	Leishmania donovani		Human	
Microscope	FEI Titan Krios		FEI Titan Krios	
Voltage (kV)	300		300	
Camera	Gatan K2 (counting Mode)		Gatan K2 (counting Mode)	
Energy-filter	None		GIF Quantum Energy Filter	
Magnification	62,422x		46,730x	
Å/pixel	0.801		1.07	
Defocus (µm)	1.1~2.5		1.0~3.63	
Total Dose (e/Å^2)	~25		~25	
Micrographs	4888		4091	
Particles selected	213,108		175,708	
Resolution (Å) (FSC≥0.143)	2.9 (averaged)		3.6 (averaged)	
**Refinement**				
	Large Subunit	Small Subunit	Large Subunit	Small Subunit
R_{work} (overall)	0.298 (30-2.9Å)	0.324 (30-2.9Å)	0.215 (50-3.6Å)	0.388 (50-3.6Å)
R_{free} (overall)	0.327 (30-2.9Å)	0.317 (30-2.9Å)	0.208 (50-3.6Å)	0.345 (50-3.6Å)
R_{work} (highest resolution zone)	0.496 (3.3-2.9Å)	0.497 (3.3-2.9Å)	0.280 (4.1-3.6Å)	0.507 (4.1-3.6Å)
R_{free} (highest resolution zone)	0.543 (3.3-2.9Å)	0.456 (3.3-2.9Å)	0.280 (4.1-3.6Å)	0.472 (4.1-3.6Å)
**Ramachandran plot**				
Most favored	91.70%	87.74%	90.31%	83.71%
Generously allowed	7.36%	9.33%	8.24%	13.16%
Disallowed regions	0.93%	2.93%	1.45%	3.13%

Supplementary Table 2. Summary of *L. donovani* LSU ribosomal protein models.

Protein name = protein names according to previously established nomenclature[4]. NCBI GI# =numbers of *L. donovani* protein sequence taken from the NCBI database. Size = number of amino acids. Range modeled = portions of the protein which have been modeled.

Protein name	NCBI GI#	Accession #	Size, aa	Range modeled, aa	Percentage modeled
uL2	154343633	XP_001567762	260	2-254	97%
uL3	157874311	XP_001685639	419	2-400	97%
uL4	398018739	XP_003862534	373	4-370	98%
uL5	398015105	XP_003860742	188	13-179	89%
uL6	398014912	XP_003860646	190	2-188	98%
eL6	157866918	XP_001682014	195	23-195	89%
eL8	398010596	XP_003858495	348	109-338	66%
uL13	322502489, 157866762	CBZ37572, XP_001681936	222	2-222	99%
eL13	389594167	XP_003722330	220	2-141, 172-214	97%
uL14	398023603	XP_003864963	139	3-139	99%
eL14	2500362	Q25278	175	2-169	96%
uL15	398023599	XP_003864961	145	2-145	99%
eL15	398023219	XP_003864771	204	2-204	99%
uL16	398009877	XP_003858137	213	2-212	99%
uL18	398023217	XP_003864770	305	5-133, 144-218, 229-265, 279-303	98%
eL18	398024852	XP_003865587	198	2-198	99%
eL19	398010301	XP_003858348	245	2-197	80%
eL20	398022973	XP_003864648	179	2-179	99%
eL21	398022682	XP_003864503	159	2-159	99%
eL22	398024594	XP_003865458	129	2-124	95%
uL22	398015811	XP_003861094	166	2-156	93%

uL23	262479179	ACY68622	145	26-145	83%
uL24	154338505	XP_001565477	143	3-122	84%
eL24	157876669	XP_001686679	124	1-65	52%
eL27	322501931	CBZ37014	134	2-134	99%
eL28	398011782	XP_003859086	147	2-147	99%
uL29	398017267	XP_003861821	127	2-127	99%
eL29	157877177	XP_001686919	70	2-70	99%
uL30	398016835	XP_003861605	252	25-252	90%
eL30	398022911	XP_003864617	104	9-102	90%
eL31	398023505	XP_003864914	183	74-183	60%
eL32	398023247	XP_003864785	133	2-130	97%
eL33	398022442	XP_003864383	144	13-144	92%
eL34	398024692	XP_003865507	168	2-125	74%
eL36	398023221	XP_003864772	105	3-101	94%
eL37	398021739	XP_003864032	83	2-82	98%
eL38	398017241	XP_003861808	83	2-76	90%
eL39	398019003	XP_003862666	51	2-51	98%
eL40	157873328	XP_001685176	128	77-128	41%
eL42	398021997	XP_003864161	106	2-97	91%
eL43	398024312	XP_003865317	92	2-92	99%

Supplementary Table 3. Summary of L. donovani SSU ribosomal protein models.

Protein name = protein names according to previously established nomenclature[4]. NCBI GI# =numbers of *L. donovani* protein sequence taken from the NCBI database. Size = number of amino acids. Range modeled = portions of the protein which have been modeled.

Protein name	NCBI GI#	Accession #	Size, aa	Range modeled, aa	Percentage modeled
eS1	398022945	XP_003864634	264	23-243	84%
uS2	157877540	XP_001687087	246	41-244	83%
uS3	154343872	XP_001567880	219	6-208	93%
uS4	398024172	XP_003865247	190	1-164	86%
eS4	398012258	XP_003859323	273	2-259	95%
uS5	157868060	XP_001682583	265	40-262	85%
eS6	157869182	XP_001683143	249	1-249	100%
uS7	157865716	XP_001681565	190	1-190	100%
eS7	389592391	XP_003721563	200	1-200	100%
eS8	398016233	XP_003861305	220	2-121, 155-220	85%
uS8	146079973	XP_001463917	130	2-130	99%
uS9	146089618	XP_001470429	149	10-149	94%
uS10	157871666	XP_001684382	116	15-116	88%
eS10	398024120	XP_003865221	153	38-129	60%
uS11	157871656	XP_001684377	144	8-143	94%
uS12	154337202	XP_001564834	143	2-143	99%
uS13	146103213	XP_001469509	153	6-153	97%
uS14	146092321	XP_001470262	57	20-57	67%
uS15	154335862	XP_001564167	151	2-142	93%
uS17	389603968	XP_003723129	173	11-159	86%
eS17	401425230	XP_003877100	143	3-123	85%
uS19	157869287	XP_001683195	152	16-136	80%
eS19	398022518	XP_003864421	179	19-163	81%

eS21	146079840	XP_001463877	164	4-86	51%
eS24	154345982	XP_001568928	137	4-129	92%
eS26	157872803	XP_001684928	112	1-104	93%
eS27	154335505	XP_001563991	86	5-86	95%
eS28	154339409	XP_001562396	87	20-87	78%
eS30	146093606	XP_001466914	66	7-62	85%
RACK1	13625467	AAK35068	312	1-308	99%

Supplementary References:

1 Scheres, S. H. RELION: implementation of a Bayesian approach to cryo-EM structure determination. *Journal of structural biology* **180**, 519-530, (2012).

2 Kucukelbir, A., Sigworth, F. J. & Tagare, H. D. Quantifying the local resolution of cryo-EM density maps. *Nature methods* **11**, 63-65, (2014).

3 Hashem, Y. *et al.* High-resolution cryo-electron microscopy structure of the Trypanosoma brucei ribosome. *Nature* **494**, 385-389, (2013).

4 Ban, N. *et al.* A new system for naming ribosomal proteins. *Current Opinion in Structural Biology* **24**, 165-169, (2014).
